# Plastome comparative genomics in maples resolves the infrageneric backbone relationships

**DOI:** 10.7717/peerj.9483

**Published:** 2020-07-13

**Authors:** Fabiola Areces-Berazain, Yixi Wang, Damien D. Hinsinger, Joeri S. Strijk

**Affiliations:** 1 Biodiversity Genomics Team, Plant Ecophysiology & Evolution Group, Guangxi Key Laboratory of Forest Ecology and Conservation, College of Forestry, Guangxi University, Nanning, Guangxi, China; 2 Alliance for Conservation Tree Genomics, Pha Tad Ke Botanical Garden, Luang Prabang, Laos; 3 Génomique Métabolique, Genoscope, Institut de Biologie François Jacob, Commisariat à l’Énergie Atomique (CEA), CNRS, Université Évry, Université Paris-Saclay, Évry, France; 4 State Key Laboratory for Conservation and Utilization of Subtropical Agro-bioresources, College of Forestry, Guangxi University, Nanning, Guangxi, China

**Keywords:** Acereae, Plastomes, Phylogenomics, Repeated sequences, SSRs

## Abstract

Maples (*Acer*) are among the most diverse and ecologically important tree genera of the north-temperate forests. They include species highly valued as ornamentals and as a source of timber and sugar products. Previous phylogenetic studies employing plastid markers have not provided sufficient resolution, particularly at deeper nodes, leaving the backbone of the maple plastid tree essentially unresolved. We provide the plastid genome sequences of 16 species of maples spanning the sectional diversity of the genus and explore the utility of these sequences as a source of information for genetic and phylogenetic studies in this group. We analyzed the distribution of different types of repeated sequences and the pattern of codon usage, and identified variable regions across the plastome. Maximum likelihood and Bayesian analyses using two partitioning strategies were performed with these and previously published sequences. The plastomes ranged in size from 155,212 to 157,023 bp and had structure and gene content except for *Acer palmatum* (sect. *Palmata*), which had longer inverted repeats and an additional copy of the *rps19* gene. Two genes, *rps2* and *rpl22*, were found to be truncated at different positions and might be non-functional in several species. Most dispersed repeats, SSRs, and overall variation were detected in the non-coding sequences of the LSC and SSC regions. Fifteen loci, most of which have not been used before in the genus, were identified as the most variable and potentially useful as molecular markers for barcoding and genetic studies. Both ML and Bayesian analyses produced similar results irrespective of the partitioning strategy used. The plastome-based tree largely supported the topology inferred in previous studies using cp markers while providing resolution to the backbone relationships but was highly incongruous with a recently published nuclear tree presenting an opportunity for further research to investigate the causes of discordance, and particularly the role of hybridization in the diversification of the genus. Plastome sequences are valuable tools to resolve deep-level relationships within *Acer*. The variable loci and SSRs identified in this study will facilitate the development of markers for ecological and evolutionary studies in the genus. This study underscores the potential of plastid genome sequences to improve our understanding of the evolution of maples.

## Introduction

*Acer* L., the maple genus, is the third most species-rich genus of trees in the northern hemisphere after *Quercus* L. and *Salix* L., and the fourth largest genus (in terms of species number) in the Sapindaceae (Acevedo-Rodríguez et al., 2011). It includes over 150 species distributed across all northern continents (De Jong, 2004). The greatest diversity is found in eastern Asia, particularly in China, where ca. 100 species have been reported (Xu et al., 2008). A few species extend into Central America (Guatemala and Honduras) and northern Africa, and only one extends beyond the Equator into Java and Sulawesi in Indonesia (De Jong, 1976).

Maples are popular and widely planted in temperate areas as decorative trees for their characteristic leaf shapes and showy fall foliage (Harris, 1975). Some species provide good-quality timber and hardwood for flooring, furniture, and many other applications (e.g., musical instruments, barrels, boxes, and woodenware) (Betts, 1959). The sugary sap of members of the *Acer saccharum* complex is used to produce the highly valued maple syrup (Ball, 2007).

Maples are among the most important components in the north-temperate deciduous forest biome. They can be found in a diverse range of habitats, from sea-level flatlands to higher than 3,000 m in the Himalayan forests. Several species, e.g., *Acer saccharum* Marshall, can be very abundant and are recognized as keystone species within their communities (Bishop et al., 2015; Minorsky, 2003). Many others are considered rare or threatened, mainly due to habitat loss and overexploitation. Fifty–four taxa (or about one–third of the genus) are included in the IUCN red list of maples with some category of threat (Gibbs & Chen, 2009).

The taxonomy of maples has always been considered as complicated due to the presence of extensive morphological variation in vegetative characters and the propensity of species to hybridize (De Jong, 1976; Grimm, Denk & Hemleben, 2007; Liao et al., 2010). This is reflected in the wide range of estimates of species numbers (and infraspecific taxa) reported in the literature, e.g., from 110 to over 155 spp. (Acevedo-Rodríguez et al., 2011; De Jong, 1976; De Jong, 2004; Xu et al., 2008). About a dozen classification systems have attempted to organize the species into subgenera, sections and series, mainly based on characters of leaves and the structure of inflorescences and flowers including the sex expression (De Jong, 1976; De Jong, 1994; De Jong, 2004; Momotani, 1962; Murray, 1970; Ogata, 1967; Pax, 1885; Pax, 1902; Pojarkova, 1933; Xu, 1996). The most recent system classifies the 156 species into 19 sections, six of which were subdivided into series (De Jong, 2004). However, the monophyly of some of these infrageneric groups is not or weakly supported by molecular data, and thus the classification of the genus remains only partially resolved (Harris et al., 2017; Li, 2011; Li, Yue & Shoup, 2006).

Phylogenetic studies in maples conducted over the past two decades (Ackerly & Donoghue, 1998; Grimm, Denk & Hemleben, 2007; Grimm et al., 2006; Harris, Frawley & Wen, 2017; Li, 2011; Li, Yue & Shoup, 2006; Renner et al., 2007; Renner et al., 2008; Suh, Heo & Park, 2000; Tian, Guo & Li, 2002; Zhang, Li & Li, 2010) have been based on a limited number of loci and/or have centered on specific taxonomic sections. Only recently, the first comprehensive phylogenomic study of *Acer* was published (Li et al., 2019). It was based on sequences of over 500 nuclear loci generated with hybrid enrichment for 65 species of *Acer*. Most of the 16 sections represented in their study were recovered as monophyletic with relatively high support, whereas three sections: *Acer*, *Lithocarpa*, and *Trifoliata*, were non-monophyletic.

Several *Acer* plastomes (e.g.,  Jia et al., 2016; Kim et al., 2019; Li et al., 2015; Wang, Chen & Zhang, 2017; Zhang et al., 2016) have been recently published. However, to our knowledge, no study has explored the structure and variation of the chloroplast genome across the genus. The plastid genome, which is maternally inherited in *Acer* (Corriveau & Coleman, 1988), has many advantages for phylogenetic inference and genetic studies over the nuclear and mitochondrial genomes (Daniell et al., 2016; Gitzendanner et al., 2018). A comparison between plastid and nuclear trees is relevant to our understanding of relationships in maples and could also provide evidence for hybridization and other processes in their evolutionary history. Here, we explore the diversity of plastomes in *Acer* as a first step towards generating a plastome-based tree for the genus. We assess the variation and compare structural features among 16 newly sequenced plastomes, each belonging to a different section in the genus. We investigate the effect of partitioning on tree inference using these and previously published plastid sequences and compare our results with the recently published nuclear tree. Finally, we discuss the utility of this genome as a source of information for genetic studies and phylogenetic inference in this valuable group of trees.

## Materials & Methods

### Taxon sampling

Leaf samples of 16 species of maples were obtained from cultivated trees in private and state gardens of Europe and China (Table S1). The identity of all samples was verified with experts from these institutions. Herbarium vouchers were deposited in the BGT herbarium, Guangxi University, China.

Each of the 16 species selected belongs to a different section in the genus (sensu De Jong, 2004), and 13 are the type of their respective section. Section *Hyptiocarpa* was recently merged with section *Rubra* (Harris et al., 2017) and was not considered in this study. *Acer oblongum*, placed in section *Pentaphylla* series *Trifida* by De Jong (2004), was included as a representative of section *Oblonga*, which was recognized by Xu et al. (2008) in Flora of China. Only sections *Spicata*, *Wardiana*, and *Macrophylla* could not be sampled.

For the phylogenomic reconstruction, plastome sequences from six additional species of maples were obtained from GenBank (see accessions in Table S1). We also retrieved sequences of the two species of *Dipteronia* (the sister genus of *Acer*), *Litchi chinensis* J. F. Gmel (Sapindaceae) and *Spondias mombin* L. (Anacardiaceae), which were incorporated as outgroups.

### DNA sequencing and plastome assembly and annotation

DNA extraction, library construction, and sequencing were performed by Annoroad Gene Technology (Beijing, PR China) Co., Ltd. Genomic DNA was isolated from frozen leaves using the DNAquick Plant system (TianGen Biotech, Beijing) following the manufacturer’s protocol. DNA degradation was assessed on 1% agarose gels. Purity and concentration were determined with a NanoPhotometer (Implen, USA) and a Qubit 2.0 fluorometer (Thermo Fisher Scientific, Massachusetts). Total DNA was fragmented to approximately 350 bp on an Ultrasonic Processor. Libraries were constructed using the NEBNext Ultra II DNA Library Prep Kit (Ipswich, Massachusetts) according to the manufacturer’s protocol, and subsequently diluted to 1 ng/µL. The final concentration and fragment sizes were verified on an Agilent 2100 Bioanalyzer (Agilent Technologies, California). Sequencing was performed on an Illumina HiSeq X Ten System (San Diego, California). Approximately one GB of 150 bp paired-end reads was generated for each sample.

Plastomes were assembled from cleaned reads using NOVOPlasty v. 2.7.2 (Dierckxsens, Mardulyn & Smits, 2017). When multiple contigs were obtained (rather than a single circularized assembly), we mapped them contigs against one of several *Acer* plastomes from GenBank (see accessions in Table S1) using Geneious v. 11.0.4 and merged the ones that overlapped. Because in several instances the assemblies resulted in short non-overlapping contigs, we mapped our reads against the contigs to extend their ends until the gap was closed. Mapping was performed with medium-low sensitivity for 100 iterations.

The annotation of the assemblies was performed with GeSeq (Tillich et al., 2017). We selected ARAGORN as third party tRNA annotator and the plastome sequence of *Acer miyabei* Maxim. subsp. *miaotaiense* (P.C. Tsoong) A.E. Murray (NC_030343) as the reference genome. The annotated sequences were aligned and verified with published *Acer* plastomes using MAFFT v. 7.450 (Katoh & Standley, 2013) in Geneious and submitted to GenBank (Table S1).

### Plastome comparison

The boundaries between the four plastome regions were inspected with the online tool IRscope (Amiryousefi, Hyvonen & Poczai, 2018), which allows visualizing the position of genes in the vicinity of these sites across species. We compared sequences and identified regions of variability with mVISTA (Frazer et al., 2004) using the annotated plastome of *Acer acuminatum* Wall. ex D. Don as a reference. Additionally, we performed a sliding window analysis as implemented in DnaSP v. 6.12.03 (Rozas et al., 2017) to locate genomic regions with high levels of variation. The alignment of the 16 *Acer* plastomes obtained with MAFFT (with default settings) was used as input file. The window length and step size were set to 600 bp and 100 bp, respectively. Using window’s position, we identified regions with 60 or more variable (polymorphic) sites (S). These regions were extracted from the alignment and analyzed individually to estimate their number of variable sites and parsimony-informative sites.

A codon usage analysis for protein-coding genes was also performed in DnaSP. We extracted the CDSs using Geneious and calculated the codon frequency and the relative synonymous codon usage (RSCU) values as a measure of codon usage bias (Sharp, Tuohy & Mosurski, 1986).

REPuter (Kurtz et al., 2001) was used to detect various types of repeated sequences (forward, reverse, complement, and palindromic) with a minimum size of 25 bp and sequence identity greater than 90%. Simple sequence repeats (SSRs) were identified with MISA-web (Beier et al., 2017). We used the default values of 10, 6, 5, 5, 5, and 5 to set the minimum number of repetitions for mono, di-, tri-, tetra-, penta-, and hexanucleotide repeats, respectively.

### Phylogenomic reconstruction

Phylogenomic reconstruction was performed on a dataset consisting of 22 *Acer* plastomes (six retrieved from GenBank), and four outgroup species (Table S1). We aligned the annotated sequences using MAFFT with default parameters and then removed one inverted repeat. Maximum likelihood and Bayesian analyses were performed on this data set using two partitioning strategies to explore the effect of partitioning on tree topology. In the first one, the alignment was fully partitioned into coding and non-coding regions with protein-coding genes further divided by codon position. We used Geneious to extract the protein-coding sequences to define the codon positions in the data blocks and then concatenated these with the non-protein-coding regions. In the second strategy, the whole plastome was treated as a single partition (i.e., unpartitioned).

We used PartitionFinder2 (Lanfear et al., 2016) to select the best partitioning scheme and best-fit substitution models for the partitioned dataset. Branch lengths were set as ‘linked’ and the AICc was used for model selection. The search was performed using ‘rcluster’, a fast algorithm recommended for large datasets with a high number of partitions (Lanfear et al., 2014). We defined 324 data blocks which were reduced to 93 subsets in the best-fit scheme. These subsets and their corresponding substitution models were specified in both ML and Bayesian partitioned analyses. The unpartitioned analysis was run using GTR+I+G as a substitution model, which was selected using both PartitionFinder2 and ModelTest-NG (Darriba et al., 2019). The maximum likelihood analysis was performed in RaxML-NG (Kozlov et al., 2019) with 1,000 bootstrap replicates. The Bayesian analysis was conducted in MrBayes v. 3.2.7 (Ronquist et al., 2012) on the CIPRES Science Gateway (Miller, Pfeiffer & Schwartz, 2010). We ran two runs of 100 million generations and four chains, sampling trees every 4,000 generations and discarding the 20% as burn-in. Tracer v. 1.6.0 (Rambaut et al., 2018) was used to verify that both runs reached stationarity and converged on the same distribution.

**Table 1:** Main features of the chloroplast genomes generated in this study.

**Species**	**Section**	**Total length (bp)**	**LSC (bp)**	**SSC (bp)**	**IR (bp)**	**GC%**				**# of coding loci**	**# of tRNA loci**	**# of rRNA loci**
						**Total**	**LSC**	**SSC**	**IR**			
*Acer acuminatum*	*Arguta*	155,548	85,358	18,046	26,072	37.9	36.1	32.1	42.9	89	38	8
*Acer carpinifolium*	*Indivisa*	155,212	85,448	17,724	26,020	38.0	36.2	32.5	43.0	89	38	8
*Acer glabrum*	*Glabra*	156,373	86,034	18,211	26,064	37.9	36.0	32.2	42.9	89	38	8
*Acer maximowiczianum*	*Trifoliata*	156,082	85,865	18,147	26,035	37.9	36.1	32.3	42.9	89	38	8
*Acer micranthum*	*Macrantha*	156,399	86,147	18,128	26,062	37.9	36.0	32.1	42.9	89	38	8
*Acer negundo*	*Negundo*	155,938	85,678	18,092	26,084	37.9	36.1	32.3	42.9	89	38	8
*Acer nipponicum*	*Parviflora*	156,225	85,823	18,232	26,085	37.8	35.9	32.0	42.9	89	38	8
*Acer oblongum*	*Oblonga*	155,686	85,665	17,821	26,100	38.0	36.1	32.4	42.9	89	38	8
*Acer palmatum* var. *palmatum*	*Palmata*	157,023	85,342	18,167	26,757	37.9	36.0	32.2	42.8	90	38	8
*Acer pentaphyllum*	*Pentaphylla*	156,220	85,938	18,148	26,067	37.9	36.0	32.3	42.9	89	38	8
*Acer pilosum*	*Pubescentia*	155,586	85,313	18,139	26,067	38.0	36.2	32.1	42.9	89	38	8
*Acer platanoides*	*Platanoidea*	156,385	86,098	18,107	26,090	37.9	36.0	32.1	42.9	89	38	8
*Acer pseudoplatanus*	*Acer*	155,933	85,812	17,971	26,075	37.9	36.1	32.2	42.9	89	38	8
*Acer rubrum*	*Rubra*	155,683	85,383	18,086	26,107	37.9	36.1	32.2	42.9	89	38	8
*Acer sterculiaceum* subsp. *sterculiaceum*	*Lithocarpa*	156,258	86,014	18,048	26,098	38.0	36.1	32.3	42.9	89	38	8
*Acer tataricum* subsp. *ginnala*	*Ginnala*	155,667	85,404	18,061	26,101	38.0	36.2	32.4	42.9	89	38	8

**Notes.**

IR inverted repeat LSC large single copy SSC small single copy

## Results

### Size and composition of *Acer* plastomes

The 16 plastomes generated in this study varied in length from 155,212 bp in *Acer carpinifolium* Siebold & Zucc. to 157,023 bp in *Acer palmatum* Thunb.var. *palmatum* (Table 1). All exhibited the typical tetrapartite organization, with the large single copy (LSC), small single copy (SSC), and inverted repeat (IR) regions ranging from 85,313 to 86,147, 17,724 to 18,232, and 26,020 to 26,757 bp, respectively. The largest genome size of *A. palmatum* is due to its expanded IRs relative to the other species.

The GC content was similar for all species (37.8–38%). As is common in plastomes of seed plants, the IRs had the highest GC content (42.8–43%) due to the presence of the GC-rich rRNA genes, whereas the SSC region had the lowest values (35.9–36.2%, Table 1).

All plastomes contained 117 different genes, of which 19 are duplicated for a total of 136 genes (Table 2). *Acer palmatum* is the only species with 137 genes due to an entire additional copy of the ribosomal *rps19*. Eighty-one of the 117 genes are protein-coding genes, 31 are transfer RNA genes, and four are ribosomal RNA genes. One gene, *infA*, appears as a pseudogene and is likely to be non-functional in all species. Sixteen genes contain a single intron, and two have two introns (Table 2).

**Table 2:** List of genes annotated in the plastomes of *Acer* species generated in this study. A total of 136 genes are present in all species except for *A. palmatum* which has two copies of the ribosomal protein gene *rps19*. Genes marked with one asterisk contain one intron; two asterisks indicate two introns.

**Gene class**	**# of genes**	**Gene name**
Photosystem I	5	*psaA*, *psaB*, *psaC*, *psaI*, *psaJ*
Photosystem II	15	*psbA*, *psbB*, *psbC*, *psbD*, *psbE*, *psbF*, *psbH*, *psbI*, *psbJ*, *psbK*, *psbL*, *psbM*, *psbN*, *psbT*, *psbZ*
Photosystem assembly factors	2	*ycf3* **, *ycf4*
Cytochrome b/f complex	6	*petA*, *petB* *, *petD* *, *petG*, *petL*, *petN*
ATP synthase complex	6	*atpA*, *atpB*, *atpE*, *atpF* *, *atpH*, *atpI*
NADH dehydrogenase complex	12	*ndhA* *, *ndhB* * (×2), *ndhC*, *ndhD*, *ndhE*, *ndhF*, *ndhG*, *ndhH*, *ndhI*, *ndhJ*, *ndhK*
Large subunit of RuBisCO	1	*rbcL*
Maturase	1	*matK*
RNA polymerase subunits	4	*rpoA*, *rpoB*, *rpoC1* *, *rpoC2*
Small subunit ribosomal proteins	14 (15)	*rps2*, *rps3*, *rps4*, *rps7* (×2), *rps8*, *rps11*, *rps12* * (×2), *rps14*, *rps15*, *rps16* *, *rps18*, *rps19* (×2 in *A. palmatum*)
Large subunit ribosomal proteins	11	*rpl2* * (×2), *rpl14*, *rpl16* *, *rpl20*, *rpl22*, *rpl23* (×2), *rpl32*, *rpl33*, *rpl36*
Subunit of acetyl-CoA-carboxylase	1	*accD*
Subunit of Clp-protease	1	*clpP* **
Translation initiation factor	1	*infA*Ψ
Inner envelope membrane protein	1	*cemA*
Cytochrome c biogenesis protein	1	*ccsA*
Genes of unknown function	8	*orf42* (×2), *ycf1* , *ycf1a*, *ycf2* (×2), *ycf15* (×2)
Ribosomal RNAs	8	*rrn4.5* (×2), *rrn5* (×2), *rrn16* (×2), *rrn23* (×2)
Transfer RNAs	38	*trnA-UGC* * (×2), *trnC-GCA*, *trnD-GUC*, *trnE-UUC*, *trnF-GAA*, *trnfM-CAU*, *trnG-GCC*, *trnG-UCC* *, *trnH-GUG*, *trnI-CAU* (×2), *trnI-GAU* * (×2), *trnK-UUU* *, *trnL-CAA* (×2), *trnL-UAA* *, *trnL-UAG*, *trnM-CAU*, *trnN-GUU* (×2), *trnP-GGG*, *trnP-UGG*, *trnQ-UUG*, *trnR-ACG* (×2), *trnR-UCU*, *trnS-GCU*, *trnS-GGA*, *trnS-UGA*, *trnT-GGU*, *trnT-UGU*, *trnV-GAC* (×2), *trnV-UAC* *, *trnW-CCA*, *trnY-GUA*
TOTAL	136 (137)	

The size of homologous genes was very similar across species except for a few cases. For example, the *rps2* gene was found to be truncated at different positions in 11 species due to mutations leading to premature stop codons. It was significantly shorter (72–264 bp) and perhaps non-functional in *A. acuminatum*, *A. carpinifolium*, *A. micranthum*, *A. negundo*, *A. nipponicum*, *A. palmatum*, *A. pilosum*, *A. pseudoplatanus*, *A. rubrum*, and *A. tataricum*. The other six species had lengths of 588 and 633 bp. The *rpl22* gene was also notably shorter (183–225 bp) in two species (*A. sterculiaceum* and *A. pentaphyllum*) compared to the rest whose length ranged from 480 and 498 bp.

**Figure 1:**
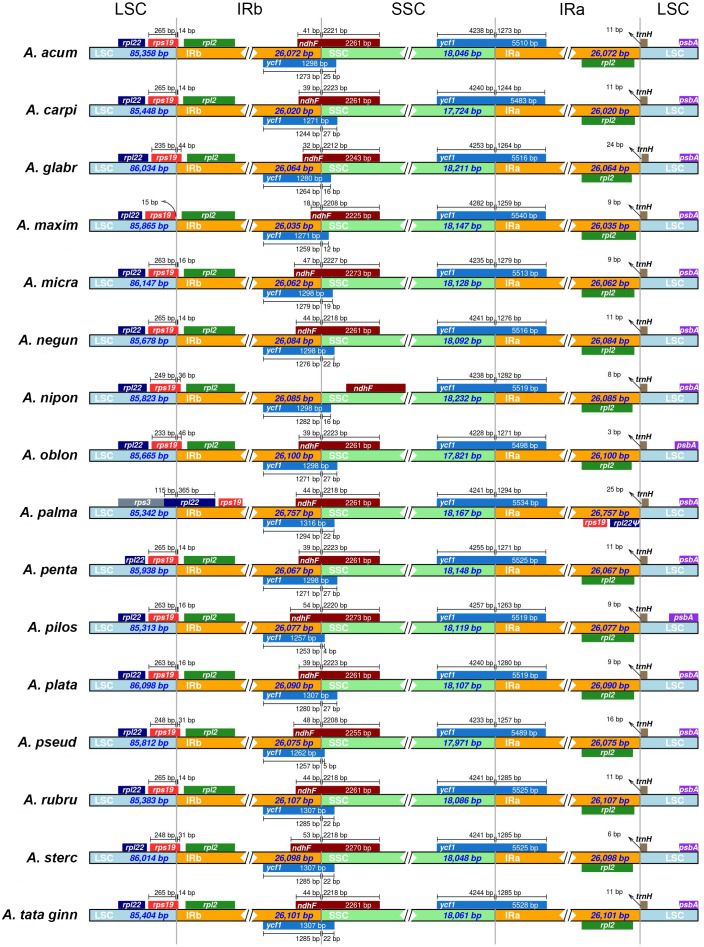
Schematic diagram of the boundaries between the four regions (LSC, large single copy; SSC, short single copy; IR, inverted repeat) in the 16 plastid genomes sequenced in this study. See Table 1 for full species names.

In all species, with the exception of *A. maximowiczianum* and *A. palmatum*, the boundary between the LSC and IRb regions is located in the 3′ end of the *rps19* gene (Fig. 1). In *A. maximowiczianum*, the entirety of *rps19* lies in the LSC region, whereas in *Acer palmatum*, it is found within the inverted repeats. This species is atypical in that the boundary LSC-IRb is situated in the *rpl22* gene, which results in a duplicated fragment of this gene in the IRa adjacent to the junction with the LSC region (Fig. 1).

A truncated but seemingly functional copy of the *ycf1* gene is present in the IRb of all plastomes extending to various lengths into the SSC region. The *ndhF* gene also spans the boundary SSC-IRb in the opposite direction, partially overlapping with the 3′ end of *ycf1*. The only exception is *Acer nipponicum*, in which the entire *ndhF* sequence lies within the SSC region (Fig. 1).

### Sequence variation

**Figure 2:**
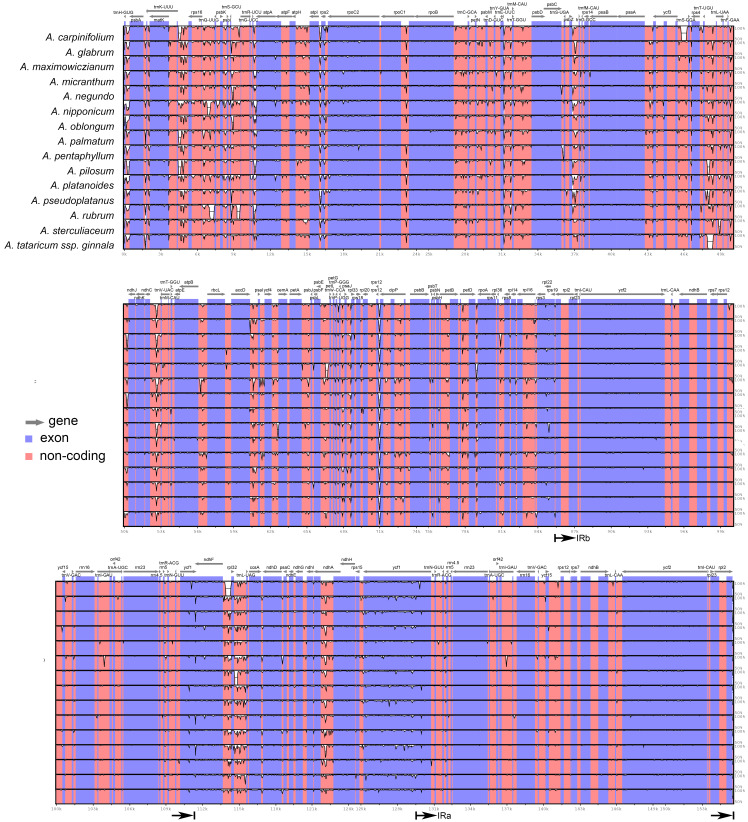
mVISTA alignment comparing the plastomes of *Acer* species against *A. acuminatum*. The vertical scale to the right shows the percent of identity (50–100%) between the species compared. The black arrows indicate the boundaries of the inverted repeats. Coding regions are indicated in blue and non-coding regions in pink.

**Figure 3:**
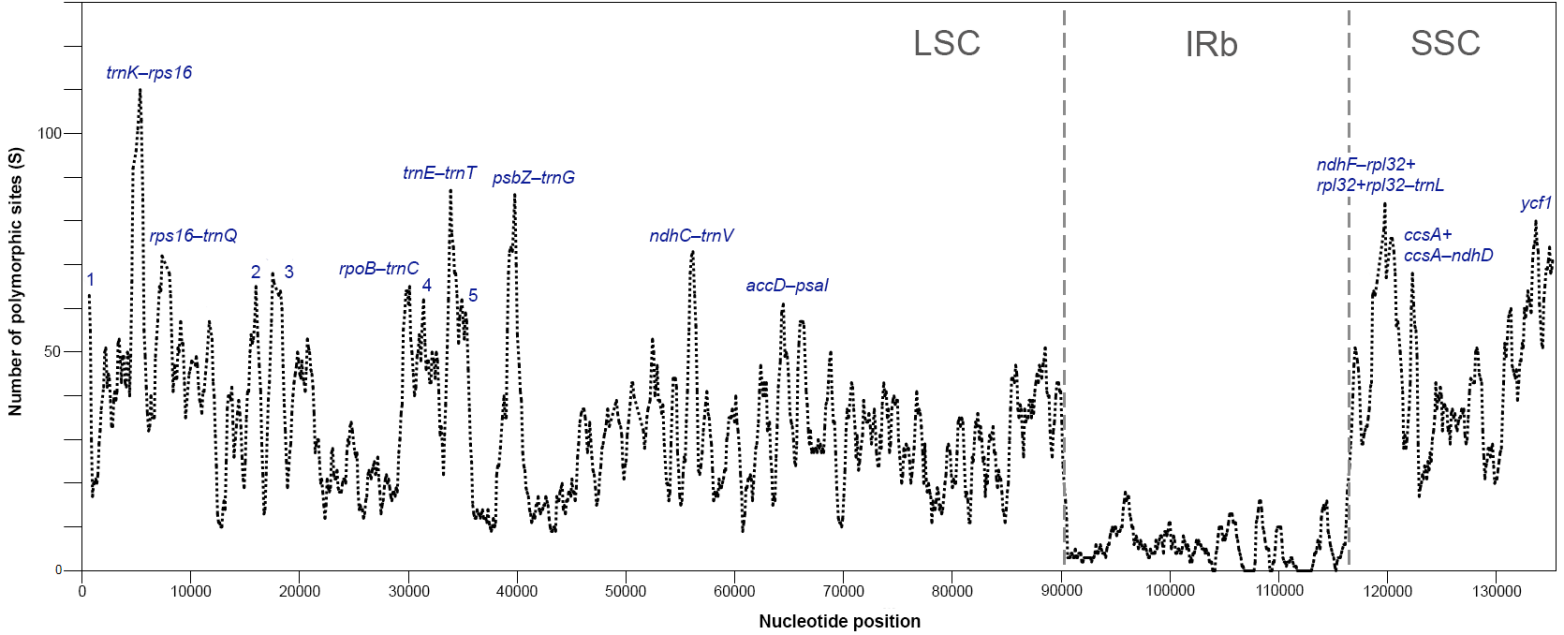
Sliding window analysis of polymorphic sites (S) for plastomes of 16 species of *Acer* (window length: 600 bp, step size: 100 bp). Regions with the highest number of S are indicated. 1: *trnH*-*psbA*, 2: *atpH-atpI*, 3: *atpI-rps2+rps2+rps2-rpoC2*, 4: *petN-psbM*, 5: *trnT -psbD* (see Table 3 for details). One inverted repeat not shown.

The plastomes of *Acer* are very similar and largely conserved as shown in the mVISTA alignment, with most of the variation found in the non-coding sequences of the LSC and SSC regions (Fig. 2). Fifteen most variable regions (S ≥ 60) were identified in the sliding window analysis. All but one, the *ycf1* gene, were or contained intergenic spacers. Three compound regions, formed by one protein-coding gene (*rps2*, *rpl32* and *ccsA*) plus one or both flanking spacers, were detected (Table 3, Fig. 3). When these regions were analyzed separately, the number of polymorphic sites varied from 36 (*ccsA–ndhD*) to 438 (*ycf1*). The percent of variability and parsimony-informative sites ranged from 5.3% (*rps16–trnQ-UUG*) to 16.7% (*ccsA–ndhD*), and from 1.0% (*accD–psaI* and *rps16–trnQ-UUG*) to 8.8% (*ccsA–ndhD*), respectively (Table 3). The five most variable regions, both in percent of variability and total number of parsimony-informative sites were the spacers *ccsA–ndhD, psbZ–trnG-GCC, ndhC–trnV-UAC, trnE-UUC–trnT-GGU*, and the *ycf1* gene. This gene exhibited greater variation in the SSC portion of the genome (9.7%) compared to the IR portion (1.1%).

**Table 3:** List of most variable regions in *Acer* plastomes.

**Region**	**Length (bp)**	**Aligned length (bp)**	**No. of variable (polymorphic) sites**	**No. of parsimony- informative sites**
*ycf1* (SSC portion)	4205–4282	4361	423 (9.7%)	93 (2.1%)
*ycf1* (whole)	5484–5541	5673	438 (7.7%)	99 (1.7%)
*ndhF–rpl32+rpl32+*	1529–2061	2453	146 (5.9%)	42 (1.7%)
*rpl32–trnL-UAG*				
*rpl32–trnL-UAG*	833–1159	1334	98 (7.3%)	30 (2.2%)
*trnT-GGU–psbD*	1454–1511	1565	119 (7.6%)	21 (1.3%)
*trnK-UUU–rps16*	776–1022	1127	118 (10.5%)	22 (1.6%)
*atpH–atpI*	1117–1178	1274	103 (8.1%)	14 (1.1%)
*trnE-UUC–trnT-GGU*	761–818	866	95 (11%)	18 (2.1%)
*ccsA+ccsA–ndhD*	1150–1162	1173	93 (7.9%)	24 (2.0%)
*ccsA–ndhD*	193–205	216	36 (16.7%)	19 (8.8%)
*rpoB–trnC-GCA*	1161–1189	1265	92 (7.3%)	15 (1.2)
*ndhC–trnV-UAC*	884–927	1040	84 (8.1%)	39 (3.75)
*atpI–rps2+rps2* +*rps2–rpoC2*	790–1195	1243	76 (6.1%)	18 (1.4%)
*petN–psbM*	762–796	867	73 (8.4%)	15 (1.7%)
*psbZ–trnG-GCC*	516–604	685	71 (10.4%)	27 (3.9%)
*rps16–trnQ-UUG*	783–1120	1252	66 (5.3%)	13 (1.0%)
*accD–psaI*	607–707	759	59 (7.8%)	8 (1.0%)
*trnH-GUG–psbA*	272–435	529	52 (9.8%)	7 (1.3%)

### Codon usage

The total number of codons (including stop codons) of the protein-coding regions of the plastomes ranged from 26,678 in *A. psudoplatanus* to 26,865 in *A. glabrum*. Codon frequency and RSCU values were very similar across species (Table 4, Table S2). The most frequent codons were AUU-Ile (1084-1112), AAA-Lys (1,060–1,079), and GAA-Glu (1,011–1,024), the three accounting for about 11% of the total number of codons in all species. The three least frequent were UGC-Cys (81–86), AGC-Ser (124–131), and CGC-Arg (127–136). The most commonly specified amino acids were leucine and isoleucine, encoded by about 10% and 8% of codons, respectively (Table 4). Codons that have T or A in their third position had RSCU >1, whereas codons ending in C or G had RSCU <1 indicating a strong bias in favor of codons ending with T and A. The only exceptions to this pattern were UUG-Leu with RSCU values between 1.20 and 1.23, CUA-Leu with 0.83–0.85, and AUA-Ile with 0.92–0.94. The two codons with the highest RSCU values (both with 1.78–1.81) were UUA-Leu and AGA-Arg, and the two with the lowest were AGC-Ser (0.36–0.38) and UAC-Tyr (0.37–0.39) (Table 4, Table S2).

**Table 4:** Codon usage in *Acer* plastomes.

**Codon**	**Aa**	**No.**	**RSCU**	**Codon**	**Aa**	**No**	**RSCU**	**Codon**	**Aa**	**No**	**RSCU**	**Codon**	**Aa**	**No**	**RSCU**
UUU	Phe	999-1012	1.28-1.29	UCU	Ser	548-556	1.58-1.61	UAU	Tyr	771-788	1.61-1.63	UGU	Cys	211-224	1.42-1.47
UUC		556-565	0.71-0.72	UCC			348-358	1.00-1.03		UAC	177-187	0.37-0.39		UGC	81-86	0.53-0.58
UUA	Leu	835-853	1.78-1.81	UCA			433-447	1.25-1.28	UAA*	Stop	48-52	1.62-1.75	UGA*	Stop	14-17	0.47-0.57
UUG			563-576	1.20-1.23		UCG	196-206	0.57-0.60	UAG*		22-25	0.74-0.84	UGG	Trp	452-460	1.00
CUU			576-588	1.23-1.25	CCU	Pro	411-421	1.46-1.50	CAU	His	491-508	1.48-1.50	CGU	Arg	317-323	1.16-1.19
CUC			213-223	0.45-0.47	CCC			227-238	0.81-0.85		CAC	164-173	0.50-0.52		CGC		127-136	0.47-0.50
CUA			389-400	0.83-0.85	CCA			310-323	1.11-1.16	CAA	Gln	716-728	1.52-1.54		CGA		360-376	1.33-1.37
CUG		207-218	0.44-0.46	CCG	151-164		0.54-0.58	CAG	218-227	0.46-0.48		CGG	139-148		0.51-0.55
AUU	Ile	1084-1112	1.45-1.47	ACU	Thr	513-523	1.52-1.56	AAU	Asn	958-977	1.50-1.52	AGU	Ser	398-412	1.15-1.19
AUC			445-460	0.59-0.61		ACC		258-269		0.77-0.81	AAC	307-318		0.48-0.50	AGC	124-131	0.36-0.38
AUA		696-709	0.92-0.94	ACA			396-406	1.18-1.20	AAA	Lys	1060-1079	1.47-1.48	AGA	Arg	481-492	1.78-1.81
AUG	Met	611-623	1.00	ACG		155-168	0.46-0.50	AAG	374-384		0.52-0.53	AGG	180-191		0.67-0.70
GUU	Val	524-534	1.43-1.46	GCU	Ala	608-625	1.72-1.75	GAU	Asp	838-862	1.57-1.58	GGU	Gly	586-598	1.28-1.30
GUC			185-191	0.50-0.52		GCC		228-238		0.64-0.67	GAC	226-233		0.42-0.43	GGC		185-193	0.40-0.42
GUA			539-549	1.47-1.50		GCA		378-388	1.07-1.09	GAA	Glu	1011-1024		1.47-1.48	GGA		720-733	1.57-1.59
GUG		199-206	0.55-0.56	GCG		187-196	0.52-0.55	GAG	356-370	0.52-0.53		GGG		326-340	0.71-0.74

**Notes.**

Aa Amino acid No Number (minimum-maximum values) RSCU Relative synonymous codon usage (minimum-maximum values)

### Repeat sequence analysis

**Figure 4:**
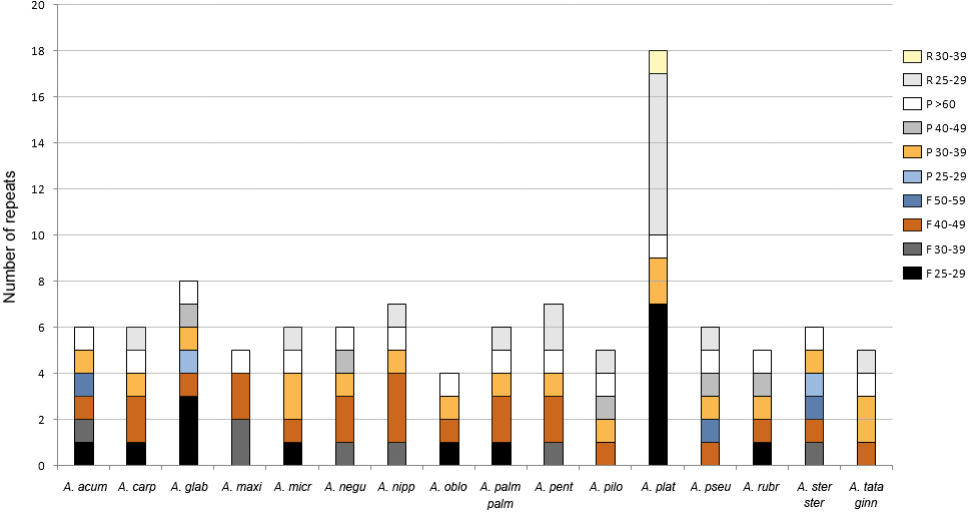
Number of repeats found in *Acer.* plastomes using REPuter. Only repeats ≥25 bp were considered. F, forward; P, palindrome; R, reverse. See Table 1 for full species names.

The analysis with REPuter detected between four and 18 repeats with a length ≥25 bp in all plastomes (Fig. 4, Table S3). *Acer platanoides* L. had the highest number while all other species ranged between four and eight repeats. The most common type were forward sequences in the range of 40–49 bp followed by palindromes between 30–39 bp (Fig. 4). No complement repeats were found in any of the plastomes analyzed.

The majority (about 67%) of these repeated sequences were identified in the intergenic spacers of the LSC and SSC regions; nearly a third were detected in the most variable spacers (Table 3). Only two were located in intergenic spacers of the IRs in one species (*Acer glabrum* Torr.). The remaining repeats were detected in five protein-coding genes (*psaA*, *psaB*, *rpoC1*, *rpl22*, *rpl32*) and one transfer RNA gene (*trnS-GGA*). The repeats found in the *rpoC1* gene were located in its intron sequence (Table S3).

**Figure 5:**
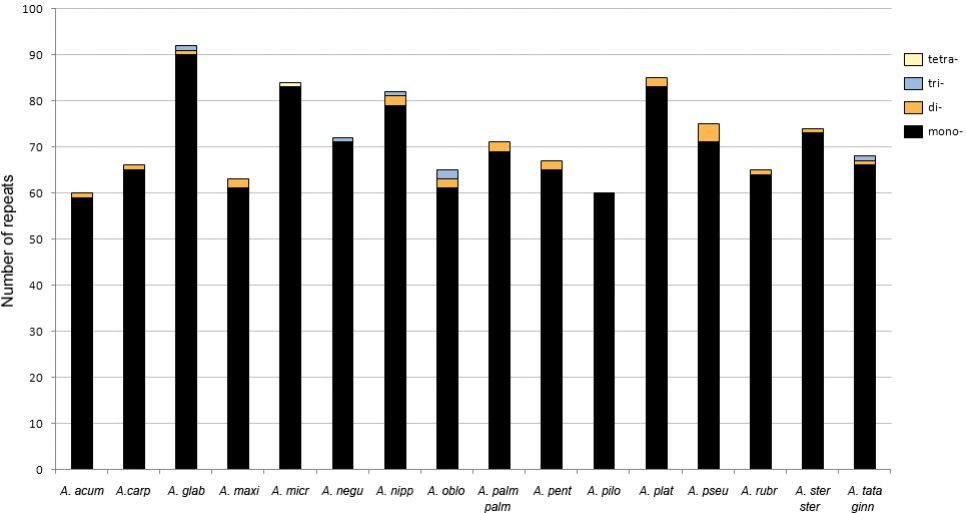
Number of simple sequence repeats (SSRs) found in *Acer* plastomes using MISA-web. See Table 1 for full species names.

The number of SSR loci ranged from 60 in the plastomes of *A. acuminatum* and *Acer pilosum* Maxim. to 92 in *A. glabrum*. Ninety-seven percent were mononucleotide repeats of up to 28 bp long. A few dinucleotide repeats were found in 13 of the 16 species, whereas tri- and tetranucleotide repeats were detected in only five and one species, respectively (Fig. 5). The most frequent motifs were (A/T)10 and (A/T)11, which collectively accounted for up to 78–79% of all SSRs in some species. C and G repeats were rare, with only one to three per species (Table S4).

**Figure 6:**
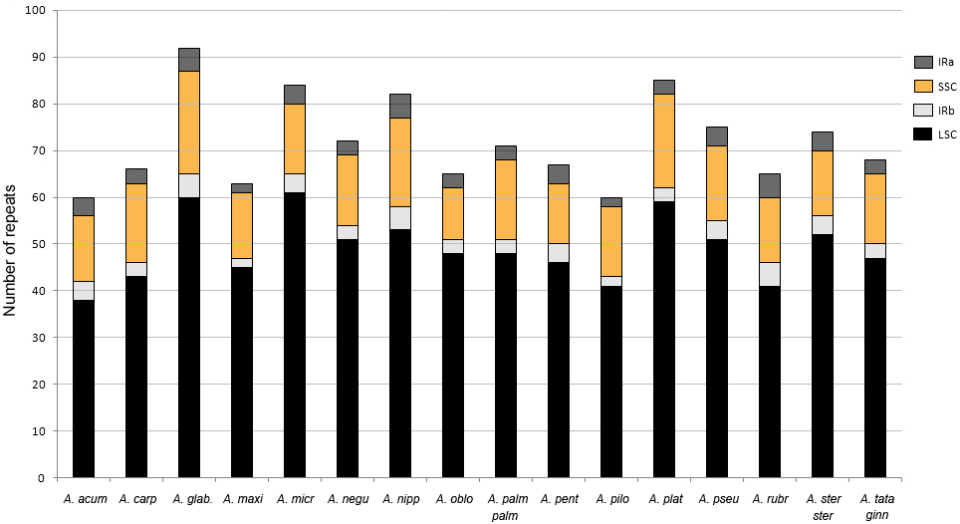
Number of simple sequence repeats (SSRs) found in *Acer* plastomes according to their location. IR, inverted repeat; LSC, large single copy, SSC, short single copy. See Table 1 for full species names.

The percentage of the cp genome containing SSRs varied from 0.48 to 0.94% (average = 0.7%). Most loci (90%) were identified in the LSC and SSC regions (Fig. 6). About 58% were located in the intergenic spacers of the genome, whereas 42% were detected in 25 different genes. The *ycf1* gene had the highest number of SSRs with six to ten per species (Table S5).

### Phylogenomic reconstruction

**Figure 7:**
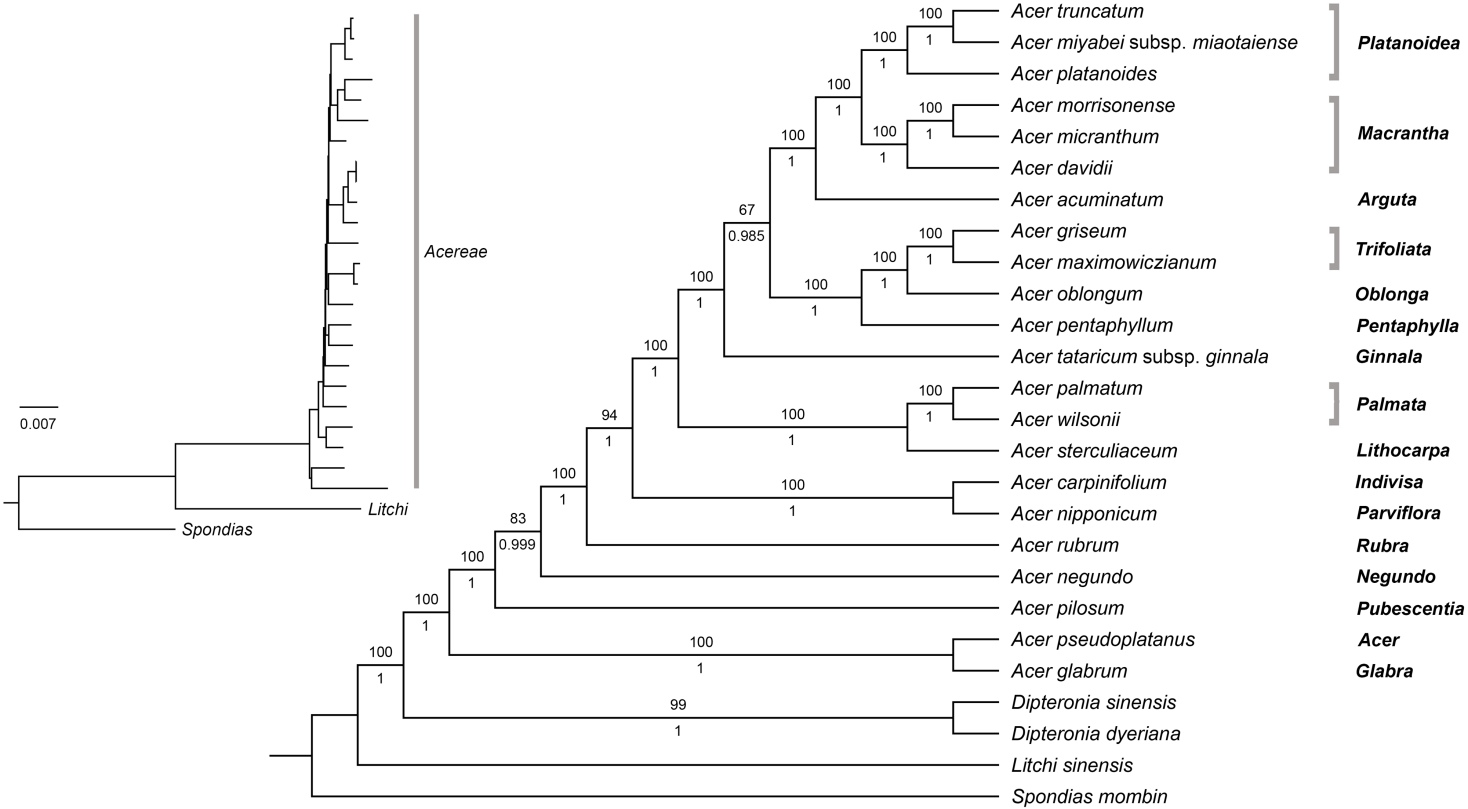
Maximum likelihood tree for *Acer.* plastomes inferred with RaxML-NG. The topology is identical to the Bayesian 50% majority rule consensus tree. Numbers above and below branches are bootstrap values and posterior probabilities, respectively. Names on the right are *Acer* sections.

The plastome data set (with one inverted repeat removed) consisted of 153,383 aligned sites, of which 20,478 (13.4%) were variable, and 5,774 were parsimony-informative. Pairwise percent identity for all species is provided in Table S6. Both ML and Bayesian analyses on the fully partitioned and the unpartitioned dataset produced trees with identical topologies, very short internal branches, and similar support values (Fig. 7).

All analyses provided maximum support for the monophyly of *Acer* and its sister relationship to *Dipteronia*. Relationships within the genus were fully resolved with strong support; only two branches were weakly to moderately supported in the ML analysis with BS values of 67 and 83%, respectively (Fig. 7). The earliest diverging lineages within the genus included species of sections *Acer* (*Acer pseudoplatanus* L.), *Glabra* (*A. glabrum*), *Pubescentia* (*A. pilosum*), *Negundo* (*Acer negundo* L.) and *Rubra* (*Acer rubrum* L.). The remaining species were grouped into three mutually exclusive clades, one consisting of *A. nipponicum* and *A. carpinifolium* (sections *Parviflora* and *Indivisa*, respectively), another formed by species of sections *Palmata* and *Lithocarpa*, and the other composed by members of the remaining sections. Within this clade, *A. tataricum* subsp. *ginnala* is sister to all other species. Section *Platanoidea* was recovered as sister to section *Macrantha* with *A. acuminatum* (section *Arguta*) as sister to this clade. The species of sections *Trifoliata*, *Oblonga*, and *Pentaphylla* formed another clade that is sister to the *Platanoidea-Macrantha-Arguta* clade. All sections represented by more than one species were recovered as monophyletic with high support (Fig. 7).

## Discussion

### Plastome composition and variable regions

The plastomes of *Acer* generated in this study are very homogeneous in size and structure as well as gene content. Of the 16 species sampled, *A. palmatum* (section *Palmata*) is the only one that differed from the rest by having much longer IRs and an extra copy of the *rps19* gene (Fig. 1, Table 1). A survey of plastomes of other members of section *Palmata*, including *Acer wilsonii* Rehder (MG012225) and 16 additional species assembled by the authors (Data S2) shows that all share these IRs of about 700 bp longer. Expanded IRs containing whole copies of *rps19* are also present in *Dipteronia* (NC_029338, NC_031899), *Dodonaea viscosa* Jacq. (NC_036099), *Dimocarpus longan* Lour. (NC_037447), *Koelreuteria paniculata* Laxm. (NC_037176), *Litchi sinensis* (NC_035238), *Sapindus mukorossi* Gaertn. (NC_025554), and *Xanthoceras* (NC_037448), but are absent in *Aesculus wangii* Hu (NC_035955). They have also been detected in other families of Sapindales, including Meliaceae, Rutaceae, Nitrariaceae, and Simaroubaceae (Lu et al., 2017; Mader et al., 2018; Saina et al., 2018). In the case of *Acer*, this feature appears to be exclusive to species of section *Palmata*.

In the 16 plastomes studied, the i*nfA* gene was found as a pseudogene, with no start codon and the open reading frame disrupted by internal stop codons. In all other Sapindaceae surveyed (listed above), *infA* also appears as a pseudogene suggesting that it became obsolete in the ancestor of this family or perhaps of Sapindales. It has been reported as missing or as a pseudogene, in several genera of Meliaceae (Mader et al., 2018), Simaroubaceae (Saina et al., 2018), Nitrariaceae (Lu et al., 2017), and Anacardiaceae (Wang et al., 2020). The loss of this gene from cpDNA has been documented in many other angiosperm lineages, and there is even indication of its transfer to the nuclear genome in a number of species (Daniell et al., 2016; Millen et al., 2001).

The ribosomal protein genes *rps2* and *rpl22* exhibited considerable variation in length and might be non-functional in several species. In *A. acuminatum*, *A. carpinifolium*, *A. micranthum*, *A. negundo*, *A. nipponicum*, *A. palmatum*, *A. pilosum*, *A. pseudoplatanus*, *A. rubrum*, and *A. tataricum*, *rps2* was 60–80% shorter compared to the other *Acer* species and genera of Sapindales. In the case of *rpl22*, it was 50 and 60% shorter in *A. sterculiaceum* and *A. pentaphyllum*, respectively. The likely loss of function in these species is notable because both genes are considered essential for plant survival (Daniell et al., 2016; Tiller & Bock, 2014). A possible explanation is that they have been transferred to the nucleus, as documented for *rpl22* in Fabaceae and Fagaceae (Jansen et al., 2011). Another possibility is their substitution by a nuclear gene that encodes chloroplast (and mitochondrion) targeted proteins as is the case of the *rps16* gene in some plant lineages (Keller et al., 2017; Ueda et al., 2008). A search for these genes in the nuclear and mitochondrial genomes will reveal insights into their evolutionary fate in *Acer*.

In addition to providing insight into the structure and gene organization, genome comparisons are useful for identifying variable regions suitable for the development of molecular markers. In *Acer*, only a few cpDNA loci (*atpB-rbcL*, *ndhF*, *psbA-trnH*, *psbM-trnD*, *rbcL*, *rpl16*, *trnD-trnT*, *trnL* and *trnL-trnF*) have been employed in phylogenetic (Li, 2011; Li, Yue & Shoup, 2006; Pfosser et al., 2002; Renner et al., 2007; Renner et al., 2008; Tian, Guo & Li, 2002; Zhang, Li & Li, 2010) and phylogeographic studies (Guo et al., 2014; Saeki et al., 2011). The maximum number included in a single dataset is six, but the resulting trees failed to provide adequate resolution and support for many major clades (Renner et al., 2007; Renner et al., 2008). Two additional loci, *matK* and *trnS-trnG*, were explored along with *rbcL* as barcodes to distinguish among 85 *Acer* species, but they also showed low discrimination power even when used in combination (Han et al., 2016). Only one of these commonly used markers, *psbA-trnH*, was identified in this study among the most variable in the genus. We found that 14 other loci (Table 3) exhibited greater variation and are thus potentially more informative than the previously used sequences. This supports the notion that universal markers may not be variable enough for many groups and underscores the importance of identifying specific loci for phylogenetic studies at the genus and family level (Cvetkovic, Hinsinger & Strijk, 2019).

### Codon usage and repeat sequence analysis

The pattern of codon usage in *Acer* plastomes is very similar to that of other members of Sapindales. The three most frequent codons (AUU, AAA, and GAA) and the least frequent (UGC, AGC, and CGC) are shared, for example, with *Ailanthus* (Simaroubaceae), *Nitraria* (Nitrariaceae) and *Toxicodendron* (Anacardiaceae) (Lu et al., 2017; Saina et al., 2018; Wang et al., 2020). The most commonly specified amino acids, leucine and isoleucine, are also the most commonly specified in these and many other plant genera (Li et al., 2018; Silva et al., 2018; Yang et al., 2018).

Codon usage was strongly biased toward codons ending in A or T, i.e., there are more codons ending in A or T (68–69% of all codons), and these are used more often (RSCU>1) than the ones ending in C or G (Table 4). This pattern has been observed in most chloroplast genomes of plants (Morton, 2000; Shimada & Sugiura, 1991), and has been related to the high AT composition bias of the protein-coding genes (Morton, 2000). However, selection for translation efficiency is likely the most important factor driving the codon usage of plastid genes (Suzuki & Morton, 2016).

Repetitive elements are a common feature of plastomes, although the amount and distribution can vary widely among plant groups (Sveinsson & Cronk, 2014); (Wicke et al., 2011). In the *Acer* plastomes analyzed, dispersed repeats of 25 bp and longer were few, ranging between four and eight in most species (Fig. 4). Except for the IRs, all had less than 60 bp in length and were more often located in the non-coding regions of the plastome conforming to the general pattern in angiosperms (Wicke et al., 2011). In the case of SSRs, T and A mononucleotide repeats were more frequent than C/G repeats and than more complex motifs, and were more abundant in non-coding DNA, also conforming to the trend observed in many plant groups (Ebert & Peakall, 2009).

SSRs are among the most variable components of the genome, and constitute an invaluable source of information for population genetic studies, DNA fingerprinting, and plant breeding programs (Nybom, Weising & Rotter, 2014; Wheeler et al., 2014). They have been used to characterize the genetic diversity in *Acer* species of economic and conservation interest (e.g., *Acer campestre* L., *Acer capillipes* Maxim., *Acer mono* Maxim. *Acer opalus* Mill., *A. pseudoplatanus*, *A. saccharum* and *Acer yangbiense* Y. S. Chen & Q. E. Yang). However, most studies have employed nuclear (nSSRs) microsatellite loci (Chybicki, Waldon-Rudzionek & Meyza, 2014; Graignic, Tremblay & Bergeron, 2013; Kikuchi & Shibata, 2008; Liu et al., 2014; Neophytou, Konnert & Fussi, 2019; Pandey et al., 2012; Segarra-Moragues, Gleiser & González-Candelas, 2008; Terui et al., 2006; Zhao, Sun & Yang, 2011), and very few have used cpSSRs (Petit et al., 2003; Saeki et al., 2011; Neophytou, Konnert & Fussi, 2019). Our SSR analysis revealed a large number of these repeats distributed across non-coding and protein-coding regions of the *Acer* plastomes (Table S5) providing an opportunity for the development of new cpSSRs markers for the genus. Because plastids are maternally inherited in *Acer* (Corriveau & Coleman, 1988), cpSSRs can be particularly useful for studies of gene flow through seed dispersal, and for tracing the maternal lineage in space and time (Nybom, Weising & Rotter, 2014; Petit et al., 2003; Provan, Powell & Hollingsworth, 2001).

The locus with the highest number of SSRs was *ycf1*. This gene was also identified among the most variable in *Acer*. It has been reported as among the most variable of the plastome in many plant groups (Jiang, Hinsinger & Strijk, 2016; Kumar et al., 2009; Neubig et al., 2009; Silva et al., 2018; Thomson, Vargas & Dick, 2018), and has even been recommended as a barcode marker (Dong et al., 2015). However, it has been rarely used for phylogenetic inference and barcoding compared to other loci. Future studies should investigate the potential of this gene as a phylogenetic marker in *Acer* and other plant groups.

### Phylogenomic reconstruction

We conducted ML and Bayesian analyses with 22 species of *Acer* using two different partitioning strategies (i.e., a fully partitioned and an unpartitioned dataset) to explore the effect of partitioning on tree inference. We found that both methods produced identical trees with similar support values regardless of the partitioning strategy used.

It is widely accepted that partitioning is important to account for rate heterogeneity and patterns of substitution among sites and that the choice of partitioning scheme can affect the phylogenetic inference (Brown & Lemmon, 2007; Kainer & Lanfear, 2015; Lanfear et al., 2014). A number of studies that have investigated the effect of partitioning have reported improvement in tree topology, branch lengths, and branch support when the data is appropriately partitioned. In contrast, unpartitioned or poorly partitioned analyses may lead to well-supported but incorrect relationships (Brandley, Schmitz & Reeder, 2005; Kainer & Lanfear, 2015; Tao, Mayden & He, 2013; Ward et al., 2010). In our case, partitioning the cp dataset into 93 subsets (based on spacers, genes, introns, exons, and codon position) did not have an effect on the phylogenetic reconstruction. A similar outcome was reported by Fu et al. (2017) using plastid genomes of Cornales and by Dong et al. (2018) with Saxifragales. The results of these studies, including our own, suggest that the effect of partitioning might be less important in large datasets, presumably due to the increase in phylogenetic signal. Kainer & Lanfear (2015) examined the impact of partitioning scheme choice by analyzing 34 datasets, the largest of which had over 25,000 sites. They found that “the longer the alignment, the less the results depend on the partitioning scheme”. With typically more than 130 kb in length, plastid genome data sets appear to be large enough (and to contain enough phylogenetic signal) to converge on the correct tree irrespective of the partitioning scheme used. It would be interesting, nonetheless, to explore further the effect of partitioning on tree inference using a diverse set of complete plastid genome data.

Our phylogenetic analyses confirmed many of the relationships inferred in previous studies using cp markers (Renner et al., 2007; Renner et al., 2008). These include the earliest-diverging position of the clade formed by *A. glabrum* and *A. pseudoplatanus*, the close relationship between *A. nipponicum* and *A. carpinifolium* (sections *Parviflora* and *Indivisa* respectively), the sister relationship of section *Pentaphylla* to the *Trifoliata-Oblonga* clade, and the sister relationship of sections *Macrantha* and *Platanoidea* (Fig. 7). Previously unresolved or unsupported relationships in the cp tree were also clarified in our analysis. For example, section *Lithocarpa*, represented here by *Acer sterculiaceum* Wall. was resolved as sister to the clade comprising members of section *Palmata* with maximum support. Section *Rubra*, *A. negundo,* and *A. pilosum*, whose positions were also unresolved, were placed among the early-diverging lineages of the genus (Fig. 7). The placement of *A. tataricum*, the only species of sect. *Ginnala*, differed from previous studies based on cp data. This species was recovered by Renner et al. (2007) and Renner et al. (2008) as sister to the clade comprising members of section *Platanoidea*, *Macrantha*, *Arguta*, and part of *Negundo*, although with no support. In our study, *A. tataricum* was placed as sister to a major clade formed by species of sections *Platanoidea*, *Macrantha*, *Arguta, Trifoliata*, *Oblonga*, and *Pentaphylla* with maximum support (Fig. 7). Overall, these results represent a significant improvement over previous studies based on cp markers, which have been characterized by poor resolution and statistical support (Harris et al., 2017; Li, Yue & Shoup, 2006; Renner et al., 2007; Renner et al., 2008).

A comparison with the phylogenomic analysis by Li et al. (2019) based on nuclear sequences reveals very different topologies between the plastid and nuclear trees. In the nuclear tree, *Acer* species were grouped into two main lineages, one comprising members of sections *Spicata*, *Palmata*, *Negundo* and *Arguta*, and the other including the remaining sections. Relationships among sections within each of these two clades also differed markedly between the two trees. For example, sect. *Platanoidea* and *Macrantha* were not sister groups in the nuclear tree as was inferred in our cp tree (Fig. 7). Section *Macrantha* was recovered as sister to a clade comprising species of sect. *Ginnala*, *Indivisa*, *Lithocarpa*, *Platanoidea*, *Rubra*, *Acer*, *Trifoliata*, and *Pentaphylla*, whereas monotypic sect. *Glabra* was sister to sect. *Parviflora* (Li et al., 2019).

Unlike nuclear trees, which typically tend to be consistent with taxonomy (as is the case in *Acer*), cp-based trees often correlate with geographic patterns, which can be explained by the occurrence of hybridization and introgression (Albaladejo et al., 2005; McKinnon et al., 1999; Rautenberg et al., 2010). Natural hybridization has been documented in *Acer* (e.g., Liao et al., 2010). However, few studies have employed molecular tools to study its contribution to the evolution of the genus. For example, Grimm, Denk & Hemleben (2007), using nuclear markers, found evidence of ancient hybridization and introgression in *Acer* section *Acer*, a group exhibiting high morphological variability. Also, polyploidization has been suggested to have played a role in the diversification of section *Rubra*, which includes diploid, tetraploid, hexaploid, and octoploid species (Harris et al., 2017), although no study has been conducted to test this hypothesis. Unfortunately, the limited sampling of cp genomes in this study does not allow detecting any apparent geographic pattern, except perhaps for the position of the three North American species (A. *glabrum*, A. *negundo*, and A. *rubrum*) which were placed among the earliest-diverging lineages (Fig. 7).

There are three polyploid species in our study, and their discordant positions in the plastid and nuclear trees (Li et al., 2019) might indicate past hybridization and introgression. *Acer rubrum* is hexaploid and octoploid, whereas *Acer carpinifolium* and *A. pseudoplatanus* are both tetraploids (Contreras & Shearer, 2018). *Acer pseudoplatanus* (section *Acer*) is an autotetraploid (Grimm, Denk & Hemleben, 2007; Pandey et al., 2012) i.e., its polyploidy did not result from hybrid speciation. Yet, its different placement in the plastid and nuclear trees suggests a hybrid origin for this species. In the cp tree, this species was placed as sister to *A. glabrum* (section *Glabra*) in the earliest diverging lineage of *Acer* with maximum support (Fig. 7). It was also recovered as sister to *A. glabrum* in the analyses of Renner et al. (2007) and Renner et al. (2008) and outside the clade comprising most species of section *Acer*. However, in the nuclear tree of Li et al. (2019), *A. pseudoplatanus* was placed in a monophyletic *Acer* section, also with strong support. This incongruence might be explained by chloroplast capture via hybridization between these two lineages (but also by the retention of ancestral polymorphism, i.e., incomplete lineage sorting). Further analyses with an increased sampling within sections (and populations) will likely reveal important clues on the causes of discordance between plastid and nuclear trees, and on the role of hybridization in the diversification of the genus.

The very short internal branches in our trees suggest a rapid differentiation of the main lineages of *Acer* within a short period of time. This is consistent with earlier studies that have analyzed the distribution of the rich fossil record (Boulter et al., 1996; Manchester, 1999; Wolfe & Tanai, 1987), which have suggested a burst of diversification during the second half of the Eocene. Based on nuclear data, Li et al. (2019) estimated that most sections in *Acer* had originated by the late Eocene (33–38 Mya). However, their biogeographic analysis was limited by a narrow sampling (30 taxa) and only two calibration points. Dating analyses incorporating a denser sampling of both plastid and nuclear genomic data are needed to improve our understanding of the diversification and biogeographic history of this diverse genus.

## Conclusions

In this study we assembled, annotated, and compared 16 plastid genomes of maples, each belonging to a different section of the 18 that are currently recognized for the genus. We found that *Acer* plastomes are very similar in structure and gene content. Expanded IRs with two whole copies of the ribosomal *rps16* gene distinguished *Acer palmatum* from the rest of species. Variation in length, possibly accompanied by a loss of function in several species, was detected in the *rps2* and *rpl22* genes. We confirmed that the greater interspecific variation is located in non-coding sequences of the LSC and SSC regions, and identified variable and potentially informative loci that will facilitate the development of markers for species identification, population genetics, and evolutionary studies in the genus.

Our phylogenetic analysis showed that plastome sequences are valuable tools to resolve deep-level relationships that were unclear or poorly supported in earlier studies using cp markers. Future studies with increased taxon sampling are needed to generate a robust plastid tree that, in combination with nuclear data, will contribute to improve our understanding of the evolution of this diverse and economically important group.

##  Supplemental Information

10.7717/peerj.9483/supp-1 Table S1 Species included in this study with GenBank accession numbers Plastomes generated in this study have voucher information and accession numbers in boldface.

10.7717/peerj.9483/supp-2 Table S2 Codon usage for *Acer* plastomes Aa: Amino acid, No: Number of codons, RSCU= Relative synonym codon usage.

10.7717/peerj.9483/supp-3 Table S3 Repeats found in *Acer* plastomes using REPuter Only sequences ≥25 bp were considered. F: forward, P: palindrome, R: reverse; IGS: intergenic spacer, IR: inverted repeat, LSC: large single copy, SSC: small single copy.

10.7717/peerj.9483/supp-4 Table S4 Number of simple sequence repeats (SSRs) found in *Acer* plastomes using MISA-web See Table S1 for full species names.

10.7717/peerj.9483/supp-5 Table S5 Simple sequence repeats (SSRs) found in *Acer* plastomes using MISA-web C: compound repeat, p1: monomeric repeat, p2: dimeric repeat, p3: trimeric repeat, p4: tetrameric repeat; IGS: intergenic spacer, IR: inverted repeat, LSC: large single copy, SSC: small single copy.

10.7717/peerj.9483/supp-6 Table S6 Pairwise percent identity for the 26 species included in the phylogenetic analysis See Table S1 for full species names.

10.7717/peerj.9483/supp-7 Data S1 Annotated sequences of the 16 plastomes generated in this study

10.7717/peerj.9483/supp-8 Data S2 Plastome sequences of section Palmata

## Additional Information and Declarations

### Data Availability

The following information was supplied regarding data availability: The annotated sequences of the 16 plastomes are available at GenBank: MN864496–MN864511 and in Data S1.
