# Supplementary material for: Plastome comparative genomics in maples resolves the infrageneric backbone relationships
Source: PeerJ. 2020 Jul 13;8:e9483. doi: 10.7717/peerj.9483 (PMC7365138; doi:10.7717/peerj.9483)
Supplement: Table S1 — Plastomes generated in this study have voucher information and accession numbers in boldface. [file peerj-08-9483-s001.docx]

**Table S1.** Species included in this study with Genbank accession numbers. Plastomes generated in this study have voucher information and accession numbers in boldface.

| **Species** | **Acer section** | **BGT database number** | **Collection locality and provenance** | **Genbank accession** | **Geographic distribution** |
| --- | --- | --- | --- | --- | --- |
| *Acer acuminatum* Wall. ex D. Don | *Arguta* | M277 | Iturraran Bot. Garden (Tamti, Nepal) | **MN864496** | N Pakistan, N India, Nepal, China |
| *Acer carpinifolium* Siebold & Zucc. | *Indivisa* | M408 | Passadou arboretum (Honshu, Japan) | **MN864497** | Japan |
| *Acer davidii* Franch. | *Macrantha* | --- | --- | NC_030331 | China, Myanmar |
| *Acer glabrum* Torr. | *Glabra* | M266 | Iturraran Bot. Garden (Oregon, USA) | **MN864498** | N America (Alaska, Canada, USA) |
| *Acer griseum* (Franch.) Pax | *Trifoliata* | --- | --- | NC_034346 | China |
| *Acer maximowiczianum* Miq. | *Trifoliata* | M404 | Passadou arboretum (Shikoku, Japan) | **MN864499** | China, Japan |
| *Acer micranthum* Siebold and Zucc. | *Macrantha* | IT19 | Iturraran Bot. Garden (Honshu, Japan) | **MN864500** | Japan |
| *Acer miyabei* subsp. *miaotaiense* (P.C. Tsoong) A.E. Murray | *Platanoidea* | --- | --- | NC_030343 | China |
| *Acer morrisonense* Hayata | *Macrantha* | --- | --- | NC_029371 | Taiwan |
| *Acer negundo* L. | *Negundo* | M383 | Passadou arboretum (Pennsylvania, USA) | **MN864501** | N America (Canada, USA, Mexico) |
| *Acer nipponicum* Hara | *Parviflora* | M263 | Iturraran Bot. Garden (Honshu, Japan) | **MN864502** | Japan |
| *Acer oblongum* Wall. ex DC. | *Oblonga* | M310 | Iturraran Bot. Garden (Hsinchu, Taiwan) | **MN864503** | S and SE Asia (Pakistan, N India, Nepal, Bhutan, Myanmar, Thailand, Laos, Vietnam, China including Taiwan, Japan) |
| *Acer palmatum* Thunb. var. *palmatum* | *Palmata* | M395 | Passadou arboretum (Shikoku, Japan) | **MN864504** | Japan, S Korea |
| *Acer pentaphyllum* Diels | *Pentaphylla* | IT14 | Iturraran Bot. Garden (Sichuan, China) | **MN864505** | China |
| *Acer pilosum* Maxim. | *Pubescentia* | M409 | Passadou arboretum (Shanxi, China) | **MN864506** | China |
| *Acer platanoides* L. | *Platanoidea* | M291 | Iturraran Bot. Garden (Neuler, Germany) | **MN864507** | Europe and SW Asia (Turkey, W Russia, Georgia, Armenia, Azerbaijan, Iran) |
| *Acer pseudoplatanus* L. | *Acer* | M290 | Iturraran Bot. Garden (Altdorf, Germany) | **MN864508** | Europe and SW Asia (W Russia, Georgia, Azerbaijan) |
| *Acer rubrum* L. | *Rubra* | M401 | Passadou arboretum (Maryland, USA) | **MN864509** | N America (Canada, US) |
| *Acer sterculiaceum* Wall. subsp. *sterculiaceum* | *Lithocarpa* | IT15 | Iturraran Bot. Garden (Sichuan, China) | **MN864510** | N India, Nepal, Bhutan, China |
| *Acer tataricum* L. subsp. *ginnala* (Maxim.) Wesm. | *Ginnala* | H120 | Hangzhou Bot. Garden (Chungnam, S Korea) | **MN864511** | E Asia (E Mongolia, China, Korea, E Russia, Japan) |
| *Acer truncatum* Bunge | *Platanoidea* | --- | --- | MG209700 | E Asia (China, Korea, E Russia) |
| *Acer wilsonii* Rehder | *Palmata* | --- | --- | MG012225 | Myanmar, Thailand, Vietnam, China |
| *Dipteronia dyeriana* Henry | --- | --- | --- | NC_031899 | China |
| *Dipteronia sinensis* Oliv. | --- | --- | --- | NC_029338 | China |
| *Litchi chinensis* Sonn. | --- | --- | --- | NC_035238 | SE Asia (Myanmar, Thailand, Laos, Vietnam, China, Malaysia, Indonesia, Philippines) |
| *Spondias mombin* L. | --- | --- | --- | KY828469 | Tropical America |
