# Supplementary material for: Plastome comparative genomics in maples resolves the infrageneric backbone relationships
Source: PeerJ. 2020 Jul 13;8:e9483. doi: 10.7717/peerj.9483 (PMC7365138; doi:10.7717/peerj.9483)
Supplement: Table S2 — Aa: Amino acid, No: Number of codons, RSCU= Relative synonym codon usage. [file peerj-08-9483-s002.doc]

**Table S2.** Codon usage for *Acer* plastomes. Aa: Amino acid, No: Number of codons, RSCU= Relative synonym codon usage.

| ***Acer acuminatum*** (26692 codons) | | | | | | | | | | | | | | | |
| --- | --- | --- | --- | --- | --- | --- | --- | --- | --- | --- | --- | --- | --- | --- | --- |
| **Codon** | **Aa** | **No** | **RSCU** | **Codon** | **Aa** | **No** | **RSCU** | **Codon** | **Aa** | **No** | **RSCU** | **Codon** | **Aa** | **No** | **RSCU** |
| UUU | Phe | 1009 | 1.29 | UCU | Ser | 556 | 1.60 | UAU | Tyr | 774 | 1.61 | UGU | Cys | 212 | 1.44 |
| UUC |  | 559 | 0.71 | UCC |  | 353 | 1.02 | UAC |  | 185 | 0.39 | UGC |  | 83 | 0.56 |
| UUA | Leu | 845 | 1.80 | UCA |  | 437 | 1.26 | UAA* | Stop | 48 | 1.62 | UGA* | Stop | 16 | 0.54 |
| UUG |  | 571 | 1.22 | UCG |  | 196 | 0.57 | UAG* |  | 25 | 0.84 | UGG | Trp | 454 | 1.00 |
| CUU |  | 579 | 1.23 | CCU | Pro | 414 | 1.47 | CAU | His | 495 | 1.49 | CGU | Arg | 318 | 1.18 |
| CUC |  | 218 | 0.46 | CCC |  | 238 | 0.85 | CAC |  | 169 | 0.51 | CGC |  | 131 | 0.49 |
| CUA |  | 396 | 0.84 | CCA |  | 316 | 1.12 | CAA | Gln | 722 | 1.53 | CGA |  | 362 | 1.34 |
| CUG |  | 207 | 0.44 | CCG |  | 156 | 0.56 | CAG |  | 219 | 0.47 | CGG |  | 141 | 0.52 |
| AUU | Ile | 1099 | 1.47 | ACU | Thr | 519 | 1.55 | AAU | Asn | 972 | 1.52 | AGU | Ser | 409 | 1.18 |
| AUC |  | 445 | 0.59 | ACC |  | 261 | 0.78 | AAC |  | 310 | 0.48 | AGC |  | 128 | 0.37 |
| AUA |  | 704 | 0.94 | ACA |  | 401 | 1.20 | AAA | Lys | 1062 | 1.47 | AGA | Arg | 481 | 1.78 |
| AUG | Met | 613 | 1.00 | ACG |  | 155 | 0.46 | AAG |  | 381 | 0.53 | AGG |  | 187 | 0.69 |
| GUU | Val | 528 | 1.44 | GCU | Ala | 613 | 1.74 | GAU | Asp | 843 | 1.57 | GGU | Gly | 591 | 1.29 |
| GUC |  | 190 | 0.52 | GCC |  | 228 | 0.65 | GAC |  | 233 | 0.43 | GGC |  | 186 | 0.41 |
| GUA |  | 544 | 1.49 | GCA |  | 383 | 1.08 | GAA | Glu | 1016 | 1.48 | GGA |  | 721 | 1.57 |
| GUG |  | 203 | 0.55 | GCG |  | 188 | 0.53 | GAG |  | 360 | 0.52 | GGG |  | 334 | 0.73 |
| ***Acer carpinifolium*** (26708 codons) | | | | | | | | | | | | | | | |
| **Codon** | **Aa** | **No** | **RSCU** | **Codon** | **Aa** | **No** | **RSCU** | **Codon** | **Aa** | **No** | **RSCU** | **Codon** | **Aa** | **No** | **RSCU** |
| UUU | Phe | 999 | 1.28 | UCU | Ser | 552 | 1.60 | UAU | Tyr | 788 | 1.63 | UGU | Cys | 214 | 1.45 |
| UUC |  | 561 | 0.72 | UCC |  | 352 | 1.02 | UAC |  | 177 | 0.37 | UGC |  | 82 | 0.55 |
| UUA | Leu | 842 | 1.79 | UCA |  | 435 | 1.26 | UAA* | Stop | 51 | 1.72 | UGA* | Stop | 15 | 0.51 |
| UUG |  | 575 | 1.22 | UCG |  | 203 | 0.59 | UAG* |  | 23 | 0.78 | UGG | Trp | 456 | 1.00 |
| CUU |  | 577 | 1.23 | CCU | Pro | 416 | 1.49 | CAU | His | 496 | 1.49 | CGU | Arg | 320 | 1.18 |
| CUC |  | 223 | 0.47 | CCC |  | 232 | 0.83 | CAC |  | 168 | 0.51 | CGC |  | 128 | 0.47 |
| CUA |  | 389 | 0.83 | CCA |  | 310 | 1.11 | CAA | Gln | 720 | 1.53 | CGA |  | 364 | 1.34 |
| CUG |  | 218 | 0.46 | CCG |  | 159 | 0.57 | CAG |  | 220 | 0.47 | CGG |  | 143 | 0.53 |
| AUU | Ile | 1084 | 1.45 | ACU | Thr | 521 | 1.56 | AAU | Asn | 962 | 1.50 | AGU | Ser | 406 | 1.17 |
| AUC |  | 459 | 0.61 | ACC |  | 259 | 0.77 | AAC |  | 318 | 0.50 | AGC |  | 128 | 0.37 |
| AUA |  | 699 | 0.94 | ACA |  | 401 | 1.20 | AAA | Lys | 1064 | 1.48 | AGA | Arg | 492 | 1.81 |
| AUG | Met | 621 | 1.00 | ACG |  | 159 | 0.47 | AAG |  | 378 | 0.52 | AGG |  | 183 | 0.67 |
| GUU | Val | 534 | 1.45 | GCU | Ala | 608 | 1.72 | GAU | Asp | 850 | 1.58 | GGU | Gly | 591 | 1.29 |
| GUC |  | 190 | 0.52 | GCC |  | 238 | 0.67 | GAC |  | 228 | 0.42 | GGC |  | 186 | 0.41 |
| GUA |  | 546 | 1.48 | GCA |  | 378 | 1.07 | GAA | Glu | 1011 | 1.47 | GGA |  | 726 | 1.59 |
| GUG |  | 202 | 0.55 | GCG |  | 191 | 0.54 | GAG |  | 360 | 0.53 | GGG |  | 327 | 0.71 |
| ***Acer glabrum*** (26865 codons) | | | | | | | | | | | | | | | |
| **Codon** | **Aa** | **No** | **RSCU** | **Codon** | **Aa** | **No** | **RSCU** | **Codon** | **Aa** | **No** | **RSCU** | **Codon** | **Aa** | **No** | **RSCU** |
| UUU | Phe | 1010 | 1.28 | UCU | Ser | 553 | 1.59 | UAU | Tyr | 781 | 1.62 | UGU | Cys | 218 | 1.44 |
| UUC |  | 565 | 0.72 | UCC |  | 355 | 1.02 | UAC |  | 184 | 0.38 | UGC |  | 85 | 0.56 |
| UUA | Leu | 849 | 1.80 | UCA |  | 447 | 1.28 | UAA* | Stop | 51 | 1.72 | UGA* | Stop | 15 | 0.51 |
| UUG |  | 573 | 1.21 | UCG |  | 199 | 0.57 | UAG* |  | 23 | 0.78 | UGG | Trp | 458 | 1.00 |
| CUU |  | 582 | 1.23 | CCU | Pro | 419 | 1.49 | CAU | His | 492 | 1.48 | CGU | Arg | 318 | 1.17 |
| CUC |  | 221 | 0.47 | CCC |  | 230 | 0.82 | CAC |  | 173 | 0.52 | CGC |  | 132 | 0.49 |
| CUA |  | 394 | 0.83 | CCA |  | 317 | 1.13 | CAA | Gln | 728 | 1.53 | CGA |  | 366 | 1.35 |
| CUG |  | 214 | 0.45 | CCG |  | 159 | 0.57 | CAG |  | 223 | 0.47 | CGG |  | 141 | 0.52 |
| AUU | Ile | 1112 | 1.47 | ACU | Thr | 518 | 1.54 | AAU | Asn | 973 | 1.51 | AGU | Ser | 410 | 1.18 |
| AUC |  | 453 | 0.60 | ACC |  | 263 | 0.78 | AAC |  | 314 | 0.49 | AGC |  | 129 | 0.37 |
| AUA |  | 704 | 0.93 | ACA |  | 403 | 1.20 | AAA | Lys | 1068 | 1.48 | AGA | Arg | 486 | 1.79 |
| AUG | Met | 621 | 1.00 | ACG |  | 164 | 0.49 | AAG |  | 380 | 0.52 | AGG |  | 187 | 0.69 |
| GUU | Val | 528 | 1.44 | GCU | Ala | 623 | 1.75 | GAU | Asp | 862 | 1.58 | GGU | Gly | 598 | 1.30 |
| GUC |  | 185 | 0.51 | GCC |  | 230 | 0.65 | GAC |  | 230 | 0.42 | GGC |  | 185 | 0.40 |
| GUA |  | 545 | 1.49 | GCA |  | 385 | 1.08 | GAA | Glu | 1024 | 1.48 | GGA |  | 727 | 1.58 |
| GUG |  | 205 | 0.56 | GCG |  | 185 | 0.52 | GAG |  | 360 | 0.52 | GGG |  | 333 | 0.72 |
| ***Acer maximowiczianum*** (26830 codons) | | | | | | | | | | | | | | | |
| **Codon** | **Aa** | **No** | **RSCU** | **Codon** | **Aa** | **No** | **RSCU** | **Codon** | **Aa** | **No** | **RSCU** | **Codon** | **Aa** | **No** | **RSCU** |
| UUU | Phe | 1004 | 1.28 | UCU | Ser | 551 | 1.58 | UAU | Tyr | 781 | 1.62 | UGU | Cys | 219 | 1.46 |
| UUC |  | 561 | 0.72 | UCC |  | 354 | 1.02 | UAC |  | 185 | 0.38 | UGC |  | 82 | 0.54 |
| UUA | Leu | 846 | 1.80 | UCA |  | 440 | 1.26 | UAA* | Stop | 49 | 1.65 | UGA* | Stop | 16 | 0.54 |
| UUG |  | 570 | 1.21 | UCG |  | 202 | 0.58 | UAG* |  | 24 | 0.81 | UGG | Trp | 456 | 1.00 |
| CUU |  | 586 | 1.25 | CCU | Pro | 415 | 1.47 | CAU | His | 495 | 1.49 | CGU | Arg | 320 | 1.17 |
| CUC |  | 219 | 0.47 | CCC |  | 233 | 0.83 | CAC |  | 169 | 0.51 | CGC |  | 135 | 0.50 |
| CUA |  | 395 | 0.84 | CCA |  | 320 | 1.13 | CAA | Gln | 722 | 1.52 | CGA |  | 363 | 1.33 |
| CUG |  | 208 | 0.44 | CCG |  | 161 | 0.57 | CAG |  | 227 | 0.48 | CGG |  | 142 | 0.52 |
| AUU | Ile | 1102 | 1.46 | ACU | Thr | 513 | 1.53 | AAU | Asn | 970 | 1.51 | AGU | Ser | 412 | 1.18 |
| AUC |  | 457 | 0.61 | ACC |  | 265 | 0.79 | AAC |  | 317 | 0.49 | AGC |  | 129 | 0.37 |
| AUA |  | 700 | 0.93 | ACA |  | 399 | 1.19 | AAA | Lys | 1064 | 1.48 | AGA | Arg | 489 | 1.79 |
| AUG | Met | 621 | 1.00 | ACG |  | 168 | 0.50 | AAG |  | 378 | 0.52 | AGG |  | 187 | 0.69 |
| GUU | Val | 530 | 1.44 | GCU | Ala | 625 | 1.74 | GAU | Asp | 850 | 1.57 | GGU | Gly | 596 | 1.30 |
| GUC |  | 190 | 0.52 | GCC |  | 231 | 0.64 | GAC |  | 232 | 0.43 | GGC |  | 186 | 0.40 |
| GUA |  | 549 | 1.49 | GCA |  | 386 | 1.08 | GAA | Glu | 1024 | 1.48 | GGA |  | 725 | 1.58 |
| GUG |  | 202 | 0.55 | GCG |  | 191 | 0.53 | GAG |  | 360 | 0.52 | GGG |  | 332 | 0.72 |
| ***Acer micranthum*** (26714 codons) | | | | | | | | | | | | | | | |
| **Codon** | **Aa** | **No** | **RSCU** | **Codon** | **Aa** | **No** | **RSCU** | **Codon** | **Aa** | **No** | **RSCU** | **Codon** | **Aa** | **No** | **RSCU** |
| UUU | Phe | 1007 | 1.29 | UCU | Ser | 553 | 1.60 | UAU | Tyr | 771 | 1.61 | UGU | Cys | 213 | 1.44 |
| UUC |  | 558 | 0.71 | UCC |  | 354 | 1.02 | UAC |  | 187 | 0.39 | UGC |  | 83 | 0.56 |
| UUA | Leu | 848 | 1.81 | UCA |  | 435 | 1.25 | UAA* | Stop | 51 | 1.72 | UGA* | Stop | 15 | 0.51 |
| UUG |  | 564 | 1.20 | UCG |  | 198 | 0.57 | UAG* |  | 23 | 0.78 | UGG | Trp | 457 | 1.00 |
| CUU |  | 580 | 1.24 | CCU | Pro | 412 | 1.48 | CAU | His | 500 | 1.50 | CGU | Arg | 319 | 1.18 |
| CUC |  | 214 | 0.46 | CCC |  | 231 | 0.83 | CAC |  | 167 | 0.50 | CGC |  | 129 | 0.48 |
| CUA |  | 397 | 0.85 | CCA |  | 322 | 1.15 | CAA | Gln | 720 | 1.54 | CGA |  | 361 | 1.33 |
| CUG |  | 212 | 0.45 | CCG |  | 152 | 0.54 | CAG |  | 218 | 0.46 | CGG |  | 148 | 0.55 |
| AUU | Ile | 1103 | 1.47 | ACU | Thr | 516 | 1.55 | AAU | Asn | 971 | 1.51 | AGU | Ser | 410 | 1.18 |
| AUC |  | 445 | 0.59 | ACC |  | 264 | 0.79 | AAC |  | 318 | 0.49 | AGC |  | 130 | 0.38 |
| AUA |  | 702 | 0.94 | ACA |  | 399 | 1.20 | AAA | Lys | 1067 | 1.47 | AGA | Arg | 486 | 1.80 |
| AUG | Met | 619 | 1.00 | ACG |  | 156 | 0.47 | AAG |  | 384 | 0.53 | AGG |  | 181 | 0.67 |
| GUU | Val | 532 | 1.45 | GCU | Ala | 608 | 1.72 | GAU | Asp | 842 | 1.57 | GGU | Gly | 586 | 1.28 |
| GUC |  | 185 | 0.50 | GCC |  | 234 | 0.66 | GAC |  | 230 | 0.43 | GGC |  | 193 | 0.42 |
| GUA |  | 546 | 1.49 | GCA |  | 379 | 1.07 | GAA | Glu | 1017 | 1.48 | GGA |  | 729 | 1.59 |
| GUG |  | 205 | 0.56 | GCG |  | 195 | 0.55 | GAG |  | 357 | 0.52 | GGG |  | 326 | 0.71 |
| ***Acer negundo*** (26714 codons) | | | | | | | | | | | | | | | |
| **Codon** | **Aa** | **No** | **RSCU** | **Codon** | **Aa** | **No** | **RSCU** | **Codon** | **Aa** | **No** | **RSCU** | **Codon** | **Aa** | **No** | **RSCU** |
| UUU | Phe | 1011 | 1.29 | UCU | Ser | 553 | 1.60 | UAU | Tyr | 781 | 1.62 | UGU | Cys | 212 | 1.44 |
| UUC |  | 562 | 0.71 | UCC |  | 354 | 1.02 | UAC |  | 182 | 0.38 | UGC |  | 83 | 0.56 |
| UUA | Leu | 837 | 1.78 | UCA |  | 437 | 1.26 | UAA* | Stop | 51 | 1.72 | UGA* | Stop | 15 | 0.51 |
| UUG |  | 571 | 1.22 | UCG |  | 198 | 0.57 | UAG* |  | 23 | 0.78 | UGG | Trp | 458 | 1.00 |
| CUU |  | 578 | 1.23 | CCU | Pro | 411 | 1.47 | CAU | His | 495 | 1.50 | CGU | Arg | 321 | 1.19 |
| CUC |  | 214 | 0.46 | CCC |  | 232 | 0.83 | CAC |  | 167 | 0.50 | CGC |  | 134 | 0.50 |
| CUA |  | 400 | 0.85 | CCA |  | 323 | 1.16 | CAA | Gln | 724 | 1.54 | CGA |  | 360 | 1.33 |
| CUG |  | 215 | 0.46 | CCG |  | 151 | 0.54 | CAG |  | 218 | 0.46 | CGG |  | 143 | 0.53 |
| AUU | Ile | 1102 | 1.47 | ACU | Thr | 520 | 1.56 | AAU | Asn | 974 | 1.51 | AGU | Ser | 407 | 1.18 |
| AUC |  | 445 | 0.59 | ACC |  | 258 | 0.77 | AAC |  | 313 | 0.49 | AGC |  | 128 | 0.37 |
| AUA |  | 701 | 0.94 | ACA |  | 400 | 1.20 | AAA | Lys | 1065 | 1.47 | AGA | Arg | 482 | 1.78 |
| AUG | Met | 619 | 1.00 | ACG |  | 157 | 0.47 | AAG |  | 383 | 0.53 | AGG |  | 181 | 0.67 |
| GUU | Val | 529 | 1.45 | GCU | Ala | 610 | 1.72 | GAU | Asp | 838 | 1.58 | GGU | Gly | 590 | 1.28 |
| GUC |  | 185 | 0.51 | GCC |  | 238 | 0.67 | GAC |  | 226 | 0.42 | GGC |  | 191 | 0.41 |
| GUA |  | 544 | 1.49 | GCA |  | 385 | 1.08 | GAA | Glu | 1015 | 1.48 | GGA |  | 728 | 1.58 |
| GUG |  | 205 | 0.56 | GCG |  | 189 | 0.53 | GAG |  | 359 | 0.52 | GGG |  | 333 | 0.72 |
| ***Acer nipponicum*** (26739 codons) | | | | | | | | | | | | | | | |
| **Codon** | **Aa** | **No** | **RSCU** | **Codon** | **Aa** | **No** | **RSCU** | **Codon** | **Aa** | **No** | **RSCU** | **Codon** | **Aa** | **No** | **RSCU** |
| UUU | Phe | 1009 | 1.29 | UCU | Ser | 556 | 1.60 | UAU | Tyr | 781 | 1.62 | UGU | Cys | 219 | 1.44 |
| UUC |  | 558 | 0.71 | UCC |  | 353 | 1.02 | UAC |  | 182 | 0.38 | UGC |  | 86 | 0.56 |
| UUA | Leu | 848 | 1.80 | UCA |  | 440 | 1.27 | UAA* | Stop | 52 | 1.75 | UGA* | Stop | 15 | 0.51 |
| UUG |  | 568 | 1.21 | UCG |  | 199 | 0.57 | UAG* |  | 22 | 0.74 | UGG | Trp | 452 | 1.00 |
| CUU |  | 582 | 1.24 | CCU | Pro | 414 | 1.48 | CAU | His | 495 | 1.49 | CGU | Arg | 318 | 1.18 |
| CUC |  | 217 | 0.46 | CCC |  | 229 | 0.82 | CAC |  | 168 | 0.51 | CGC |  | 127 | 0.47 |
| CUA |  | 397 | 0.84 | CCA |  | 320 | 1.14 | CAA | Gln | 722 | 1.53 | CGA |  | 363 | 1.34 |
| CUG |  | 209 | 0.44 | CCG |  | 155 | 0.55 | CAG |  | 224 | 0.47 | CGG |  | 140 | 0.52 |
| AUU | Ile | 1100 | 1.46 | ACU | Thr | 521 | 1.56 | AAU | Asn | 967 | 1.51 | AGU | Ser | 406 | 1.17 |
| AUC |  | 447 | 0.60 | ACC |  | 259 | 0.78 | AAC |  | 313 | 0.49 | AGC |  | 128 | 0.37 |
| AUA |  | 706 | 0.94 | ACA |  | 400 | 1.20 | AAA | Lys | 1071 | 1.48 | AGA | Arg | 488 | 1.81 |
| AUG | Met | 617 | 1.00 | ACG |  | 156 | 0.47 | AAG |  | 379 | 0.52 | AGG |  | 185 | 0.68 |
| GUU | Val | 529 | 1.45 | GCU | Ala | 618 | 1.74 | GAU | Asp | 849 | 1.58 | GGU | Gly | 593 | 1.29 |
| GUC |  | 186 | 0.51 | GCC |  | 232 | 0.65 | GAC |  | 226 | 0.42 | GGC |  | 187 | 0.41 |
| GUA |  | 543 | 1.48 | GCA |  | 385 | 1.08 | GAA | Glu | 1018 | 1.48 | GGA |  | 727 | 1.58 |
| GUG |  | 205 | 0.56 | GCG |  | 187 | 0.53 | GAG |  | 361 | 0.52 | GGG |  | 330 | 0.72 |
| ***Acer oblongum*** (26934 codons) | | | | | | | | | | | | | | | |
| **Codon** | **Aa** | **No** | **RSCU** | **Codon** | **Aa** | **No** | **RSCU** | **Codon** | **Aa** | **No** | **RSCU** | **Codon** | **Aa** | **No** | **RSCU** |
| UUU | Phe | 1007 | 1.29 | UCU | Ser | 549 | 1.58 | UAU | Tyr | 783 | 1.62 | UGU | Cys | 217 | 1.45 |
| UUC |  | 560 | 0.71 | UCC |  | 355 | 1.02 | UAC |  | 184 | 0.38 | UGC |  | 82 | 0.55 |
| UUA | Leu | 850 | 1.80 | UCA |  | 436 | 1.25 | UAA* | Stop | 49 | 1.65 | UGA* | Stop | 16 | 0.54 |
| UUG |  | 570 | 1.21 | UCG |  | 204 | 0.59 | UAG* |  | 24 | 0.81 | UGG | Trp | 455 | 1.00 |
| CUU |  | 586 | 1.24 | CCU | Pro | 417 | 1.47 | CAU | His | 495 | 1.49 | CGU | Arg | 323 | 1.18 |
| CUC |  | 221 | 0.47 | CCC |  | 234 | 0.83 | CAC |  | 169 | 0.51 | CGC |  | 132 | 0.48 |
| CUA |  | 393 | 0.83 | CCA |  | 320 | 1.13 | CAA | Gln | 727 | 1.53 | CGA |  | 362 | 1.33 |
| CUG |  | 209 | 0.44 | CCG |  | 161 | 0.57 | CAG |  | 223 | 0.47 | CGG |  | 146 | 0.53 |
| AUU | Ile | 1100 | 1.46 | ACU | Thr | 516 | 1.53 | AAU | Asn | 969 | 1.50 | AGU | Ser | 412 | 1.19 |
| AUC |  | 459 | 0.61 | ACC |  | 264 | 0.78 | AAC |  | 319 | 0.50 | AGC |  | 130 | 0.37 |
| AUA |  | 699 | 0.93 | ACA |  | 399 | 1.19 | AAA | Lys | 1066 | 1.48 | AGA | Arg | 488 | 1.79 |
| AUG | Met | 623 | 1.00 | ACG |  | 167 | 0.50 | AAG |  | 379 | 0.52 | AGG |  | 188 | 0.69 |
| GUU | Val | 532 | 1.45 | GCU | Ala | 622 | 1.74 | GAU | Asp | 846 | 1.57 | GGU | Gly | 593 | 1.29 |
| GUC |  | 191 | 0.52 | GCC |  | 229 | 0.64 | GAC |  | 233 | 0.43 | GGC |  | 186 | 0.41 |
| GUA |  | 546 | 1.48 | GCA |  | 386 | 1.08 | GAA | Glu | 1018 | 1.47 | GGA |  | 727 | 1.59 |
| GUG |  | 202 | 0.55 | GCG |  | 196 | 0.55 | GAG |  | 363 | 0.53 | GGG |  | 327 | 0.71 |
| ***Acer palmatum* var. *palmatum*** (26821 codons) | | | | | | | | | | | | | | | |
| **Codon** | **Aa** | **No** | **RSCU** | **Codon** | **Aa** | **No** | **RSCU** | **Codon** | **Aa** | **No** | **RSCU** | **Codon** | **Aa** | **No** | **RSCU** |
| UUU | Phe | 1011 | 1.29 | UCU | Ser | 548 | 1.58 | UAU | Tyr | 778 | 1.61 | UGU | Cys | 211 | 1.42 |
| UUC |  | 559 | 0.71 | UCC |  | 358 | 1.03 | UAC |  | 186 | 0.39 | UGC |  | 86 | 0.58 |
| UUA | Leu | 844 | 1.80 | UCA |  | 446 | 1.28 | UAA* | Stop | 52 | 1.73 | UGA* | Stop | 15 | 0.50 |
| UUG |  | 570 | 1.21 | UCG |  | 197 | 0.57 | UAG* |  | 23 | 0.77 | UGG | Trp | 460 | 1.00 |
| CUU |  | 588 | 1.25 | CCU | Pro | 420 | 1.50 | CAU | His | 508 | 1.50 | CGU | Arg | 322 | 1.18 |
| CUC |  | 214 | 0.46 | CCC |  | 228 | 0.81 | CAC |  | 168 | 0.50 | CGC |  | 134 | 0.49 |
| CUA |  | 392 | 0.83 | CCA |  | 315 | 1.13 | CAA | Gln | 723 | 1.53 | CGA |  | 364 | 1.34 |
| CUG |  | 212 | 0.45 | CCG |  | 157 | 0.56 | CAG |  | 222 | 0.47 | CGG |  | 145 | 0.53 |
| AUU | Ile | 1101 | 1.46 | ACU | Thr | 523 | 1.55 | AAU | Asn | 974 | 1.51 | AGU | Ser | 411 | 1.18 |
| AUC |  | 449 | 0.60 | ACC |  | 266 | 0.79 | AAC |  | 316 | 0.49 | AGC |  | 124 | 0.36 |
| AUA |  | 709 | 0.94 | ACA |  | 406 | 1.20 | AAA | Lys | 1079 | 1.48 | AGA | Arg | 485 | 1.78 |
| AUG | Met | 616 | 1.00 | ACG |  | 155 | 0.46 | AAG |  | 382 | 0.52 | AGG |  | 181 | 0.67 |
| GUU | Val | 524 | 1.43 | GCU | Ala | 616 | 1.73 | GAU | Asp | 840 | 1.57 | GGU | Gly | 591 | 1.28 |
| GUC |  | 190 | 0.52 | GCC |  | 233 | 0.65 | GAC |  | 228 | 0.43 | GGC |  | 189 | 0.41 |
| GUA |  | 547 | 1.49 | GCA |  | 384 | 1.08 | GAA | Glu | 1018 | 1.47 | GGA |  | 733 | 1.59 |
| GUG |  | 203 | 0.55 | GCG |  | 194 | 0.54 | GAG |  | 370 | 0.53 | GGG |  | 328 | 0.71 |
| ***Acer pentaphyllum*** (26750 codons) | | | | | | | | | | | | | | | |
| **Codon** | **Aa** | **No** | **RSCU** | **Codon** | **Aa** | **No** | **RSCU** | **Codon** | **Aa** | **No** | **RSCU** | **Codon** | **Aa** | **No** | **RSCU** |
| UUU | Phe | 1008 | 1.29 | UCU | Ser | 554 | 1.60 | UAU | Tyr | 773 | 1.61 | UGU | Cys | 214 | 1.45 |
| UUC |  | 557 | 0.71 | UCC |  | 355 | 1.03 | UAC |  | 186 | 0.39 | UGC |  | 81 | 0.55 |
| UUA | Leu | 845 | 1.79 | UCA |  | 437 | 1.26 | UAA* | Stop | 48 | 1.62 | UGA* | Stop | 17 | 0.57 |
| UUG |  | 573 | 1.22 | UCG |  | 196 | 0.57 | UAG* |  | 24 | 0.81 | UGG | Trp | 454 | 1.00 |
| CUU |  | 588 | 1.25 | CCU | Pro | 416 | 1.48 | CAU | His | 495 | 1.50 | CGU | Arg | 319 | 1.17 |
| CUC |  | 221 | 0.47 | CCC |  | 232 | 0.83 | CAC |  | 166 | 0.50 | CGC |  | 136 | 0.50 |
| CUA |  | 392 | 0.83 | CCA |  | 317 | 1.13 | CAA | Gln | 721 | 1.53 | CGA |  | 364 | 1.34 |
| CUG |  | 207 | 0.44 | CCG |  | 156 | 0.56 | CAG |  | 224 | 0.47 | CGG |  | 144 | 0.53 |
| AUU | Ile | 1104 | 1.47 | ACU | Thr | 513 | 1.53 | AAU | Asn | 970 | 1.51 | AGU | Ser | 408 | 1.18 |
| AUC |  | 454 | 0.60 | ACC |  | 261 | 0.78 | AAC |  | 316 | 0.49 | AGC |  | 128 | 0.37 |
| AUA |  | 702 | 0.93 | ACA |  | 401 | 1.20 | AAA | Lys | 1064 | 1.48 | AGA | Arg | 482 | 1.78 |
| AUG | Met | 621 | 1.00 | ACG |  | 167 | 0.50 | AAG |  | 378 | 0.52 | AGG |  | 184 | 0.68 |
| GUU | Val | 532 | 1.45 | GCU | Ala | 619 | 1.74 | GAU | Asp | 847 | 1.57 | GGU | Gly | 592 | 1.29 |
| GUC |  | 188 | 0.51 | GCC |  | 229 | 0.64 | GAC |  | 230 | 0.43 | GGC |  | 186 | 0.41 |
| GUA |  | 539 | 1.47 | GCA |  | 388 | 1.09 | GAA | Glu | 1020 | 1.48 | GGA |  | 723 | 1.58 |
| GUG |  | 204 | 0.56 | GCG |  | 190 | 0.53 | GAG |  | 360 | 0.52 | GGG |  | 330 | 0.72 |
| ***Acer pilosum*** (26699 codons) | | | | | | | | | | | | | | | |
| **Codon** | **Aa** | **No** | **RSCU** | **Codon** | **Aa** | **No** | **RSCU** | **Codon** | **Aa** | **No** | **RSCU** | **Codon** | **Aa** | **No** | **RSCU** |
| UUU | Phe | 1002 | 1.28 | UCU | Ser | 556 | 1.61 | UAU | Tyr | 778 | 1.62 | UGU | Cys | 215 | 1.45 |
| UUC |  | 564 | 0.72 | UCC |  | 348 | 1.00 | UAC |  | 183 | 0.38 | UGC |  | 81 | 0.55 |
| UUA | Leu | 835 | 1.78 | UCA |  | 440 | 1.27 | UAA* | Stop | 50 | 1.69 | UGA* | Stop | 15 | 0.51 |
| UUG |  | 576 | 1.23 | UCG |  | 199 | 0.57 | UAG* |  | 24 | 0.81 | UGG | Trp | 453 | 1.00 |
| CUU |  | 585 | 1.25 | CCU | Pro | 409 | 1.47 | CAU | His | 497 | 1.49 | CGU | Arg | 317 | 1.17 |
| CUC |  | 213 | 0.45 | CCC |  | 232 | 0.83 | CAC |  | 168 | 0.51 | CGC |  | 130 | 0.48 |
| CUA |  | 394 | 0.84 | CCA |  | 316 | 1.13 | CAA | Gln | 722 | 1.53 | CGA |  | 365 | 1.35 |
| CUG |  | 212 | 0.45 | CCG |  | 159 | 0.57 | CAG |  | 222 | 0.47 | CGG |  | 143 | 0.53 |
| AUU | Ile | 1097 | 1.46 | ACU | Thr | 521 | 1.56 | AAU | Asn | 975 | 1.52 | AGU | Ser | 404 | 1.17 |
| AUC |  | 452 | 0.60 | ACC |  | 263 | 0.79 | AAC |  | 307 | 0.48 | AGC |  | 131 | 0.38 |
| AUA |  | 705 | 0.94 | ACA |  | 396 | 1.18 | AAA | Lys | 1062 | 1.48 | AGA | Arg | 481 | 1.78 |
| AUG | Met | 615 | 1.00 | ACG |  | 160 | 0.48 | AAG |  | 376 | 0.52 | AGG |  | 185 | 0.68 |
| GUU | Val | 526 | 1.44 | GCU | Ala | 616 | 1.74 | GAU | Asp | 844 | 1.58 | GGU | Gly | 598 | 1.30 |
| GUC |  | 187 | 0.51 | GCC |  | 228 | 0.64 | GAC |  | 227 | 0.42 | GGC |  | 185 | 0.40 |
| GUA |  | 545 | 1.50 | GCA |  | 383 | 1.08 | GAA | Glu | 1020 | 1.48 | GGA |  | 729 | 1.58 |
| GUG |  | 200 | 0.55 | GCG |  | 192 | 0.54 | GAG |  | 356 | 0.52 | GGG |  | 330 | 0.72 |
| ***Acer platanoides*** (26846 codons) | | | | | | | | | | | | | | | |
| **Codon** | **Aa** | **No** | **RSCU** | **Codon** | **Aa** | **No** | **RSCU** | **Codon** | **Aa** | **No** | **RSCU** | **Codon** | **Aa** | **No** | **RSCU** |
| UUU | Phe | 1008 | 1.29 | UCU | Ser | 551 | 1.59 | UAU | Tyr | 785 | 1.62 | UGU | Cys | 224 | 1.47 |
| UUC |  | 556 | 0.71 | UCC |  | 354 | 1.02 | UAC |  | 186 | 0.38 | UGC |  | 81 | 0.53 |
| UUA | Leu | 853 | 1.81 | UCA |  | 443 | 1.28 | UAA* | Stop | 50 | 1.69 | UGA* | Stop | 16 | 0.54 |
| UUG |  | 569 | 1.21 | UCG |  | 198 | 0.57 | UAG* |  | 23 | 0.78 | UGG | Trp | 460 | 1.00 |
| CUU |  | 587 | 1.24 | CCU | Pro | 421 | 1.49 | CAU | His | 497 | 1.50 | CGU | Arg | 317 | 1.17 |
| CUC |  | 217 | 0.46 | CCC |  | 227 | 0.81 | CAC |  | 166 | 0.50 | CGC |  | 133 | 0.49 |
| CUA |  | 395 | 0.84 | CCA |  | 322 | 1.14 | CAA | Gln | 726 | 1.53 | CGA |  | 367 | 1.35 |
| CUG |  | 211 | 0.45 | CCG |  | 157 | 0.56 | CAG |  | 225 | 0.47 | CGG |  | 139 | 0.51 |
| AUU | Ile | 1104 | 1.46 | ACU | Thr | 517 | 1.54 | AAU | Asn | 977 | 1.51 | AGU | Ser | 403 | 1.16 |
| AUC |  | 460 | 0.61 | ACC |  | 264 | 0.78 | AAC |  | 314 | 0.49 | AGC |  | 131 | 0.38 |
| AUA |  | 705 | 0.93 | ACA |  | 404 | 1.20 | AAA | Lys | 1070 | 1.47 | AGA | Arg | 488 | 1.80 |
| AUG | Met | 621 | 1.00 | ACG |  | 162 | 0.48 | AAG |  | 382 | 0.53 | AGG |  | 186 | 0.68 |
| GUU | Val | 529 | 1.45 | GCU | Ala | 620 | 1.74 | GAU | Asp | 853 | 1.58 | GGU | Gly | 592 | 1.29 |
| GUC |  | 186 | 0.51 | GCC |  | 234 | 0.66 | GAC |  | 230 | 0.42 | GGC |  | 187 | 0.41 |
| GUA |  | 545 | 1.49 | GCA |  | 381 | 1.07 | GAA | Glu | 1019 | 1.48 | GGA |  | 720 | 1.57 |
| GUG |  | 202 | 0.55 | GCG |  | 194 | 0.54 | GAG |  | 362 | 0.52 | GGG |  | 340 | 0.74 |
| ***Acer pseudoplatanus*** (26678 codons) | | | | | | | | | | | | | | | |
| **Codon** | **Aa** | **No** | **RSCU** | **Codon** | **Aa** | **No** | **RSCU** | **Codon** | **Aa** | **No** | **RSCU** | **Codon** | **Aa** | **No** | **RSCU** |
| UUU | Phe | 1010 | 1.29 | UCU | Ser | 551 | 1.59 | UAU | Tyr | 782 | 1.62 | UGU | Cys | 214 | 1.45 |
| UUC |  | 557 | 0.71 | UCC |  | 350 | 1.01 | UAC |  | 184 | 0.38 | UGC |  | 82 | 0.55 |
| UUA | Leu | 841 | 1.80 | UCA |  | 442 | 1.28 | UAA* | Stop | 50 | 1.69 | UGA* | Stop | 15 | 0.51 |
| UUG |  | 563 | 1.20 | UCG |  | 197 | 0.57 | UAG* |  | 24 | 0.81 | UGG | Trp | 457 | 1.00 |
| CUU |  | 576 | 1.23 | CCU | Pro | 418 | 1.50 | CAU | His | 491 | 1.50 | CGU | Arg | 319 | 1.18 |
| CUC |  | 219 | 0.47 | CCC |  | 231 | 0.83 | CAC |  | 164 | 0.50 | CGC |  | 131 | 0.49 |
| CUA |  | 397 | 0.85 | CCA |  | 314 | 1.12 | CAA | Gln | 723 | 1.53 | CGA |  | 364 | 1.35 |
| CUG |  | 214 | 0.46 | CCG |  | 154 | 0.55 | CAG |  | 225 | 0.47 | CGG |  | 141 | 0.52 |
| AUU | Ile | 1100 | 1.47 | ACU | Thr | 514 | 1.54 | AAU | Asn | 958 | 1.51 | AGU | Ser | 406 | 1.17 |
| AUC |  | 448 | 0.60 | ACC |  | 269 | 0.81 | AAC |  | 314 | 0.49 | AGC |  | 131 | 0.38 |
| AUA |  | 702 | 0.94 | ACA |  | 397 | 1.19 | AAA | Lys | 1061 | 1.48 | AGA | Arg | 481 | 1.78 |
| AUG | Met | 611 | 1.00 | ACG |  | 155 | 0.46 | AAG |  | 375 | 0.52 | AGG |  | 184 | 0.68 |
| GUU | Val | 531 | 1.45 | GCU | Ala | 612 | 1.72 | GAU | Asp | 845 | 1.58 | GGU | Gly | 595 | 1.29 |
| GUC |  | 189 | 0.52 | GCC |  | 233 | 0.66 | GAC |  | 228 | 0.42 | GGC |  | 187 | 0.41 |
| GUA |  | 543 | 1.48 | GCA |  | 388 | 1.09 | GAA | Glu | 1019 | 1.48 | GGA |  | 724 | 1.58 |
| GUG |  | 203 | 0.55 | GCG |  | 187 | 0.53 | GAG |  | 356 | 0.52 | GGG |  | 332 | 0.72 |
| ***Acer rubrum*** (26717 codons) | | | | | | | | | | | | | | | |
| **Codon** | **Aa** | **No** | **RSCU** | **Codon** | **Aa** | **No** | **RSCU** | **Codon** | **Aa** | **No** | **RSCU** | **Codon** | **Aa** | **No** | **RSCU** |
| UUU | Phe | 1010 | 1.29 | UCU | Ser | 551 | 1.59 | UAU | Tyr | 780 | 1.62 | UGU | Cys | 214 | 1.45 |
| UUC |  | 561 | 0.71 | UCC |  | 355 | 1.03 | UAC |  | 182 | 0.38 | UGC |  | 82 | 0.55 |
| UUA | Leu | 845 | 1.80 | UCA |  | 433 | 1.25 | UAA* | Stop | 51 | 1.72 | UGA* | Stop | 14 | 0.47 |
| UUG |  | 569 | 1.21 | UCG |  | 203 | 0.59 | UAG* |  | 24 | 0.81 | UGG | Trp | 456 | 1.00 |
| CUU |  | 578 | 1.23 | CCU | Pro | 418 | 1.49 | CAU | His | 499 | 1.50 | CGU | Arg | 319 | 1.18 |
| CUC |  | 218 | 0.46 | CCC |  | 229 | 0.82 | CAC |  | 166 | 0.50 | CGC |  | 128 | 0.47 |
| CUA |  | 396 | 0.84 | CCA |  | 318 | 1.13 | CAA | Gln | 720 | 1.53 | CGA |  | 362 | 1.34 |
| CUG |  | 210 | 0.45 | CCG |  | 157 | 0.56 | CAG |  | 221 | 0.47 | CGG |  | 142 | 0.53 |
| AUU | Ile | 1100 | 1.46 | ACU | Thr | 520 | 1.55 | AAU | Asn | 967 | 1.51 | AGU | Ser | 403 | 1.17 |
| AUC |  | 449 | 0.60 | ACC |  | 261 | 0.78 | AAC |  | 313 | 0.49 | AGC |  | 129 | 0.37 |
| AUA |  | 705 | 0.94 | ACA |  | 399 | 1.19 | AAA | Lys | 1070 | 1.48 | AGA | Arg | 482 | 1.79 |
| AUG | Met | 617 | 1.00 | ACG |  | 159 | 0.47 | AAG |  | 376 | 0.52 | AGG |  | 186 | 0.69 |
| GUU | Val | 532 | 1.46 | GCU | Ala | 610 | 1.72 | GAU | Asp | 844 | 1.57 | GGU | Gly | 595 | 1.29 |
| GUC |  | 185 | 0.51 | GCC |  | 231 | 0.65 | GAC |  | 229 | 0.43 | GGC |  | 187 | 0.41 |
| GUA |  | 539 | 1.47 | GCA |  | 388 | 1.09 | GAA | Glu | 1021 | 1.48 | GGA |  | 724 | 1.57 |
| GUG |  | 206 | 0.56 | GCG |  | 189 | 0.53 | GAG |  | 357 | 0.52 | GGG |  | 333 | 0.72 |
| ***Acer sterculiaceum* subsp. *sterculiaceum*** (26770 codons) | | | | | | | | | | | | | | | |
| **Codon** | **Aa** | **No** | **RSCU** | **Codon** | **Aa** | **No** | **RSCU** | **Codon** | **Aa** | **No** | **RSCU** | **Codon** | **Aa** | **No** | **RSCU** |
| UUU | Phe | 1012 | 1.29 | UCU | Ser | 550 | 1.59 | UAU | Tyr | 777 | 1.62 | UGU | Cys | 215 | 1.44 |
| UUC |  | 560 | 0.71 | UCC |  | 353 | 1.02 | UAC |  | 182 | 0.38 | UGC |  | 84 | 0.56 |
| UUA | Leu | 848 | 1.80 | UCA |  | 438 | 1.27 | UAA* | Stop | 51 | 1.72 | UGA* | Stop | 15 | 0.51 |
| UUG |  | 565 | 1.20 | UCG |  | 206 | 0.60 | UAG* |  | 23 | 0.78 | UGG | Trp | 460 | 1.00 |
| CUU |  | 587 | 1.25 | CCU | Pro | 412 | 1.46 | CAU | His | 495 | 1.50 | CGU | Arg | 319 | 1.16 |
| CUC |  | 223 | 0.47 | CCC |  | 239 | 0.85 | CAC |  | 164 | 0.50 | CGC |  | 129 | 0.47 |
| CUA |  | 392 | 0.83 | CCA |  | 312 | 1.11 | CAA | Gln | 716 | 1.52 | CGA |  | 376 | 1.37 |
| CUG |  | 209 | 0.44 | CCG |  | 164 | 0.58 | CAG |  | 224 | 0.48 | CGG |  | 141 | 0.51 |
| AUU | Ile | 1105 | 1.47 | ACU | Thr | 513 | 1.52 | AAU | Asn | 969 | 1.51 | AGU | Ser | 398 | 1.15 |
| AUC |  | 458 | 0.61 | ACC |  | 263 | 0.78 | AAC |  | 316 | 0.49 | AGC |  | 128 | 0.37 |
| AUA |  | 696 | 0.92 | ACA |  | 406 | 1.20 | AAA | Lys | 1064 | 1.48 | AGA | Arg | 487 | 1.78 |
| AUG | Met | 621 | 1.00 | ACG |  | 167 | 0.50 | AAG |  | 374 | 0.52 | AGG |  | 191 | 0.70 |
| GUU | Val | 528 | 1.45 | GCU | Ala | 616 | 1.74 | GAU | Asp | 856 | 1.58 | GGU | Gly | 592 | 1.29 |
| GUC |  | 186 | 0.51 | GCC |  | 230 | 0.65 | GAC |  | 228 | 0.42 | GGC |  | 191 | 0.42 |
| GUA |  | 545 | 1.50 | GCA |  | 383 | 1.08 | GAA | Glu | 1013 | 1.47 | GGA |  | 721 | 1.57 |
| GUG |  | 199 | 0.55 | GCG |  | 190 | 0.54 | GAG |  | 363 | 0.53 | GGG |  | 332 | 0.72 |
| ***Acer tataricum* subsp. *ginnala*** (26750 codons) | | | | | | | | | | | | | | | |
| **Codon** | **Aa** | **No** | **RSCU** | **Codon** | **Aa** | **No** | **RSCU** | **Codon** | **Aa** | **No** | **RSCU** | **Codon** | **Aa** | **No** | **RSCU** |
| UUU | Phe | 1011 | 1.29 | UCU | Ser | 554 | 1.59 | UAU | Tyr | 777 | 1.62 | UGU | Cys | 218 | 1.45 |
| UUC |  | 561 | 0.71 | UCC |  | 358 | 1.03 | UAC |  | 184 | 0.38 | UGC |  | 83 | 0.55 |
| UUA | Leu | 845 | 1.80 | UCA |  | 440 | 1.26 | UAA* | Stop | 52 | 1.75 | UGA* | Stop | 14 | 0.47 |
| UUG |  | 572 | 1.22 | UCG |  | 205 | 0.59 | UAG* |  | 23 | 0.78 | UGG | Trp | 455 | 1.00 |
| CUU |  | 586 | 1.25 | CCU | Pro | 414 | 1.49 | CAU | His | 496 | 1.49 | CGU | Arg | 317 | 1.17 |
| CUC |  | 215 | 0.46 | CCC |  | 235 | 0.84 | CAC |  | 168 | 0.51 | CGC |  | 131 | 0.48 |
| CUA |  | 394 | 0.84 | CCA |  | 311 | 1.12 | CAA | Gln | 726 | 1.53 | CGA |  | 369 | 1.36 |
| CUG |  | 207 | 0.44 | CCG |  | 154 | 0.55 | CAG |  | 222 | 0.47 | CGG |  | 142 | 0.53 |
| AUU | Ile | 1099 | 1.47 | ACU | Thr | 521 | 1.55 | AAU | Asn | 963 | 1.51 | AGU | Ser | 407 | 1.17 |
| AUC |  | 452 | 0.60 | ACC |  | 268 | 0.80 | AAC |  | 314 | 0.49 | AGC |  | 129 | 0.37 |
| AUA |  | 699 | 0.93 | ACA |  | 398 | 1.19 | AAA | Lys | 1065 | 1.47 | AGA | Arg | 483 | 1.79 |
| AUG | Met | 619 | 1.00 | ACG |  | 155 | 0.46 | AAG |  | 380 | 0.53 | AGG |  | 180 | 0.67 |
| GUU | Val | 529 | 1.45 | GCU | Ala | 618 | 1.74 | GAU | Asp | 849 | 1.58 | GGU | Gly | 586 | 1.28 |
| GUC |  | 187 | 0.51 | GCC |  | 232 | 0.65 | GAC |  | 228 | 0.42 | GGC |  | 193 | 0.42 |
| GUA |  | 543 | 1.48 | GCA |  | 381 | 1.07 | GAA | Glu | 1022 | 1.48 | GGA |  | 726 | 1.58 |
| GUG |  | 205 | 0.56 | GCG |  | 191 | 0.54 | GAG |  | 360 | 0.52 | GGG |  | 329 | 0.72 |
