# Supplementary material for: Plastome comparative genomics in maples resolves the infrageneric backbone relationships
Source: PeerJ. 2020 Jul 13;8:e9483. doi: 10.7717/peerj.9483 (PMC7365138; doi:10.7717/peerj.9483)
Supplement: Table S3 — Only sequences ≥25 bp were considered. F: forward, P: palindrome, R: reverse; IGS: intergenic spacer, IR: inverted repeat, LSC: large single copy, SSC: small single copy. [file peerj-08-9483-s003.docx]

**Table S3.** Repeats found in *Acer* plastomes using REPuter. Only sequences ≥ 25 bp were considered. F: forward, P: palindrome, R: reverse; IGS: intergenic spacer, IR: inverted repeat, LSC: large single copy, SSC: small single copy.

| **Repeat 1 (start location)** | | **Repeat 2 (start location)** | **Size (bp)** | **Type** | **region** |
| --- | --- | --- | --- | --- | --- |
| ***Acer acuminatum*** | | | | | |
| 1 | 85,358 (*rps19*) | 129,476 (*ycf1*) | 26,072 | P | LSC; SSC (IRs) |
| 2 | 70,908 (IGS *rps12* – *clpP*) | 70,937 (IGS *rps12 – clpP*) | 58 | F | LSC |
| 3 | 39,632 (*psaB*) | 41,856 (*psaA*) | 41 | F | LSC |
| 4 | 52,616 (IGS *ndhC* – *trnV-UAC*) | 52,649 (IGS *ndhC – trnV-UAC*) | 34 | F | LSC |
| 5 | 8,692 (IGS *psbI* – *trnS-GCU*) | 46,396 (*trnS-GGA*) | 30 | P | LSC |
| 6 | 70,908 (IGS *rps12 – clpP*) | 70,966 (IGS *rps12 – clpP*) | 29 | F | LSC |
| ***Acer carpinifolium*** | | | | | |
| 1 | 85,448 (*rps19*) | 129,192 (*ycf1*) | 26,020 | P | LSC; SSC (IRs) |
| 2 | 16,899 (IGS *rps2 – rpoC2*) | 16,915 (IGS *rps2 – rpoC2*) | 42 | F | LSC |
| 3 | 40,066 (*psaB*) | 42,290 (*psaA*) | 41 | F | LSC |
| 4 | 8,782 (IGS *psbI* – *trnS-GCU*) | 46,371 (*trnS-GGA*) | 30 | P | LSC |
| 5 | 16,899 (IGS *rps2 – rpoC2*) | 16,931 (IGS *rps2 – rpoC2*) | 26 | F | LSC |
| 6 | 114,546 (IGS *rpl32*  – *trnL-UAG*) | 114,546 (IGS *rpl32*  – *trnL-UAG*) | 26 | R | SSC |
| ***Acer glabrum*** | | | | | |
| 1 | 86,034 (*rps19*) | 130,309 (*ycf1*) | 26,064 | P | LSC; SSC (IRs) |
| 2 | 76,061 (IGS *psbT – psbN*) | 76,061 (IGS *psbT – psbN*) | 44 | P | LSC |
| 3 | 40,247 (*psaB*) | 42,471 (*psaA*) | 41 | F | LSC |
| 4 | 8,878 (IGS *psbI* – *trnS-GCU*) | 47,029 (*trnS-GGA*) | 30 | P | LSC |
| 5 | 109,595 (IGS *rrn5 – trnR-ACG*) | 115,368 (IGS *rpl32*  – *trnL-UAG*) | 28 | F | IRb; SSC |
| 6 | 115,368 (IGS *rpl32*  – *trnL-UAG*) | 132,784 (IGS *trnR-ACG – rrn5*) | 28 | P | SSC; IRa |
| 7 | 23,729 (*rpoC1*) | 23,767 (*rpoC1*) | 26 | F | LSC |
| 8 | 114,372 (IGS *ndhF – rpl32*) | 114,394 (IGS *ndhF – rpl32*) | 26 | F | SSC |
| ***Acer maximowiczianum*** | | | | | |
| 1 | 85,865 (IGS *rps19 – rpl2*) | 130,047 (*ycf1*) | 26,035 | P | LSC; SSC (IRs) |
| 2 | 114,688 (IGS *ndhF – rpl32*) | 114,735 (IGS *ndhF – rpl32*) | 49 | F | SSC |
| 3 | 40,061 (*psaB*) | 42,285 (*psaA*) | 41 | F | LSC |
| 4 | 8,757 (IGS *psbI* – *trnS-GCU*) | 46,794 (*trnS-GGA*) | 30 | P | LSC |
| 5 | 46,853 (IGS *trnS-GGA – rps4*) | 46,879 (IGS *trnS-GGA – rps4*) | 30 | F | LSC |
| ***Acer micranthum*** | | | | | |
| 1 | 86,147 (*rps19*) | 130,337 (*ycf1*) | 26,062 | P | LSC; SSC (IRs) |
| 2 | 40,148 (*psaB*) | 42,372 (*psaA*) | 41 | F | LSC |
| 3 | 29,536 (IGS *petN – psbM*) | 29,536 (IGS *petN – psbM*) | 32 | P | LSC |
| 4 | 8,772 (IGS *psbI – trnS-GCU*) | 46,925 (*trnS-GGA*) | 30 | P | LSC |
| 5 | 59,058 (IGS *rbcL – accD*) | 59,085 (IGS *rbcL – accD*) | 27 | F | LSC |
| 6 | 115,705 (IGS *rpI32 – trnL-UAG*) | 115,705 (IGS *rpI32 – trnL-UAG*) | 26 | R | SSC |
| ***Acer negundo*** | | | | | |
| 1 | 85,678 (*rps19*) | 129,854 (*ycf1*) | 26,084 | P | LSC; SSC (IRs) |
| 2 | 31,608 (IGS *trnE-UUC – trnT-GGU*) | 31,652 (IGS *trnE-UUC – trnT-GGU*) | 45 | F | LSC |
| 3 | 75,745 (IGS *psbT – psbN*) | 75,745 (IGS *psbT – psbN*) | 44 | P | LSC |
| 4 | 40,011 (*psaB*) | 42,235 (*psaA*) | 41 | F | LSC |
| 5 | 8,819 (IGS *psbI – trnS-GCU*) | 46,778 (*trnS-GGA*) | 30 | P | LSC |
| 6 | 66,084 (IGS *psbJ – psbL*) | 66,113 (IGS *psbJ – psbL*) | 30 | F | LSC |
| ***Acer nipponicum*** | | | | | |
| 1 | 85,823 (*rps19*) | 130,140 (*ycf1*) | 26,085 | P | LSC; SSC (IRs) |
| 2 | 10,658 (IGS *trnR-UCU – atpA*) | 10,674 (IGS *trnR-UCU – atpA*) | 46 | F | LSC |
| 3 | 114,359 (IGS *ndhF – rpl32*) | 114,380 (IGS *ndhF – rpl32*) | 43 | F | SSC |
| 4 | 39,971 (*psaB*) | 42,195 (*psaA*) | 41 | F | LSC |
| 5 | 8,571 (IGS *psbI – trnS-GCU*) | 46,769 (*trnS-GGA*) | 30 | P | LSC |
| 6 | 10,658 (IGS *trnR-UCU – atpA*) | 10,690 (IGS *trnR-UCU – atpA*) | 30 | F | LSC |
| 7 | 53,550 (IGS *ndhC – trnV-UAC*) | 53,550 (IGS *ndhC – trnV-UAC*) | 26 | R | LSC |
| ***Acer oblongum*** | | | | | |
| 1 | 85,665 (*rps19*) | 129,586 (*ycf1*) | 26,100 | P | LSC; SSC (IRs) |
| 2 | 39,864 (*psaB*) | 42,088 (*psaA*) | 41 | F | LSC |
| 3 | 8,693 (IGS *psbI – trnS-GCU*) | 46,616 (*trnS-GGA*) | 30 | P | LSC |
| 4 | 10,705 (IGS *trnR-UCU* *– atpA*) | 10,728 (IGS *trnR-UCU* *– atpA*) | 26 | F | LSC |
| ***Acer palmatum* var*. palmatum*** | | | | | |
| 1 | 85,342 (*rpl22*) | 130,266 (*ycf1*) | 26,757 | P | LSC; SSC (IRs) |
| 2 | 116,222 (IGS *rpI32 – trnL-UAG*) | 116,245 (IGS *rpI32 – trnL-UAG*) | 46 | F | SSC |
| 3 | 40,093 (*psaB*) | 42,317 (*psaA*) | 41 | F | LSC |
| 4 | 8,811 (IGS *psbI – trnS-GCU*) | 46,832 (*trnS-GGA*) | 30 | P | LSC |
| 5 | 6,581 (IGS *rps16 – trnQ-UUG*) | 6,607 (IGS *rps16 – trnQ-UUG*) | 28 | F | LSC |
| 6 | 115,594 (IGS *rpI32 – trnL-UAG*) | 115,594 (IGS *rpI32 – trnL-UAG*) | 27 | R | SSC |
| ***Acer pentaphyllum*** | | | | | |
| 1 | 85,938 (*rps19*) | 130,153 (*ycf1*) | 26,067 | P | LSC; SSC (IRs) |
| 2 | 75,840 (IGS *psbT – psbN*) | 75,860 (IGS *psbT – psbN*) | 43 | F | LSC |
| 3 | 40,092 (*psaB*) | 42,316 (*psaA*) | 41 | F | LSC |
| 4 | 23,613 (*rpoC1*) | 23,648 (*rpoC1*) | 31 | F | LSC |
| 5 | 8,816 (IGS *psbI – trnS-GCU*) | 46,834 (*trnS-GGA*) | 30 | P | LSC |
| 6 | 53,591 (IGS *ndhC – trnV-UAC*) | 53,591 (IGS *ndhC – trnV-UAC*) | 26 | R | LSC |
| 7 | 53,597 (IGS *ndhC – trnV-UAC*) | 53,597 (IGS *ndhC – trnV-UAC*) | 25 | R | LSC |
| ***Acer pilosum*** | | | | | |
| 1 | 85,313 (*rps19*) | 129,519 (*ycf1*) | 26,067 | P | LSC; SSC (IRs) |
| 2 | 75,282 (IGS *psbT – psbN*) | 75,282 (IGS *psbT – psbN*) | 44 | P | LSC |
| 3 | 39,675 (*psaB*) | 41,899 (*psaA*) | 41 | F | LSC |
| 4 | 8,426 (IGS *psbI – trnS-GCU*) | 46,429 (*trnS-GGA*) | 30 | P | LSC |
| 5 | 1,638 (IGS *psbA – trnK-UUU*) | 1,638 (IGS *psbA – trnK-UUU*) | 28 | R | LSC |
| ***Acer platanoides*** | | | | | |
| 1 | 86,098 (*rps19*) | 130,295 (*ycf1*) | 26,090 | P | LSC; SSC (IRs) |
| 2 | 119,281 (IGS *ndhD – psaC*) | 119,281 (IGS *ndhD – psaC*) | 38 | P | SSC |
| 3 | 4,850 (IGS *trnK-UUU* – *rps16*) | 4,850 (IGS *trnK-UUU* – *rps16*) | 30 | R | LSC |
| 4 | 8,747 (IGS *psbI – trnS-GCU*) | 46,885 (*trnS-GGA*) | 30 | P | LSC |
| 5 | 4,851 (IGS *trnK-UUU* – *rps16*) | 4,851 (IGS *trnK-UUU* – *rps16*) | 27 | R | LSC |
| 6 | 4,851 (IGS *trnK-UUU* – *rps16*) | 4,852 (IGS *trnK-UUU* – *rps16*) | 27 | F | LSC |
| 7 | 4,852 (IGS *trnK-UUU* – *rps16*) | 4,852 (IGS *trnK-UUU* – *rps16*) | 27 | R | LSC |
| 8 | 4,851 (IGS *trnK-UUU* – *rps16*) | 4,851 (IGS *trnK-UUU* – *rps16*) | 26 | R | LSC |
| 9 | 4,851 (IGS *trnK-UUU* – *rps16*) | 4,853 (IGS *trnK-UUU* – *rps16*) | 26 | F | LSC |
| 10 | 4,853 (IGS *trnK-UUU* – *rps16*) | 4,853 (IGS *trnK-UUU* – *rps16*) | 26 | R | LSC |
| 11 | 8,644 (IGS *psbI – trnS-GCU*) | 8,669 (IGS *psbI – trnS-GCU*) | 26 | F | LSC |
| 12 | 4,848 (IGS *trnK-UUU* – *rps16*) | 8,839 (IGS *trnS-GCU – trnG-UCC*) | 25 | F | LSC |
| 13 | 4,851 (IGS *trnK-UUU* – *rps16*) | 4,851 (IGS *trnK-UUU* – *rps16*) | 25 | R | LSC |
| 14 | 4,851 (IGS *trnK-UUU* – *rps16*) | 4,854 (IGS *trnK-UUU* – *rps16*) | 25 | F | LSC |
| 15 | 4,854 (IGS *trnK-UUU* – *rps16*) | 4,854 (IGS *trnK-UUU* – *rps16*) | 25 | R | LSC |
| 16 | 40,148 (*psaB*) | 42,372 (*psaA*) | 25 | F | LSC |
| 17 | 57,390 (IGS *atpB* – *rbcL*) | 57,414 (IGS *atpB* – *rbcL*) | 25 | F | LSC |
| 18 | 115,664 (IGS *rpI32 – trnL-UAG*) | 115,664 (IGS *rpI32 – trnL-UAG*) | 25 | R | SSC |
| ***Acer pseudoplatanus*** | | | | | |
| 1 | 85,812 (*rps19*) | 129,858 (*ycf1*) | 26,075 | P | LSC; SSC (IRs) |
| 2 | 36,749 (IGS *trnS-UGA – psbZ*) | 36,799 (IGS *trnS-UGA – psbZ*) | 50 | F | LSC |
| 3 | 75,823 (IGS *psbT – psbN*) | 75,823 (IGS *psbT – psbN*) | 44 | P | LSC |
| 4 | 40,129 (*psaB*) | 42,353 (*psaA*) | 41 | F | LSC |
| 5 | 8,827 (IGS *psbI* – *trnS-GCU*) | 46,891 (*trnS-GGA*) | 30 | P | LSC |
| 6 | 114,991 (*rpl32*) | 114,991 (*rpl32*) | 29 | R | SSC |
| ***Acer rubrum*** | | | | | |
| 1 | 85,383 (*rps19*) | 129,576 (*ycf1*) | 26,107 | P | LSC; SSC (IRs) |
| 2 | 75,359 (IGS *psbT – psbN*) | 75,359 (IGS *psbT – psbN*) | 44 | P | LSC |
| 3 | 39,461 (*psaB*) | 41,685 (*psaA*) | 41 | F | LSC |
| 4 | 8,438 (IGS *psbI* – *trnS-GCU*) | 46,227 (*trnS-GGA*) | 30 | P | LSC |
| 5 | 32,014 (IGS *trnT-GGU* – *psbD*) | 32,040 (IGS *trnT-GGU* – *psbD*) | 27 | F | LSC |
| ***Acer sterculiaceum* subsp*. sterculiaceum*** | | | | | |
| 1 | 86,014 (*rps19*) | 130,160 (*ycf1*) | 26,098 | P | LSC; SSC (IRs) |
| 2 | 50,335 (IGS *trnF-GAA – ndhJ*) | 50,390 (IGS *trnF-GAA – ndhJ*) | 56 | F | LSC |
| 3 | 40,110 (*psaB*) | 42,334 (*psaA*) | 41 | F | LSC |
| 4 | 68,643 (IGS *trnP-UGG – psaJ*) | 68,678 (IGS *trnP-UGG – psaJ*) | 35 | F | LSC |
| 5 | 8,778 (IGS *psbI* – *trnS-GCU*) | 46,852 (*trnS-GGA*) | 30 | P | LSC |
| 6 | 37,623 (IGS *psbZ – trnG-GCC*) | 37,623 (IGS *psbZ – trnG-GCC*) | 28 | P | LSC |
| ***Acer tataricum* subsp. *ginnala*** | | | | | |
| 1 | 85,404 (*rps19*) | 129,566 (*ycf1*) | 26,101 | P | LSC; SSC (IRs) |
| 2 | 40,037 (*psaB*) | 42,261 (*psaA*) | 41 | F | LSC |
| 3 | 118,597 (IGS *ndhD – psaC*) | 118,597 (IGS *ndhD – psaC*) | 34 | P | SSC |
| 4 | 8,721 (IGS *psbI* – *trnS-GCU*) | 46,786 (*trnS-GGA*) | 30 | P | LSC |
| 5 | 114,975 (IGS *rpI32 – trnL-UAG*) | 114,975 (IGS *rpI32 – trnL-UAG*) | 29 | R | SSC |
