# Supplementary material for: Plastome comparative genomics in maples resolves the infrageneric backbone relationships
Source: PeerJ. 2020 Jul 13;8:e9483. doi: 10.7717/peerj.9483 (PMC7365138; doi:10.7717/peerj.9483)
Supplement: Table S4 — See Table S1 for full species names. [file peerj-08-9483-s004.docx]

**Table S4.** Number of simple sequence repeats (SSRs) found in *Acer* plastomes using MISA-web. See Table S1 for full species names.

| **Motif** | ***A. acum*** | ***A. carp*** | ***A. glab*** | ***A. maxi*** | ***A. micr*** | ***A. negu*** | ***A. nipp*** | ***A. oblo*** | ***A. palm*** | ***A. pent*** | ***A. pilo*** | ***A. plat*** | ***A. pseu*** | ***A. rubr*** | ***A. ster*** | ***A. tata*** | **Total** | **%** |
| --- | --- | --- | --- | --- | --- | --- | --- | --- | --- | --- | --- | --- | --- | --- | --- | --- | --- | --- |
| (A)10 | 13 | 13 | 18 | 14 | 15 | 22 | 13 | 11 | 12 | 12 | 13 | 15 | 12 | 13 | 18 | 20 | **234** | **20.4** |
| (A)11 | 6 | 10 | 9 | 7 | 10 | 7 | 8 | 8 | 10 | 8 | 12 | 8 | 9 | 9 | 8 | 7 | **136** | **11.8** |
| (A)12 | 2 | 3 | 2 | 4 | 2 | 2 | 5 | 5 | 4 | 3 | 1 | 3 | 3 | 3 | 2 | 2 | **46** | **4.0** |
| (A)13 | 1 | 2 | 1 | 1 | 2 | 0 | 2 | 1 | 5 | 2 | 2 | 4 | 5 | 1 | 3 | 1 | **33** | **2.9** |
| (A)14 | 0 | 0 | 3 | 0 | 2 | 2 | 3 | 1 | 1 | 2 | 1 | 1 | 3 | 1 | 3 | 0 | **23** | **2.0** |
| (A)15 | 1 | 0 | 3 | 1 | 0 | 1 | 3 | 3 | 0 | 1 | 0 | 1 | 0 | 1 | 1 | 0 | **16** | **1.4** |
| (A)16 | 0 | 1 | 1 | 0 | 4 | 0 | 1 | 0 | 1 | 1 | 0 | 1 | 0 | 0 | 1 | 0 | **11** | **1.0** |
| (A)17 | 0 | 0 | 0 | 0 | 1 | 0 | 0 | 0 | 0 | 1 | 0 | 1 | 0 | 0 | 0 | 1 | **4** | **0.3** |
| (A)18 | 1 | 0 | 1 | 1 | 0 | 0 | 0 | 1 | 0 | 0 | 0 | 0 | 0 | 0 | 0 | 1 | **5** | **0.4** |
| (A)19 | 0 | 0 | 0 | 0 | 0 | 0 | 0 | 0 | 0 | 0 | 0 | 0 | 0 | 0 | 0 | 1 | **1** | **0.1** |
| (A)22 | 0 | 0 | 0 | 0 | 0 | 0 | 0 | 0 | 0 | 0 | 0 | 1 | 0 | 0 | 0 | 0 | **1** | **0.1** |
| (A)28 | 0 | 0 | 0 | 0 | 0 | 0 | 0 | 0 | 0 | 0 | 0 | 1 | 0 | 0 | 0 | 0 | **1** | **0.1** |
| (C)10 | 0 | 0 | 0 | 0 | 1 | 2 | 1 | 2 | 0 | 0 | 1 | 1 | 0 | 1 | 0 | 0 | **9** | **0.8** |
| (C)11 | 0 | 0 | 1 | 0 | 0 | 0 | 0 | 0 | 0 | 0 | 1 | 0 | 0 | 0 | 0 | 0 | **2** | **0.2** |
| (C)12 | 0 | 1 | 0 | 1 | 0 | 0 | 0 | 0 | 0 | 0 | 0 | 0 | 0 | 0 | 1 | 0 | **3** | **0.3** |
| (C)13 | 0 | 0 | 0 | 1 | 1 | 0 | 0 | 0 | 1 | 0 | 0 | 0 | 0 | 1 | 0 | 1 | **5** | **0.4** |
| (C)14 | 1 | 0 | 0 | 0 | 0 | 0 | 0 | 1 | 0 | 1 | 0 | 1 | 0 | 0 | 0 | 0 | **4** | **0.3** |
| (G)10 | 1 | 0 | 0 | 0 | 0 | 0 | 0 | 0 | 0 | 0 | 0 | 1 | 0 | 0 | 1 | 0 | **3** | **0.3** |
| (T)10 | 16 | 16 | 24 | 12 | 22 | 18 | 19 | 14 | 13 | 16 | 15 | 19 | 18 | 16 | 21 | 17 | **276** | **24.0** |
| (T)11 | 9 | 10 | 11 | 7 | 12 | 9 | 18 | 6 | 11 | 11 | 7 | 14 | 11 | 8 | 6 | 10 | **160** | **13.9** |
| (T)12 | 2 | 5 | 7 | 5 | 5 | 4 | 2 | 3 | 3 | 3 | 1 | 2 | 1 | 6 | 5 | 0 | **54** | **4.7** |
| (T)13 | 1 | 1 | 4 | 3 | 2 | 1 | 2 | 3 | 5 | 3 | 2 | 3 | 5 | 2 | 2 | 2 | **41** | **3.6** |
| (T)14 | 2 | 2 | 4 | 2 | 2 | 1 | 1 | 1 | 2 | 0 | 2 | 2 | 4 | 1 | 1 | 1 | **28** | **2.4** |
| (T)15 | 1 | 1 | 0 | 0 | 1 | 2 | 0 | 0 | 1 | 0 | 0 | 1 | 0 | 0 | 0 | 1 | **8** | **0.7** |
| (T)16 | 0 | 0 | 1 | 1 | 0 | 0 | 1 | 1 | 0 | 1 | 1 | 1 | 0 | 1 | 0 | 0 | **8** | **0.7** |
| (T)17 | 1 | 0 | 0 | 1 | 0 | 0 | 0 | 0 | 0 | 0 | 0 | 1 | 0 | 0 | 0 | 0 | **3** | **0.3** |
| (T)18 | 1 | 0 | 0 | 0 | 1 | 0 | 0 | 0 | 0 | 0 | 0 | 1 | 0 | 0 | 0 | 1 | **4** | **0.3** |
| (T)24 | 0 | 0 | 0 | 0 | 0 | 0 | 0 | 0 | 0 | 0 | 1 | 0 | 0 | 0 | 0 | 0 | **1** | **0.1** |
| (AT)6 | 1 | 1 | 1 | 2 | 0 | 0 | 0 | 2 | 0 | 2 | 0 | 1 | 3 | 0 | 1 | 1 | **15** | **1.3** |
| (AT)7 | 0 | 0 | 0 | 0 | 0 | 0 | 0 | 0 | 1 | 0 | 0 | 1 | 0 | 0 | 0 | 0 | **2** | **0.2** |
| (AT)8 | 0 | 0 | 0 | 0 | 0 | 0 | 0 | 0 | 1 | 0 | 0 | 0 | 0 | 0 | 0 | 0 | **1** | **0.1** |
| (AT)9 | 0 | 0 | 0 | 0 | 0 | 0 | 0 | 0 | 0 | 0 | 0 | 0 | 0 | 1 | 0 | 0 | **1** | **0.1** |
| (TA)6 | 0 | 0 | 0 | 0 | 0 | 0 | 2 | 0 | 0 | 0 | 0 | 0 | 0 | 0 | 0 | 0 | **2** | **0.2** |
| (TA)8 | 0 | 0 | 0 | 0 | 0 | 0 | 0 | 0 | 0 | 0 | 0 | 0 | 1 | 0 | 0 | 0 | **1** | **0.1** |
| (AAT)5 | 0 | 0 | 0 | 0 | 0 | 1 | 1 | 1 | 0 | 0 | 0 | 0 | 0 | 0 | 0 | 0 | **3** | **0.3** |
| (ATA)6 | 0 | 0 | 1 | 0 | 0 | 0 | 0 | 0 | 0 | 0 | 0 | 0 | 0 | 0 | 0 | 0 | **1** | **0.1** |
| (TAT)5 | 0 | 0 | 0 | 0 | 0 | 0 | 0 | 0 | 0 | 0 | 0 | 0 | 0 | 0 | 0 | 1 | **1** | **0.1** |
| (TAT)6 | 0 | 0 | 0 | 0 | 0 | 0 | 0 | 1 | 0 | 0 | 0 | 0 | 0 | 0 | 0 | 0 | **1** | **0.1** |
| (TAAA)5 | 0 | 0 | 0 | 0 | 1 | 0 | 0 | 0 | 0 | 0 | 0 | 0 | 0 | 0 | 0 | 0 | **1** | **0.1** |
| **Total** | **60** | **66** | **92** | **63** | **84** | **72** | **82** | **65** | **71** | **67** | **60** | **85** | **75** | **65** | **74** | **68** | **1149** |  |
