# Supplementary material for: Plastome comparative genomics in maples resolves the infrageneric backbone relationships
Source: PeerJ. 2020 Jul 13;8:e9483. doi: 10.7717/peerj.9483 (PMC7365138; doi:10.7717/peerj.9483)
Supplement: Table S5 — C: compound repeat, p1: monomeric repeat, p2: dimeric repeat, p3: trimeric repeat, p4: tetrameric repeat; IGS: intergenic spacer, IR: inverted repeat, LSC: large single copy, SSC: small single copy. [file peerj-08-9483-s005.docx]

**Table S5.** Simple sequence repeats (SSRs) found in *Acer* plastomes using MISA-web. C: compound repeat, p1: monomeric repeat, p2: dimeric repeat, p3: trimeric repeat, p4: tetrameric repeat; IGS: intergenic spacer, IR: inverted repeat, LSC: large single copy, SSC: small single copy.

| **Fre-quency** | **SSR type** | **SSR** | **Size (bp)** | **Start** | **End** | **SSR location** | **Region** |
| --- | --- | --- | --- | --- | --- | --- | --- |
| *Acer acuminatum* | | | | | | |  |
| 12 | p1 | A | 10 | 6,284 | 6,293 | *rps16* | LSC |
|  | p1 | A | 10 | 7,046 | 7,055 | IGS (*rps16 – trnQ-UUG*) | LSC |
|  | p1 | A | 10 | 8,115 | 8,124 | IGS (*psbK – psbI)* | LSC |
|  | p1 | A | 10 | 36,934 | 36,943 | IGS (*psbZ – trnG-GCC*) | LSC |
|  | p1 | A | 10 | 52,798 | 52,807 | IGS (*ndhC – trnV-UAC)* | LSC |
|  | p1 | A | 10 | 61,102 | 61,111 | *psaI* | LSC |
|  | p1 | A | 10 | 61,290 | 61,299 | IGS (*psaI – ycf4*) | LSC |
|  | p1 | A | 10 | 65,760 | 65,769 | *psbF* | LSC |
|  | p1 | A | 10 | 111,401 | 111,410 | *ndhF*; *ycf1* | IRb |
|  | p1 | A | 10 | 122,016 | 122,025 | *ndhA* | SSC |
|  | p1 | A | 10 | 129,660 | 129,669 | *ycf1* | IRa |
|  | p1 | A | 10 | 131,573 | 131,582 | IGS (*trnN-GUU – trnR-ACG*) | IRa |
| 5 | p1 | A | 11 | 366 | 376 | IGS (*trnH-GUG – psbA*) | LSC |
|  | p1 | A | 11 | 5,635 | 5,645 | *rps16* | LSC |
|  | p1 | A | 11 | 114,760 | 114,770 | IGS (*rpl32 – trnL-UAG*) | SSC |
|  | p1 | A | 11 | 127,435 | 127,445 | *ycf1* | SSC |
|  | p1 | A | 11 | 129,318 | 129,328 | *ycf1* | SSC |
| 2 | p1 | A | 12 | 4,867 | 4,878 | IGS (*trnK-UUU – rps16*) | LSC |
|  | p1 | A | 12 | 15,185 | 15,196 | IGS (*atpH – atpI*) | LSC |
| 1 | p1 | A | 13 | 48,361 | 48,373 | IGS (*trnT-UGU – trnL-UAA*) | LSC |
| 1 | p1 | A | 15 | 82,224 | 82,238 | IGS (*rpl14 – rpl16*) | LSC |
| 1 | p1 | A | 18 | 108,913 | 108,930 | IGS (*rrn5 – trnR-ACG*) | IRb |
| 1 | p1 | C | 14 | 80,918 | 80,931 | *infA* | LSC |
| 1 | p1 | G | 10 | 67,320 | 67,329 | IGS (*petG – trnW-CCA*) | LSC |
| 14 | p1 | T | 10 | 9,054 | 9,063 | IGS (*trnS-GCU – trnG-UCC*) | LSC |
|  | p1 | T | 10 | 12,989 | 12,998 | *atpF* | LSC |
|  | p1 | T | 10 | 15,069 | 15,078 | IGS (*atpH – atpI*) | LSC |
|  | p1 | T | 10 | 26,420 | 26,429 | *rpoB* | LSC |
|  | p1 | T | 10 | 27,890 | 27,899 | IGS (*rpoB – trnC-GCA*) | LSC |
|  | p1 | T | 10 | 48,071 | 48,080 | IGS (*trnT-UGU – trnL-UAA*) | LSC |
|  | p1 | T | 10 | 53,106 | 53,115 | *trnV-UAC* | LSC |
|  | p1 | T | 10 | 55,992 | 56,001 | *atpB* | LSC |
|  | p1 | T | 10 | 61,494 | 61,503 | IGS (*psaI – ycf4*) | LSC |
|  | p1 | T | 10 | 69,012 | 69,021 | IGS (*rpl33 – rps18*) | LSC |
|  | p1 | T | 10 | 109,325 | 109,334 | IGS (*trnR-ACG – trnN-GUU*) | IRb |
|  | p1 | T | 10 | 111,238 | 111,247 | *ycf1* | IRb |
|  | p1 | T | 10 | 122,396 | 122,405 | *ndhA* | SSC |
|  | p1 | T | 10 | 129,497 | 129,506 | *ycf1* | IRa |
| 7 | p1 | T | 11 | 8,243 | 8,253 | IGS (*psbK – psbI*) | LSC |
|  | p1 | T | 11 | 51,678 | 51,688 | IGS (*ndhK – ndhC*) | LSC |
|  | p1 | T | 11 | 72,576 | 72,586 | *clpP* | LSC |
|  | p1 | T | 11 | 118,544 | 118,554 | IGS (*ndhD – psaC*) | SSC |
|  | p1 | T | 11 | 124,824 | 124,834 | *rps15* | SSC |
|  | p1 | T | 11 | 125,618 | 125,628 | *ycf1* | SSC |
|  | p1 | T | 11 | 128,019 | 128,029 | *ycf1* | SSC |
| 2 | p1 | T | 12 | 29,834 | 29,845 | IGS (*psbM – trnD-GUC*) | LSC |
|  | p1 | T | 12 | 66,400 | 66,411 | IGS (*psbE – petL*) | LSC |
| 1 | p1 | T | 13 | 23,174 | 23,186 | *rpoC1* | LSC |
| 2 | p1 | T | 14 | 18,786 | 18,799 | *rpoC2* | LSC |
|  | p1 | T | 14 | 71,590 | 71,603 | *clpP* | LSC |
| 1 | p1 | T | 15 | 114,896 | 114,910 | IGS (*rpl32 – trnL-UAG*) | SSC |
| 1 | p1 | T | 17 | 14,833 | 14,849 | IGS (*atpH – atpI*) | LSC |
| 1 | p1 | T | 18 | 131,977 | 131,994 | IGS (*trnR-ACG – rrn5*) | IRa |
| 1 | p2 | (AT)6 | 12 | 31,833 | 31,844 | IGS (*trnE-UUC – trnT-GGU*) | LSC |
| 1 | c | (T)10 ... (A)11 | 113 | 128,556 | 128,668 | *ycf1* | SSC |
| 1 | c | (T)10 G (T)11 | 22 | 1,761 | 1,782 | IGS (*psbA – trnK-UUU*) | LSC |
| 1 | c | (A)10 ... (T)11 | 42 | 122,678 | 122,719 | *ndhA* | SSC |
| *Acer carpinifolium* | | | | | | |  |
| 11 | p1 | A | 10 | 4,786 | 4,795 | IGS (*trnK-UUU – rps16*) | LSC |
|  | p1 | A | 10 | 13,348 | 13,357 | *atpF* | LSC |
|  | p1 | A | 10 | 13,841 | 13,850 | IGS (*atpF – atpH*) | LSC |
|  | p1 | A | 10 | 52,482 | 52,491 | IGS (*ndhC – trnV-UAC*) | LSC |
|  | p1 | A | 10 | 61,174 | 61,183 | *psaI* | LSC |
|  | p1 | A | 10 | 61,362 | 61,371 | IGS (*psaI – ycf4*) | LSC |
|  | p1 | A | 10 | 65,828 | 65,837 | *psbF* | LSC |
|  | p1 | A | 10 | 111,441 | 111,450 | *ycf1; ndhF* | IRb |
|  | p1 | A | 10 | 113,960 | 113,969 | IGS (*ndhF – rpl32*) | SSC |
|  | p1 | A | 10 | 121,698 | 121,707 | *ndhA* | SSC |
|  | p1 | A | 10 | 129,374 | 129,383 | *ycf1* | IRa |
| 9 | p1 | A | 11 | 5,714 | 5,724 | *rps16* | LSC |
|  | p1 | A | 11 | 8,878 | 8,888 | IGS (*trnS-GCU –trnG-UCC*) | LSC |
|  | p1 | A | 11 | 10,566 | 10,576 | IGS (*trnG-UCC – trnR-UCU*) | LSC |
|  | p1 | A | 11 | 43,688 | 43,698 | IGS (*psaA – ycf3*) | LSC |
|  | p1 | A | 11 | 56,650 | 56,660 | IGS (*atpB – rbcL*) | LSC |
|  | p1 | A | 11 | 78,074 | 78,084 | *petD* | LSC |
|  | p1 | A | 11 | 127,155 | 127,165 | *ycf1* | SSC |
|  | p1 | A | 11 | 128,378 | 128,388 | *ycf1* | SSC |
|  | p1 | A | 11 | 129,032 | 129,042 | *ycf1* | SSC |
| 3 | p1 | A | 12 | 48,465 | 48,476 | IGS (*trnT-UGU – trnL-UAA*) | LSC |
|  | p1 | A | 12 | 108,987 | 108,998 | IGS (*rrn5 – trnR-ACG*) | IRb |
|  | p1 | A | 12 | 124,788 | 124,799 | IGS (*rps15 – ycf1*) | SSC |
| 2 | p1 | A | 13 | 15,260 | 15,272 | IGS (*atpH – atpI*) | LSC |
|  | p1 | A | 13 | 82,310 | 82,322 | IGS (*rpl14 – rpl16*) | LSC |
| 1 | p1 | A | 16 | 37,377 | 37,392 | IGS (*psbZ – trnG-GCC*) | LSC |
| 1 | p1 | C | 12 | 81,004 | 81,015 | *infA* | LSC |
| 14 | p1 | T | 10 | 13,040 | 13,049 | *atpF* | LSC |
|  | p1 | T | 10 | 23,612 | 23,621 | *rpoC1* | LSC |
|  | p1 | T | 10 | 26,855 | 26,864 | *rpoB* | LSC |
|  | p1 | T | 10 | 49,437 | 49,446 | IGS (*trnL-UAA – trnF-GAA*) | LSC |
|  | p1 | T | 10 | 50,365 | 50,374 | IGS (*trnF-GAA – ndhJ*) | LSC |
|  | p1 | T | 10 | 56,089 | 56,098 | *atpB* | LSC |
|  | p1 | T | 10 | 60,409 | 60,418 | IGS (*accD – psaI*) | LSC |
|  | p1 | T | 10 | 61,565 | 61,574 | IGS (*psaI – ycf4*) | LSC |
|  | p1 | T | 10 | 69,091 | 69,100 | IGS (*rpl33 – rps18*) | LSC |
|  | p1 | T | 10 | 71,504 | 71,513 | *clpP* | LSC |
|  | p1 | T | 10 | 75,159 | 75,168 | IGS (*psbB – psbT*) | LSC |
|  | p1 | T | 10 | 111,278 | 111,287 | *ycf1* | IRb |
|  | p1 | T | 10 | 127,865 | 127,874 | *ycf1* | SSC |
|  | p1 | T | 10 | 129,211 | 129,220 | *ycf1* | IRa |
| 10 | p1 | T | 11 | 9,307 | 9,317 | IGS (*trnS-GCU – trnG-UCC*) | LSC |
|  | p1 | T | 11 | 30,096 | 30,106 | IGS (*petN – psbM*) | LSC |
|  | p1 | T | 11 | 48,040 | 48,050 | IGS (*trnT-UGU – trnL-UAA*) | LSC |
|  | p1 | T | 11 | 56,444 | 56,454 | IGS (*atpB – rbcL*) | LSC |
|  | p1 | T | 11 | 72,580 | 72,590 | *clpP* | LSC |
|  | p1 | T | 11 | 118,214 | 118,224 | IGS (*ndhD – psaC*) | SSC |
|  | p1 | T | 11 | 122,099 | 122,109 | *ndhA* | SSC |
|  | p1 | T | 11 | 124,535 | 124,545 | *rps15* | SSC |
|  | p1 | T | 11 | 125,338 | 125,348 | *ycf1* | SSC |
|  | p1 | T | 11 | 127,739 | 127,749 | *ycf1* | SSC |
| 5 | p1 | T | 12 | 8,321 | 8,332 | IGS (*psbK – psbI*) | LSC |
|  | p1 | T | 12 | 30,284 | 30,295 | IGS (*psbM – trnD-GUC*) | LSC |
|  | p1 | T | 12 | 66,474 | 66,485 | IGS (*psbE – petL*) | LSC |
|  | p1 | T | 12 | 114,554 | 114,565 | IGS (*rpl32 – trnL-UAG*) | SSC |
|  | p1 | T | 12 | 131,663 | 131,674 | IGS (*trnR-ACG – rrn5*) | IRa |
| 2 | p1 | T | 14 | 19,195 | 19,208 | *rpoC2* | LSC |
|  | p1 | T | 14 | 53,198 | 53,211 | *trnV-UAC* | LSC |
| 1 | p1 | T | 15 | 51,804 | 51,818 | IGS (*ndhK – ndhC*) | LSC |
| 1 | p2 | (AT)6 | 12 | 32,291 | 32,302 | IGS (*trnE-UUC – trnT-GGU*) | LSC |
| 1 | c | (A)10 ... (A)11 | 118 | 114,302 | 114,419 | IGS (*rpl32 – trnL-UAG*) | SSC |
| 1 | c | (A)10 ... (T)13 | 40 | 122,382 | 122,421 | *ndhA* | SSC |
| 1 | c | (T)10 ... (T)10 | 62 | 1,732 | 1,793 | IGS (*psbA – trnK-UUU*) | LSC |
| *Acer glabrum* | | | | | | |  |
| 16 | p1 | A | 10 | 3,784 | 3,793 | *trnK-UUU* | LSC |
|  | p1 | A | 10 | 8,295 | 8,304 | IGS (*psbK – psbI*) | LSC |
|  | p1 | A | 10 | 10,703 | 10,712 | IGS (*trnG-UCC – trnR-UCU*) | LSC |
|  | p1 | A | 10 | 13,514 | 13,523 | *atpF* | LSC |
|  | p1 | A | 10 | 14,007 | 14,016 | IGS (*atpF – atpH*) | LSC |
|  | p1 | A | 10 | 33,637 | 33,646 | IGS (*trnT-GGU – psbD*) | LSC |
|  | p1 | A | 10 | 61,804 | 61,813 | *psaI* | LSC |
|  | p1 | A | 10 | 66,458 | 66,467 | *psbF* | LSC |
|  | p1 | A | 10 | 72,943 | 72,952 | *clpP* | LSC |
|  | p1 | A | 10 | 74,135 | 74,144 | IGS (*clpP – psbB*) | LSC |
|  | p1 | A | 10 | 112,087 | 112,096 | *ycf1*; *ndhF* | IRb |
|  | p1 | A | 10 | 114,653 | 114,662 | IGS (*ndhF – rpl32*) | SSC |
|  | p1 | A | 10 | 125,893 | 125,902 | IGS (*rps15 – ycf1*) | SSC |
|  | p1 | A | 10 | 130,484 | 130,493 | *ycf1* | IRa |
|  | p1 | A | 10 | 132,397 | 132,406 | IGS (*trnN-GUU – trnR-ACG*) | IRa |
|  | p1 | A | 10 | 141,746 | 141,755 | IGS (*ycf15 – rps12*) | IRa |
| 8 | p1 | A | 11 | 37,610 | 37,620 | IGS (*psbZ – trnG-GCC*) | LSC |
|  | p1 | A | 11 | 46,793 | 46,803 | IGS (*ycf3 – trnS-GGA*) | LSC |
|  | p1 | A | 11 | 49,148 | 49,158 | IGS (*trnT-UGU – trnL-UAA*) | LSC |
|  | p1 | A | 11 | 53,594 | 53,604 | IGS (*ndhC – trnV-UAC*) | LSC |
|  | p1 | A | 11 | 57,358 | 57,368 | IGS (*atpB – rbcL*) | LSC |
|  | p1 | A | 11 | 122,769 | 122,779 | *ndhA* | SSC |
|  | p1 | A | 11 | 128,268 | 128,278 | *ycf1* | SSC |
|  | p1 | A | 11 | 130,133 | 130,143 | *ycf1* | SSC |
| 2 | p1 | A | 12 | 109,596 | 109,607 | IGS (*rrn5 – trnR-ACG*) | IRb |
|  | p1 | A | 12 | 129,479 | 129,490 | *ycf1* | SSC |
| 2 | p1 | A | 15 | 78,711 | 78,725 | *petD* | LSC |
|  | p1 | A | 15 | 115,020 | 115,034 | IGS (*ndhF – rpl32*) | SSC |
| 1 | p1 | A | 16 | 15,429 | 15,444 | IGS (*atpH – atpI*) | LSC |
| 1 | p1 | A | 18 | 43,885 | 43,902 | IGS (*psaA – ycf3*) | LSC |
| 1 | p1 | C | 11 | 81,648 | 81,658 | *infA* | LSC |
| 20 | p1 | T | 10 | 9,256 | 9,265 | IGS (*trnS-GCU – trnG-UCC*) | LSC |
|  | p1 | T | 10 | 9,489 | 9,498 | IGS (*trnS-GCU – trnG-UCC*) | LSC |
|  | p1 | T | 10 | 9,700 | 9,709 | IGS (*trnS-GCU – trnG-UCC*) | LSC |
|  | p1 | T | 10 | 12,610 | 12,619 | IGS (*atpA – atpF*) | LSC |
|  | p1 | T | 10 | 27,053 | 27,062 | *rpoB* | LSC |
|  | p1 | T | 10 | 30,297 | 30,306 | IGS (*petN – psbM*) | LSC |
|  | p1 | T | 10 | 32,187 | 32,196 | IGS (*trnE-UUC – trnT-GGU*) | LSC |
|  | p1 | T | 10 | 32,735 | 32,744 | IGS (*trnT-GGU – psbD*) | LSC |
|  | p1 | T | 10 | 48,606 | 48,615 | IGS (*trnT-UGU – trnL-UAA*) | LSC |
|  | p1 | T | 10 | 56,791 | 56,800 | *atpB* | LSC |
|  | p1 | T | 10 | 62,187 | 62,196 | IGS (*psaI – ycf4*) | LSC |
|  | p1 | T | 10 | 62,970 | 62,979 | IGS (*ycf4 – cemA*) | LSC |
|  | p1 | T | 10 | 79,692 | 79,701 | IGS (*petD – rpoA*) | LSC |
|  | p1 | T | 10 | 100,653 | 100,662 | IGS (*rps12 – ycf15*) | IRb |
|  | p1 | T | 10 | 110,002 | 110,011 | IGS (*trnR-ACG – trnN-GUU*) | IRb |
|  | p1 | T | 10 | 111,915 | 111,924 | *ycf1* | IRb |
|  | p1 | T | 10 | 115,773 | 115,782 | IGS (*rpl32 – trnL-UAG*) | SSC |
|  | p1 | T | 10 | 119,295 | 119,304 | IGS (*ndhD – psaC*) | SSC |
|  | p1 | T | 10 | 127,121 | 127,130 | *ycf1* | SSC |
|  | p1 | T | 10 | 130,312 | 130,321 | *ycf1* | IRa |
| 9 | p1 | T | 11 | 13,202 | 13,212 | *atpF* | LSC |
|  | p1 | T | 11 | 48,722 | 48,733 | IGS (*trnT-UGU – trnL-UAA*) | LSC |
|  | p1 | T | 11 | 69,766 | 69,776 | IGS (*rpl33 – rps18*) | LSC |
|  | p1 | T | 11 | 75,860 | 75,870 | IGS (*psbB – psbT*) | LSC |
|  | p1 | T | 11 | 81,906 | 81,916 | IGS (*infA – rps8*) | LSC |
|  | p1 | T | 11 | 121,881 | 121,891 | IGS (*ndhI – ndhA*) | SSC |
|  | p1 | T | 11 | 125,625 | 125,635 | *rps15* | SSC |
|  | p1 | T | 11 | 126,442 | 126,452 | *ycf1* | SSC |
|  | p1 | T | 11 | 128,840 | 128,850 | *ycf1* | SSC |
| 6 | p1 | T | 12 | 17,444 | 17,455 | *rpoC2* | LSC |
|  | p1 | T | 12 | 28,535 | 28,546 | IGS (*rpoB – trnC-GCA*) | LSC |
|  | p1 | T | 12 | 53,903 | 53,914 | *trnV-UAC* | LSC |
|  | p1 | T | 12 | 73,272 | 73,283 | *clpP* | LSC |
|  | p1 | T | 12 | 128,558 | 128,569 | *ycf1* | SSC |
|  | p1 | T | 12 | 132,801 | 132,812 | IGS (*trnR-ACG – rrn5*) | IRa |
| 2 | p1 | T | 13 | 51,053 | 51,065 | IGS (*trnF-GAA – ndhJ*) | LSC |
|  | p1 | T | 13 | 52,504 | 52,516 | IGS (*ndhK – ndhC*) | LSC |
| 4 | p1 | T | 14 | 19,358 | 19,371 | *rpoC2* | LSC |
|  | p1 | T | 14 | 30,484 | 30,497 | IGS (*psbM – trnD-GUC*) | LSC |
|  | p1 | T | 14 | 115,644 | 115,657 | IGS (*rpl32 – trnL-UAG*) | SSC |
|  | p1 | T | 14 | 123,192 | 123,205 | *ndhA* | SSC |
| 1 | p1 | T | 16 | 122,898 | 122,913 | *ndhA* | SSC |
| 1 | p2 | (AT)6 | 12 | 32,527 | 32,538 | IGS (*trnE-UUC – trnT-GGU*) | LSC |
| 1 | c | (A)10 ... (A)14 | 110 | 4,932 | 5,041 | IGS (*trnK-UUU – rps16*) | LSC |
| 1 | c | (A)10 ... (T)11 | 42 | 123,479 | 123,520 | *ndhA* | SSC |
| 1 | c | (A)14 ... (T)13 | 110 | 115,367 | 115,476 | IGS (*rpl32 – trnL-UAG*) | SSC |
| 1 | c | (A)14 TAT(ATA)6 | 35 | 8,974 | 9,008 | IGS (*trnS-GCU – trnG-UCC*) | LSC |
| 1 | c | (T)10 ... (A)11 | 56 | 65,772 | 65,827 | IGS (*petA – psbJ*) | LSC |
| 1 | c | (T)10 ... (A)13 | 79 | 67,106 | 67,184 | IGS (*psbE – petL*) | LSC |
| 1 | c | (T)10 ... (T)12 | 103 | 72,191 | 72,293 | *clpP* | LSC |
| 1 | c | (T)10 A (T)13 | 24 | 1,824 | 1,847 | IGS (*psbA – trnK-UUU*) | LSC |
| 1 | c | (T)11 ... (A)15 | 67 | 82,905 | 82,971 | IGS (*rpl14 – rpl16*) | LSC |
| *Acer maximowiczianum* | | | | | | |  |
| 11 | p1 | A | 10 | 380 | 389 | IGS (*trnH-GUG – psbA*) | LSC |
|  | p1 | A | 10 | 5,682 | 5,691 | *rps16* | LSC |
|  | p1 | A | 10 | 12,474 | 12,483 | IGS (*atpA – atpF*) | LSC |
|  | p1 | A | 10 | 37,334 | 37,343 | IGS (*psbZ – trnG-GCC*) | LSC |
|  | p1 | A | 10 | 46,559 | 46,568 | IGS (*ycf3 – trnS-GGA*) | LSC |
|  | p1 | A | 10 | 52,863 | 52,872 | IGS (*ndhC – trnV-UAC*) | LSC |
|  | p1 | A | 10 | 61,552 | 61,561 | *psaI* | LSC |
|  | p1 | A | 10 | 65,587 | 65,596 | IGS (*petA – psbJ*) | LSC |
|  | p1 | A | 10 | 66,223 | 66,232 | *psbF* | LSC |
|  | p1 | A | 10 | 115,269 | 115,278 | IGS (*rpl32 – trnL-UAG*) | SSC |
|  | p1 | A | 10 | 130,208 | 130,217 | *ycf1* | IRa |
| 6 | p1 | A | 11 | 15,254 | 15,264 | IGS (*atpH – atpI*) | LSC |
|  | p1 | A | 11 | 61,741 | 61,751 | IGS (*psaI – ycf4*) | LSC |
|  | p1 | A | 11 | 114,821 | 114,831 | IGS (*ndhF – rpl32*) | SSC |
|  | p1 | A | 11 | 127,989 | 127,999 | *ycf1* | SSC |
|  | p1 | A | 11 | 129,213 | 129,223 | *ycf1* | SSC |
|  | p1 | A | 11 | 129,866 | 129,876 | *ycf1* | SSC |
| 4 | p1 | A | 12 | 3,698 | 3,709 | *trnK-UUU* | LSC |
|  | p1 | A | 12 | 8,853 | 8,864 | IGS (*trnS-GCU – trnG-UCC*) | LSC |
|  | p1 | A | 12 | 48,918 | 48,929 | IGS (*trnT-UGU – trnL-UAA*) | LSC |
|  | p1 | A | 12 | 109,404 | 109,415 | IGS (*rrn5 – trnR-ACG*) | IRb |
| 1 | p1 | A | 13 | 53,277 | 53,289 | IGS (*ndhC – trnV-UAC*) | LSC |
| 1 | p1 | A | 15 | 82,672 | 82,686 | IGS (*rpl14 – rpl16*) | LSC |
| 1 | p1 | C | 12 | 50,330 | 50,341 | IGS (*trnF-GAA – ndhJ*) | LSC |
| 1 | p1 | C | 13 | 8,466 | 8,478 | IGS (*psbK – psbI*) | LSC |
| 10 | p1 | T | 10 | 9,122 | 9,131 | IGS (*trnS-GCU – trnG-UCC*) | LSC |
|  | p1 | T | 10 | 9,273 | 9,282 | IGS (*trnS-GCU – trnG-UCC*) | LSC |
|  | p1 | T | 10 | 26,832 | 26,841 | *rpoB* | LSC |
|  | p1 | T | 10 | 28,315 | 28,324 | IGS (*rpoB – trnC-GCA*) | LSC |
|  | p1 | T | 10 | 49,890 | 49,899 | IGS (*trnL-UAA – trnF-GAA*) | LSC |
|  | p1 | T | 10 | 56,479 | 56,488 | *atpB* | LSC |
|  | p1 | T | 10 | 82,132 | 82,141 | IGS (*rps8 – rpl14*) | LSC |
|  | p1 | T | 10 | 111,731 | 111,740 | *ycf1* | IRb |
|  | p1 | T | 10 | 122,933 | 122,942 | *ndhA* | SSC |
|  | p1 | T | 10 | 130,045 | 130,054 | *ycf1* | SSC-IRa |
| 4 | p1 | T | 11 | 53,590 | 53,600 | *trnV-UAC* | LSC |
|  | p1 | T | 11 | 119,060 | 119,070 | IGS (*ndhD – psaC*) | SSC |
|  | p1 | T | 11 | 125,360 | 125,370 | *rps15* | SSC |
|  | p1 | T | 11 | 128,573 | 128,583 | *ycf1* | SSC |
| 5 | p1 | T | 12 | 8,294 | 8,305 | IGS (*psbK – psbI*) | LSC |
|  | p1 | T | 12 | 30,258 | 30,269 | IGS (*psbM – trnD-GUC*) | LSC |
|  | p1 | T | 12 | 52,188 | 52,199 | IGS (*ndhK – ndhC*) | LSC |
|  | p1 | T | 12 | 66,863 | 66,874 | IGS (*psbE – petL*) | LSC |
|  | p1 | T | 12 | 132,533 | 132,544 | IGS (*trnR-ACG – rrn5*) | IRa |
| 3 | p1 | T | 13 | 14,908 | 14,920 | IGS (*atpH – atpI*) | LSC |
|  | p1 | T | 13 | 19,173 | 19,185 | *rpoC2* | LSC |
|  | p1 | T | 13 | 115,413 | 115,425 | IGS (*rpl32 – trnL-UAG*) | SSC |
| 2 | p1 | T | 14 | 73,015 | 73,028 | *clpP* | LSC |
|  | p1 | T | 14 | 122,647 | 122,660 | *ndhA* | SSC |
| 1 | p1 | T | 17 | 1,765 | 1,781 | IGS (*psbA – trnK-UUU*) | LSC |
| 2 | p2 | (AT)6 | 12 | 10,703 | 10,714 | IGS (*trnR-UCU – atpA*) | LSC |
|  | p2 | (AT)6 | 12 | 32,262 | 32,273 | IGS (*trnE-UUC – trnT-GGU*) | LSC |
| 1 | c | (A)10 ... (A)11 ... (A)18 | 184 | 4,761 | 4,944 | IGS (*trnK-UUU – rps16*) | LSC |
| 1 | c | (A)10 ... (T)10 | 41 | 123,215 | 123,255 | *ndhA* | SSC |
| 1 | c | (T)10 ... (T)16 | 107 | 71,930 | 72,036 | *clpP* | LSC |
| 1 | c | (T)11 ... (A)10 | 43 | 60,915 | 60,957 | IGS (*accD – psaI*) | LSC |
| 1 | c | (T)11 ... (T)11 | 47 | 23,587 | 23,633 | *rpoC1* | LSC |
| *Acer micranthum* | | | | | | |  |
| 12 | p1 | A | 10 | 380 | 389 | IGS (*trnH-GUG – psbA*) | LSC |
|  | p1 | A | 10 | 4,784 | 4,793 | IGS (*trnK-UUU – rps16*) | LSC |
|  | p1 | A | 10 | 4,950 | 4,959 | IGS (*trnK-UUU – rps16*) | LSC |
|  | p1 | A | 10 | 6,357 | 6,366 | *rps16* | LSC |
|  | p1 | A | 10 | 13,422 | 13,431 | *atpF* | LSC |
|  | p1 | A | 10 | 49,045 | 49,054 | IGS (*trnT-UGU – trnL-UAA*) | LSC |
|  | p1 | A | 10 | 53,071 | 53,080 | IGS (*ndhC – trnV-UAC*) | LSC |
|  | p1 | A | 10 | 61,801 | 61,810 | *psaI* | LSC |
|  | p1 | A | 10 | 66,461 | 66,470 | *psbF* | LSC |
|  | p1 | A | 10 | 114,745 | 114,754 | IGS (*ndhF – rpl32*) | SSC |
|  | p1 | A | 10 | 130,527 | 130,536 | *ycf1* | IRa |
|  | p1 | A | 10 | 156,358 | 156,367 | IGS (*rpl2 – trnH-GUG*) | IRa |
| 9 | p1 | A | 11 | 5,710 | 5,720 | *rps16* | LSC |
|  | p1 | A | 11 | 8,183 | 8,193 | IGS (*psbK – psbI*) | LSC |
|  | p1 | A | 11 | 46,689 | 46,699 | IGS (*ycf3 – trnS-GGA*) | LSC |
|  | p1 | A | 11 | 53,492 | 53,502 | IGS (*ndhC – trnV-UAC*) | LSC |
|  | p1 | A | 11 | 61,989 | 61,999 | IGS (*psaI – ycf4*) | LSC |
|  | p1 | A | 11 | 77,308 | 77,318 | *petB* | LSC |
|  | p1 | A | 11 | 128,305 | 128,315 | *ycf1* | SSC |
|  | p1 | A | 11 | 130,185 | 130,195 | *ycf1* | SSC |
|  | p1 | A | 11 | 141,801 | 141,811 | IGS (*ycf15 – rps12*) | IRa |
| 2 | p1 | A | 12 | 37,447 | 37,458 | IGS (*psbZ – trnG-GCC*) | LSC |
|  | p1 | A | 12 | 78,743 | 78,754 | *petD* | LSC |
| 2 | p1 | A | 13 | 109,692 | 109,704 | IGS (*rrn5 – trnR-ACG*) | IRb |
|  | p1 | A | 13 | 115,117 | 115,129 | IGS (*ndhF – rpl32*) | SSC |
| 1 | p1 | A | 14 | 3,723 | 3,736 | *trnK-UUU* | LSC |
| 3 | p1 | A | 16 | 15,285 | 15,300 | IGS (*atpH – atpI*) | LSC |
|  | p1 | A | 16 | 43,782 | 43,797 | IGS (*psaA – ycf3*) | LSC |
|  | p1 | A | 16 | 82,993 | 83,008 | IGS (*rpl14 – rpl16*) | LSC |
| 1 | p1 | A | 17 | 33,465 | 33,481 | IGS (*trnT-GGU – psbD*) | LSC |
| 1 | p1 | C | 10 | 17,282 | 17,291 | *rpoC2* | LSC |
| 1 | p1 | C | 13 | 50,463 | 50,475 | IGS (*trnF-GAA – ndhJ*) | LSC |
| 15 | p1 | T | 10 | 9,168 | 9,177 | IGS (*trnS-GCU – trnG-UCC*) | LSC |
|  | p1 | T | 10 | 9,380 | 9,389 | IGS (*trnS-GCU – trnG-UCC*) | LSC |
|  | p1 | T | 10 | 13,113 | 13,122 | *atpF* | LSC |
|  | p1 | T | 10 | 26,871 | 26,880 | *rpoB* | LSC |
|  | p1 | T | 10 | 28,362 | 28,371 | IGS (*rpoB – trnC-GCA*) | LSC |
|  | p1 | T | 10 | 32,575 | 32,584 | IGS (*trnT-GGU – psbD*) | LSC |
|  | p1 | T | 10 | 44,830 | 44,839 | *ycf3* | LSC |
|  | p1 | T | 10 | 48,622 | 48,631 | IGS (*trnT-UGU – trnL-UAA*) | LSC |
|  | p1 | T | 10 | 53,801 | 53,810 | *trnV-UAC* | LSC |
|  | p1 | T | 10 | 56,686 | 56,695 | *atpB* | LSC |
|  | p1 | T | 10 | 57,041 | 57,050 | IGS (*atpB – rbcL*) | LSC |
|  | p1 | T | 10 | 69,749 | 69,758 | IGS (*rpl33 – rps18*) | LSC |
|  | p1 | T | 10 | 86,180 | 86,189 | IGS (*rps19 – rpl2*) | IRb |
|  | p1 | T | 10 | 112,011 | 112,020 | *ycf1* | IRb |
|  | p1 | T | 10 | 125,449 | 125,458 | IGS (*ndhH – rps15*) | SSC |
| 10 | p1 | T | 11 | 14,956 | 14,966 | IGS (*atpH – atpI*) | LSC |
|  | p1 | T | 11 | 30,325 | 30,335 | IGS (*psbM – trnD-GUC*) | LSC |
|  | p1 | T | 11 | 61,029 | 61,039 | IGS (*accD – psaI*) | LSC |
|  | p1 | T | 11 | 73,283 | 73,293 | *clpP* | LSC |
|  | p1 | T | 11 | 100,736 | 100,746 | IGS (*rps12 – ycf15*) | IRb |
|  | p1 | T | 11 | 119,356 | 119,366 | IGS (*ndhD – psaC*) | SSC |
|  | p1 | T | 11 | 123,254 | 123,264 | *ndhA* | SSC |
|  | p1 | T | 11 | 125,688 | 125,698 | *rps15* | SSC |
|  | p1 | T | 11 | 126,488 | 126,498 | *ycf1* | SSC |
|  | p1 | T | 11 | 128,898 | 128,908 | *ycf1* | SSC |
| 5 | p1 | T | 12 | 8,312 | 8,323 | IGS (*psbK – psbI*) | LSC |
|  | p1 | T | 12 | 52,396 | 52,407 | IGS (*ndhK – ndhC*) | LSC |
|  | p1 | T | 12 | 67,101 | 67,112 | IGS (*psbE – petL*) | LSC |
|  | p1 | T | 12 | 69,011 | 69,022 | IGS (*psaJ – rpl33*) | LSC |
|  | p1 | T | 12 | 115,713 | 115,724 | IGS (*rpl32 – trnL-UAG*) | SSC |
| 2 | p1 | T | 13 | 23,626 | 23,638 | *rpoC1* | LSC |
|  | p1 | T | 13 | 132,843 | 132,855 | IGS (*trnR-ACG – rrn5*) | IRa |
| 2 | p1 | T | 14 | 19,202 | 19,215 | *rpoC2* | LSC |
|  | p1 | T | 14 | 84,594 | 84,607 | IGS (*rpl16 – rps3*) | LSC |
| 1 | p1 | T | 15 | 79,724 | 79,738 | IGS (*petD – rpoA*) | LSC |
| 1 | p4 | (TAAA)5 | 20 | 72,550 | 72,569 | *clpP* | LSC |
| 1 | c | (A)10 ... (T)10 | 41 | 123,537 | 123,577 | *ndhA* | SSC |
| 1 | c | (A)16 ... (A)11 | 75 | 8,868 | 8,942 | IGS (*trnS-GCU – trnG-UCC*) | LSC |
| 1 | c | (T)10 ... (A)10 | 28 | 12,520 | 12,547 | IGS (*atpA – atpF*) | LSC |
| 1 | c | (T)10 ... (A)14 | 59 | 65,765 | 65,823 | IGS (*petA – psbJ*) | LSC |
| 1 | c | (T)10 ... (T)10 | 56 | 1,720 | 1,775 | IGS (*psbA – trnK-UUU*) | LSC |
| 1 | c | (T)10 ... (T)11 | 99 | 50,868 | 50,966 | IGS (*trnF-GAA – ndhJ*) | LSC |
| 1 | c | (T)10 ... (T)18 | 109 | 72,191 | 72,299 | *clpP* | LSC |
| 1 | c | (T)11 ... (A)10 | 29 | 122,820 | 122,848 | *ndhA* | SSC |
| *Acer negundo* | | | | | | |  |
| 19 | p1 | A | 10 | 374 | 383 | IGS (*trnH-GUG – psbA*) | LSC |
|  | p1 | A | 10 | 5,716 | 5,725 | *rps16* | LSC |
|  | p1 | A | 10 | 7,148 | 7,157 | IGS (*rps16 – trnQ-UUG*) | LSC |
|  | p1 | A | 10 | 31,798 | 31,807 | IGS (*trnE-UUC – trnT-GGU*) | LSC |
|  | p1 | A | 10 | 48,787 | 48,796 | IGS (*trnT-UGU – trnL-UAA*) | LSC |
|  | p1 | A | 10 | 48,918 | 48,927 | IGS (*trnT-UGU – trnL-UAA*) | LSC |
|  | p1 | A | 10 | 52,937 | 52,946 | IGS (*ndhC – trnV-UAC*) | LSC |
|  | p1 | A | 10 | 57,108 | 57,117 | IGS (*atpB – rbcL*) | LSC |
|  | p1 | A | 10 | 61,655 | 61,664 | *psaI* | LSC |
|  | p1 | A | 10 | 61,844 | 61,853 | IGS (*psaI – ycf4*) | LSC |
|  | p1 | A | 10 | 66,321 | 66,330 | *psbF* | LSC |
|  | p1 | A | 10 | 72,648 | 72,657 | *clpP* | LSC |
|  | p1 | A | 10 | 78,397 | 78,406 | *petD* | LSC |
|  | p1 | A | 10 | 111,730 | 111,739 | *ycf1*; *ndhF* | IRb |
|  | p1 | A | 10 | 114,303 | 114,312 | IGS (*ndhF – rpl32*) | SSC |
|  | p1 | A | 10 | 115,732 | 115,741 | IGS (*rpl32 – trnL-UAG*) | SSC |
|  | p1 | A | 10 | 125,451 | 125,460 | IGS (*rps15 – ycf1*) | SSC |
|  | p1 | A | 10 | 127,822 | 127,831 | *ycf1* | SSC |
|  | p1 | A | 10 | 130,041 | 130,050 | *ycf1* | IRa |
| 6 | p1 | A | 11 | 6,361 | 6,371 | *rps16* | LSC |
|  | p1 | A | 11 | 8,915 | 8,925 | IGS (*trnS-GCU – trnG-UCC*) | LSC |
|  | p1 | A | 11 | 109,238 | 109,248 | IGS (*rrn5 – trnR-ACG*) | IRb |
|  | p1 | A | 11 | 114,659 | 114,669 | IGS (*ndhF – rpl32*) | SSC |
|  | p1 | A | 11 | 129,045 | 129,055 | *ycf1* | SSC |
|  | p1 | A | 11 | 129,699 | 129,709 | *ycf1* | SSC |
| 2 | p1 | A | 12 | 10,529 | 10,540 | IGS (*trnG-UCC – trnR-UCU*) | LSC |
|  | p1 | A | 12 | 115,091 | 115,102 | IGS (*rpl32 – trnL-UAG*) | SSC |
| 2 | p1 | A | 14 | 8,223 | 8,236 | IGS (*psbK – psbI*) | LSC |
|  | p1 | A | 14 | 53,351 | 53,364 | IGS (*ndhC – trnV-UAC*) | LSC |
| 1 | p1 | A | 15 | 82,547 | 82,561 | IGS (*rpl14 – rpl16*) | LSC |
| 1 | p1 | C | 10 | 81,243 | 81,252 | *infA* | LSC |
| 18 | p1 | T | 10 | 1,727 | 1,736 | IGS (*psbA – trnK-UUU*) | LSC |
|  | p1 | T | 10 | 9,411 | 9,420 | IGS (*trnS-GCU – trnG-UCC*) | LSC |
|  | p1 | T | 10 | 12,444 | 12,453 | IGS (*atpA – atpF*) | LSC |
|  | p1 | T | 10 | 26,800 | 26,809 | *rpoB* | LSC |
|  | p1 | T | 10 | 44,696 | 44,705 | *ycf3* | LSC |
|  | p1 | T | 10 | 48,491 | 48,500 | IGS (*trnT-UGU – trnL-UAA*) | LSC |
|  | p1 | T | 10 | 50,820 | 50,829 | IGS (*trnF-GAA – ndhJ*) | LSC |
|  | p1 | T | 10 | 53,664 | 53,673 | *trnV-UAC* | LSC |
|  | p1 | T | 10 | 56,542 | 56,551 | *atpB* | LSC |
|  | p1 | T | 10 | 62,832 | 62,841 | IGS (*ycf4 – cemA*) | LSC |
|  | p1 | T | 10 | 66,961 | 66,970 | IGS (*psbE – petL*) | LSC |
|  | p1 | T | 10 | 69,435 | 69,444 | IGS (*rpl33 – rps18*) | LSC |
|  | p1 | T | 10 | 75,545 | 75,554 | IGS (*psbB – psbT*) | LSC |
|  | p1 | T | 10 | 85,669 | 85,678 | *rps19* | LSC |
|  | p1 | T | 10 | 111,567 | 111,576 | *ycf1* | IRb |
|  | p1 | T | 10 | 115,352 | 115,361 | IGS (*rpl32 – trnL-UAG*) | SSC |
|  | p1 | T | 10 | 126,684 | 126,693 | *ycf1* | SSC |
|  | p1 | T | 10 | 129,878 | 129,887 | *ycf1* | IRa |
| 9 | p1 | T | 11 | 23,556 | 23,566 | *rpoC1* | LSC |
|  | p1 | T | 11 | 30,031 | 30,041 | IGS (*petN – psbM*) | LSC |
|  | p1 | T | 11 | 62,047 | 62,057 | IGS (*psaI – ycf4*) | LSC |
|  | p1 | T | 11 | 72,977 | 72,987 | *clpP* | LSC |
|  | p1 | T | 11 | 115,237 | 115,247 | IGS (*rpl32 – trnL-UAG*) | SSC |
|  | p1 | T | 11 | 125,198 | 125,208 | *rps15* | SSC |
|  | p1 | T | 11 | 125,999 | 126,009 | *ycf1* | SSC |
|  | p1 | T | 11 | 128,406 | 128,416 | *ycf1* | SSC |
|  | p1 | T | 11 | 132,369 | 132,379 | IGS (*trnR-ACG – rrn5*) | IRa |
| 3 | p1 | T | 12 | 14,888 | 14,899 | IGS (*atpH – atpI*) | LSC |
|  | p1 | T | 12 | 28,270 | 28,281 | IGS (*rpoB – trnC-GCA*) | LSC |
|  | p1 | T | 12 | 123,081 | 123,092 | *ndhA* | SSC |
| 1 | p1 | T | 13 | 71,986 | 71,998 | *clpP* | LSC |
| 1 | p1 | T | 14 | 19,133 | 19,146 | *rpoC2* | LSC |
| 2 | p1 | T | 15 | 30,213 | 30,227 | IGS (*psbM – trnD-GUC*) | LSC |
|  | p1 | T | 15 | 52,259 | 52,273 | IGS (*ndhK – ndhC*) | LSC |
| 1 | c | (A)10 (AAT)5 | 23 | 37,324 | 37,346 | IGS (*psbZ – trnG-GCC*) | LSC |
| 1 | c | (A)11 ... (A)10 ... (A)10 | 186 | 4,778 | 4,963 | IGS (*trnK-UUU – rps16*) | LSC |
| 1 | c | (T)12 ... (C)10 | 100 | 56,897 | 56,996 | IGS (*atpB – rbcL*) | LSC |
| *Acer nipponicum* | | | | | | |  |
| 11 | p1 | A | 10 | 6,296 | 6,305 | *rps16* | LSC |
|  | p1 | A | 10 | 10,394 | 10,403 | IGS (*trnG-UCC – trnR-UCU*) | LSC |
|  | p1 | A | 10 | 43,626 | 43,635 | IGS (*psaA – ycf3*) | LSC |
|  | p1 | A | 10 | 46,534 | 46,543 | IGS (*ycf3 – trnS-GGA*) | LSC |
|  | p1 | A | 10 | 61,530 | 61,539 | *psaI* | LSC |
|  | p1 | A | 10 | 65,517 | 65,526 | IGS (*petA – psbJ*) | LSC |
|  | p1 | A | 10 | 66,172 | 66,181 | *psbF* | LSC |
|  | p1 | A | 10 | 111,870 | 111,879 | *ycf1*; *ndhF* | IRb |
|  | p1 | A | 10 | 119,204 | 119,213 | IGS (*ndhD – psaC*) | SSC |
|  | p1 | A | 10 | 125,749 | 125,758 | IGS (*rps15 – ycf1*) | SSC |
|  | p1 | A | 10 | 130,333 | 130,342 | *ycf1* | IRa |
| 7 | p1 | A | 11 | 43,799 | 43,809 | IGS (*psaA – ycf3*) | LSC |
|  | p1 | A | 11 | 52,933 | 52,943 | IGS (*ndhC – trnV-UAC*) | LSC |
|  | p1 | A | 11 | 109,379 | 109,389 | IGS (*rrn5 – trnR-ACG*) | IRb |
|  | p1 | A | 11 | 128,114 | 128,124 | *ycf1* | SSC |
|  | p1 | A | 11 | 129,337 | 129,347 | *ycf1* | SSC |
|  | p1 | A | 11 | 129,991 | 130,001 | *ycf1* | SSC |
|  | p1 | A | 11 | 132,246 | 132,256 | IGS (*trnN-GUU – trnR-ACG*) | IRa |
| 5 | p1 | A | 12 | 407 | 418 | IGS (*trnH-GUG – psbA*) | LSC |
|  | p1 | A | 12 | 5,643 | 5,654 | *rps16* | LSC |
|  | p1 | A | 12 | 48,903 | 48,914 | IGS (*trnT-UGU – trnL-UAA*) | LSC |
|  | p1 | A | 12 | 78,446 | 78,457 | *petD* | LSC |
|  | p1 | A | 12 | 116,137 | 116,148 | IGS (*rpl32 – trnL-UAG*) | SSC |
| 1 | p1 | A | 13 | 13,252 | 13,264 | *atpF* | LSC |
| 2 | p1 | A | 14 | 4,878 | 4,891 | IGS (*trnK-UUU – rps16*) | LSC |
|  | p1 | A | 14 | 8,667 | 8,680 | IGS (*trnS-GCU – trnG-UCC*) | LSC |
| 3 | p1 | A | 15 | 15,146 | 15,160 | IGS (*atpH – atpI*) | LSC |
|  | p1 | A | 15 | 77,012 | 77,026 | *petB* | LSC |
|  | p1 | A | 15 | 114,887 | 114,901 | IGS (*ndhF – rpl32*) | SSC |
| 1 | p1 | A | 16 | 156,204 | 156,219 | IGS (*rpl2 – trnH-GUG*) | IRa |
| 1 | p1 | C | 10 | 81,378 | 81,387 | *infA* | LSC |
| 17 | p1 | T | 10 | 12,358 | 12,367 | IGS (*atpA – atpF*) | LSC |
|  | p1 | T | 10 | 12,949 | 12,958 | *atpF* | LSC |
|  | p1 | T | 10 | 23,484 | 23,493 | *rpoC1* | LSC |
|  | p1 | T | 10 | 26,727 | 26,736 | *rpoB* | LSC |
|  | p1 | T | 10 | 56,557 | 56,566 | *atpB* | LSC |
|  | p1 | T | 10 | 56,823 | 56,832 | IGS (*atpB – rbcL*) | LSC |
|  | p1 | T | 10 | 61,893 | 61,902 | IGS (*psaI – ycf4*) | LSC |
|  | p1 | T | 10 | 62,688 | 62,697 | IGS (*ycf4 – cemA*) | LSC |
|  | p1 | T | 10 | 68,718 | 68,727 | IGS (*psaJ – rpl33*) | LSC |
|  | p1 | T | 10 | 69,454 | 69,463 | IGS (*rpl33 – rps18*) | LSC |
|  | p1 | T | 10 | 72,979 | 72,988 | *clpP* | LSC |
|  | p1 | T | 10 | 75,556 | 75,565 | IGS (*psbB – psbT*) | LSC |
|  | p1 | T | 10 | 82,152 | 82,161 | IGS (*rps8 – rpl14*) | LSC |
|  | p1 | T | 10 | 111,707 | 111,716 | *ycf1* | IRb |
|  | p1 | T | 10 | 123,059 | 123,068 | *ndhA* | SSC |
|  | p1 | T | 10 | 128,824 | 128,833 | *ycf1* | SSC |
|  | p1 | T | 10 | 130,170 | 130,179 | *ycf1* | IRa |
| 13 | p1 | T | 11 | 8,949 | 8,959 | IGS (*trnS-GCU – trnG-UCC*) | LSC |
|  | p1 | T | 11 | 44,668 | 44,678 | *ycf3* | LSC |
|  | p1 | T | 11 | 53,675 | 53,685 | *trnV-UAC* | LSC |
|  | p1 | T | 11 | 66,812 | 66,822 | IGS (*psbE – petL*) | LSC |
|  | p1 | T | 11 | 79,423 | 79,433 | IGS (*petD – rpoA*) | LSC |
|  | p1 | T | 11 | 109,793 | 109,803 | IGS (*trnR-ACG – trnN-GUU*) | IRb |
|  | p1 | T | 11 | 115,488 | 115,498 | IGS (*rpl32 – trnL-UAG*) | SSC |
|  | p1 | T | 11 | 115,603 | 115,613 | IGS (*rpl32 – trnL-UAG*) | SSC |
|  | p1 | T | 11 | 125,251 | 125,261 | IGS (*ndhH – rps15*) | SSC |
|  | p1 | T | 11 | 125,490 | 125,500 | *rps15* | SSC |
|  | p1 | T | 11 | 126,288 | 126,298 | *ycf1* | SSC |
|  | p1 | T | 11 | 128,698 | 128,708 | *ycf1* | SSC |
|  | p1 | T | 11 | 132,660 | 132,670 | IGS (*trnR-ACG – rrn5*) | IRa |
| 1 | p1 | T | 12 | 49,875 | 49,886 | IGS (*trnL-UAA – trnF-GAA*) | LSC |
| 2 | p1 | T | 13 | 19,070 | 19,082 | *rpoC2* | LSC |
|  | p1 | T | 13 | 30,154 | 30,166 | IGS (*psbM – trnD-GUC*) | LSC |
| 1 | p1 | T | 14 | 52,256 | 52,269 | IGS (*ndhK – ndhC*) | LSC |
| 1 | p1 | T | 16 | 85,830 | 85,845 | *rps19* | IRb |
| 2 | p2 | (TA)6 | 12 | 32,187 | 32,198 | IGS (*trnE-UUC – trnT-GGU*) | LSC |
|  | p2 | (TA)6 | 12 | 78,685 | 78,696 | *petD* | LSC |
| 1 | c | (A)11 (AAT)5 | 24 | 37,269 | 37,292 | IGS (*psbZ – trnG-GCC*) | LSC |
| 1 | c | (A)10 ... (A)13 | 120 | 115,234 | 115,353 | IGS (*rpl32 – trnL-UAG*) | SSC |
| 1 | c | (A)10 ... (T)12 | 43 | 123,341 | 123,383 | *ndhA* | SSC |
| 2 | c | (T)10 ... (T)11 | 74 | 9,101 | 9,174 | IGS (*trnS-GCU – trnG-UCC*) | LSC |
|  | c | (T)10 ... (T)11 | 102 | 71,893 | 71,994 | *clpP* | LSC |
| 1 | c | (T)11 ... (A)14 | 73 | 82,643 | 82,715 | IGS (*rpl14 – rpl16*) | LSC |
| 1 | c | (T)11 ... (T)11 | 72 | 1,758 | 1,829 | IGS (*psbA – trnK-UUU*) | LSC |
| *Acer oblongum* | | | | | | |  |
| 9 | p1 | A | 10 | 3,640 | 3,649 | *trnK-UUU* | LSC |
|  | p1 | A | 10 | 37,799 | 37,808 | IGS (*trnG-GCC – trnfM-CAU*) | LSC |
|  | p1 | A | 10 | 46,381 | 46,390 | IGS (*ycf3 – trnS-GGA*) | LSC |
|  | p1 | A | 10 | 56,876 | 56,885 | IGS (*atpB – rbcL*) | LSC |
|  | p1 | A | 10 | 58,931 | 58,940 | IGS (*rbcL – accD*) | LSC |
|  | p1 | A | 10 | 65,442 | 65,451 | IGS (*petA – psbJ*) | LSC |
|  | p1 | A | 10 | 66,078 | 66,087 | *psbF* | LSC |
|  | p1 | A | 10 | 111,738 | 111,747 | *ycf1*; *ndhF* | IRb |
|  | p1 | A | 10 | 129,768 | 129,777 | *ycf1* | IRa |
| 7 | p1 | A | 11 | 8,789 | 8,799 | IGS (*trnS-GCU – trnG-UCC*) | LSC |
|  | p1 | A | 11 | 15,117 | 15,127 | IGS (*atpH – atpI*) | LSC |
|  | p1 | A | 11 | 52,693 | 52,703 | IGS (*ndhC – trnV-UAC*) | LSC |
|  | p1 | A | 11 | 78,319 | 78,329 | *petD* | LSC |
|  | p1 | A | 11 | 127,561 | 127,571 | *ycf1* | SSC |
|  | p1 | A | 11 | 128,772 | 128,782 | *ycf1* | SSC |
|  | p1 | A | 11 | 129,426 | 129,436 | *ycf1* | SSC |
| 4 | p1 | A | 12 | 5,620 | 5,631 | *rps16* | LSC |
|  | p1 | A | 12 | 13,229 | 13,240 | *atpF* | LSC |
|  | p1 | A | 12 | 76,871 | 76,882 | *petB* | LSC |
|  | p1 | A | 12 | 109,257 | 109,268 | IGS (*rrn5 – trnR-ACG*) | IRb |
| 1 | p1 | A | 13 | 73,706 | 73,718 | IGS (*clpP – psbB*) | LSC |
| 1 | p1 | A | 14 | 114,643 | 114,656 | IGS (*ndhF – rpl32*) | SSC |
| 2 | p1 | A | 15 | 48,730 | 48,744 | IGS (*trnT-UGU – trnL-UAA*) | LSC |
|  | p1 | A | 15 | 82,555 | 82,569 | IGS (*rpl14 – rpl16*) | LSC |
| 1 | p1 | A | 18 | 53,118 | 53,135 | IGS (*ndhC – trnV-UAC*) | LSC |
| 2 | p1 | C | 10 | 8,405 | 8,414 | IGS (*psbK – psbI*) | LSC |
|  | p1 | C | 10 | 50,158 | 50,167 | IGS (*trnF-GAA – ndhJ*) | LSC |
| 1 | p1 | C | 14 | 81,239 | 81,252 | *infA* | LSC |
| 11 | p1 | T | 10 | 8,971 | 8,980 | IGS (*trnS-GCU – trnG-UCC*) | LSC |
|  | p1 | T | 10 | 26,691 | 26,700 | *rpoB* | LSC |
|  | p1 | T | 10 | 44,519 | 44,528 | *ycf3* | LSC |
|  | p1 | T | 10 | 56,317 | 56,326 | *atpB* | LSC |
|  | p1 | T | 10 | 69,353 | 69,362 | IGS (*rpl33 – rps18*) | LSC |
|  | p1 | T | 10 | 111,575 | 111,584 | *ycf1* | IRb |
|  | p1 | T | 10 | 118,643 | 118,652 | IGS (*ndhD – psaC*) | SSC |
|  | p1 | T | 10 | 122,245 | 122,254 | *ndhA* | SSC |
|  | p1 | T | 10 | 128,134 | 128,143 | *ycf1* | SSC |
|  | p1 | T | 10 | 128,259 | 128,268 | *ycf1* | SSC |
|  | p1 | T | 10 | 129,605 | 129,614 | *ycf1* | IRa |
| 6 | p1 | T | 11 | 49,712 | 49,722 | IGS (*trnL-UAA – trnF-GAA*) | LSC |
|  | p1 | T | 11 | 53,428 | 53,438 | *trnV-UAC* | LSC |
|  | p1 | T | 11 | 66,718 | 66,728 | IGS (*psbE – petL*) | LSC |
|  | p1 | T | 11 | 68,622 | 68,632 | IGS (*psaJ – rpl33*) | LSC |
|  | p1 | T | 11 | 122,527 | 122,537 | *ndhA* | SSC |
|  | p1 | T | 11 | 124,953 | 124,963 | *rps15* | SSC |
| 2 | p1 | T | 12 | 30,114 | 30,125 | IGS (*psbM – trnD-GUC*) | LSC |
|  | p1 | T | 12 | 132,084 | 132,095 | IGS (*trnR-ACG – rrn5*) | IRa |
| 3 | p1 | T | 13 | 19,034 | 19,046 | *rpoC2* | LSC |
|  | p1 | T | 13 | 52,002 | 52,014 | IGS (*ndhK – ndhC*) | LSC |
|  | p1 | T | 13 | 115,002 | 115,014 | IGS (*rpl32 – trnL-UAG*) | SSC |
| 1 | p1 | T | 14 | 72,850 | 72,863 | *clpP* | LSC |
| 1 | p1 | T | 16 | 71,857 | 71,872 | *clpP* | LSC |
| 2 | p2 | (AT)6 | 12 | 10,554 | 10,565 | IGS (*trnR-UCU – atpA*) | LSC |
|  | p2 | (AT)6 | 12 | 32,108 | 32,119 | IGS (*trnE-UUC – trnT-GGU*) | LSC |
| 1 | p3 | (TAT)6 | 18 | 48,268 | 48,285 | IGS (*trnT-UGU – trnL-UAA*) | LSC |
| 1 | c | (A)10 (AAT)5 | 23 | 37,179 | 37,201 | IGS (*psbZ – trnG-GCC*) | LSC |
| 1 | c | (A)10 ... (T)12 | 58 | 61,399 | 61,456 | *psaI* | LSC |
| 1 | c | (A)11 ... (A)15 | 118 | 4,763 | 4,880 | IGS (*trnK-UUU – rps16*) | LSC |
| 1 | c | (T)10 ... (A)12 | 41 | 16,794 | 16,834 | IGS (*rps2 – rpoC2*) | LSC |
| 1 | c | (T)10 ... (T)10 | 45 | 23,448 | 23,492 | *rpoC1* | LSC |
| *Acer palmatum* var. *palmatum* | | | | | | |  |
| 10 | p1 | A | 10 | 417 | 426 | IGS (*trnH-GUG – psbA*) | LSC |
|  | p1 | A | 10 | 52,981 | 52,990 | IGS (*ndhC – trnV-UAC*) | LSC |
|  | p1 | A | 10 | 53,379 | 53,388 | IGS (*ndhC – trnV-UAC*) | LSC |
|  | p1 | A | 10 | 66,369 | 66,378 | *psbF* | LSC |
|  | p1 | A | 10 | 77,241 | 77,250 | *petB* | LSC |
|  | p1 | A | 10 | 78,653 | 78,662 | *petD* | LSC |
|  | p1 | A | 10 | 112,067 | 112,076 | *ycf1*; *ndhF* | IRb |
|  | p1 | A | 10 | 125,863 | 125,872 | IGS (*rps15 – ycf1*) | SSC |
|  | p1 | A | 10 | 128,234 | 128,243 | *ycf1* | SSC |
|  | p1 | A | 10 | 130,453 | 130,462 | *ycf1* | IRa |
| 8 | p1 | A | 11 | 8,219 | 8,229 | IGS (*psbK – psbI*) | LSC |
|  | p1 | A | 11 | 8,907 | 8,917 | IGS (*trnS-GCU – trnG-UCC*) | LSC |
|  | p1 | A | 11 | 13,435 | 13,445 | *atpF* | LSC |
|  | p1 | A | 11 | 15,321 | 15,331 | IGS (*atpH – atpI*) | LSC |
|  | p1 | A | 11 | 37,424 | 37,434 | IGS (*psbZ – trnG-GCC*) | LSC |
|  | p1 | A | 11 | 43,706 | 43,716 | IGS (*psaA – ycf3*) | LSC |
|  | p1 | A | 11 | 129,457 | 129,467 | *ycf1* | SSC |
|  | p1 | A | 11 | 130,111 | 130,121 | *ycf1* | SSC |
| 3 | p1 | A | 12 | 48,969 | 48,980 | IGS (*trnT-UGU – trnL-UAA*) | LSC |
|  | p1 | A | 12 | 74,064 | 74,075 | IGS (*clpP – psbB*) | LSC |
|  | p1 | A | 12 | 114,631 | 114,642 | IGS (*ndhF – rpl32*) | SSC |
| 3 | p1 | A | 13 | 65,730 | 65,742 | IGS (*petA – psbJ*) | LSC |
|  | p1 | A | 13 | 109,567 | 109,579 | IGS (*rrn5 – trnR-ACG*) | IRb |
|  | p1 | A | 13 | 114,982 | 114,994 | IGS (*ndhF – rpl32*) | SSC |
| 1 | p1 | A | 16 | 82,888 | 82,903 | IGS (*rpl14 – rpl16*) | LSC |
| 1 | p1 | C | 13 | 50,377 | 50,389 | IGS (*trnF-GAA – ndhJ*) | LSC |
| 10 | p1 | T | 10 | 9,596 | 9,605 | IGS (*trnS-GCU – trnG-UCC*) | LSC |
|  | p1 | T | 10 | 26,866 | 26,875 | *rpoB* | LSC |
|  | p1 | T | 10 | 32,552 | 32,561 | IGS (*trnT-GGU – psbD*) | LSC |
|  | p1 | T | 10 | 38,018 | 38,027 | IGS (*trnG-GCC – trnfM-CAU*) | LSC |
|  | p1 | T | 10 | 44,750 | 44,759 | *ycf3* | LSC |
|  | p1 | T | 10 | 50,864 | 50,873 | IGS (*trnF-GAA – ndhJ*) | LSC |
|  | p1 | T | 10 | 56,606 | 56,615 | *atpB* | LSC |
|  | p1 | T | 10 | 69,665 | 69,674 | IGS (*rpl33 – rps18*) | LSC |
|  | p1 | T | 10 | 111,904 | 111,913 | *ycf1* | IRb |
|  | p1 | T | 10 | 130,290 | 130,299 | *ycf1* | IRa |
| 9 | p1 | T | 11 | 8,347 | 8,357 | IGS (*psbK – psbI*) | LSC |
|  | p1 | T | 11 | 12,531 | 12,541 | IGS (*atpA – atpF*) | LSC |
|  | p1 | T | 11 | 13,122 | 13,132 | *atpF* | LSC |
|  | p1 | T | 11 | 48,532 | 48,542 | IGS (*trnT-UGU – trnL-UAA*) | LSC |
|  | p1 | T | 11 | 62,883 | 62,893 | IGS (*ycf4 – cemA*) | LSC |
|  | p1 | T | 11 | 73,211 | 73,221 | *clpP* | LSC |
|  | p1 | T | 11 | 125,611 | 125,621 | *rps15* | SSC |
|  | p1 | T | 11 | 126,411 | 126,421 | *ycf1* | SSC |
|  | p1 | T | 11 | 128,818 | 128,828 | *ycf1* | SSC |
| 2 | p1 | T | 12 | 68,928 | 68,939 | IGS (*psaJ – rpl33*) | LSC |
|  | p1 | T | 12 | 119,306 | 119,317 | IGS (*ndhD – psaC*) | SSC |
| 4 | p1 | T | 13 | 14,992 | 15,004 | IGS (*atpH – atpI*) | LSC |
|  | p1 | T | 13 | 79,620 | 79,632 | IGS (*petD – rpoA*) | LSC |
|  | p1 | T | 13 | 115,602 | 115,614 | IGS (*rpl32 – trnL-UAG*) | SSC |
|  | p1 | T | 13 | 132,787 | 132,799 | IGS (*trnR-ACG – rrn5*) | IRa |
| 2 | p1 | T | 14 | 19,231 | 19,244 | *rpoC2* | LSC |
|  | p1 | T | 14 | 30,298 | 30,311 | IGS (*psbM – trnD-GUC*) | LSC |
| 1 | p1 | T | 15 | 52,303 | 52,317 | IGS (*ndhK – ndhC*) | LSC |
| 1 | p2 | (AT)7 | 14 | 32,342 | 32,355 | IGS (*trnE-UUC – trnT-GGU*) | LSC |
| 1 | p2 | (AT)8 | 16 | 78,879 | 78,894 | *petD* | LSC |
| 1 | c | (A)10 ... (A)14 | 119 | 115,327 | 115,445 | IGS (*rpl32 – trnL-UAG*) | SSC |
| 1 | c | (A)10 ... (T)13 | 59 | 61,706 | 61,764 | *psaI* | LSC |
| 1 | c | (A)12 ... (A)11 ... (A)11 | 182 | 4,792 | 4,973 | IGS (*trnK-UUU – rps16*) | LSC |
| 1 | c | (A)13 ... (T)12 | 46 | 123,460 | 123,505 | *ndhA* | SSC |
| 1 | c | (T)10 ... (A)13 | 31 | 122,762 | 122,792 | *ndhA* | SSC |
| 1 | c | (T)10 ... (T)10 | 29 | 54,414 | 54,442 | IGS (*trnV-UAC – trnM-CAU*) | LSC |
| 1 | c | (T)11 ... (T)11 | 103 | 72,124 | 72,226 | *clpP* | LSC |
| *Acer pentaphyllum* | | | | | | |  |
| 9 | p1 | A | 10 | 5,743 | 5,752 | *rps16* | LSC |
|  | p1 | A | 10 | 38,034 | 38,043 | IGS (*trnG-GCC – trnfM-CAU*) | LSC |
|  | p1 | A | 10 | 43,705 | 43,714 | IGS (*psaA – ycf3*) | LSC |
|  | p1 | A | 10 | 52,986 | 52,995 | IGS (*ndhC – trnV-UAC*) | LSC |
|  | p1 | A | 10 | 61,709 | 61,718 | *psaI* | LSC |
|  | p1 | A | 10 | 66,321 | 66,330 | *psbF* | LSC |
|  | p1 | A | 10 | 111,978 | 111,987 | *ycf1*; *ndhF* | IRb |
|  | p1 | A | 10 | 130,335 | 130,344 | *ycf1* | IRa |
|  | p1 | A | 10 | 132,248 | 132,257 | IGS (*trnN-GUU – trnR-ACG*) | IRa |
| 8 | p1 | A | 11 | 406 | 416 | IGS (*trnH-GUG – psbA*) | LSC |
|  | p1 | A | 11 | 3,733 | 3,743 | *trnK-UUU* | LSC |
|  | p1 | A | 11 | 57,171 | 57,181 | IGS (*atpB – rbcL*) | LSC |
|  | p1 | A | 11 | 109,497 | 109,507 | IGS (*rrn5 – trnR-ACG*) | IRb |
|  | p1 | A | 11 | 115,357 | 115,367 | IGS (*rpl32 – trnL-UAG*) | SSC |
|  | p1 | A | 11 | 128,128 | 128,138 | *ycf1* | SSC |
|  | p1 | A | 11 | 129,339 | 129,349 | *ycf1* | SSC |
|  | p1 | A | 11 | 129,993 | 130,003 | *ycf1* | SSC |
| 3 | p1 | A | 12 | 48,947 | 48,958 | IGS (*trnT-UGU – trnL-UAA*) | LSC |
|  | p1 | A | 12 | 53,401 | 53,412 | IGS (*ndhC – trnV-UAC*) | LSC |
|  | p1 | A | 12 | 65,683 | 65,694 | IGS (*petA – psbJ*) | LSC |
| 1 | p1 | A | 13 | 8,912 | 8,924 | IGS (*trnS-GCU – trnG-UCC*) | LSC |
| 2 | p1 | A | 14 | 37,391 | 37,404 | IGS (*psbZ – trnG-GCC*) | LSC |
|  | p1 | A | 14 | 77,138 | 77,151 | *petB* | LSC |
| 1 | p1 | A | 15 | 15,309 | 15,323 | IGS (*atpH – atpI*) | LSC |
| 1 | p1 | A | 16 | 114,905 | 114,920 | IGS (*ndhF – rpl32*) | SSC |
| 1 | p1 | C | 14 | 50,377 | 50,390 | IGS (*trnF-GAA – ndhJ*) | LSC |
| 14 | p1 | T | 10 | 9,337 | 9,346 | IGS (*trnS-GCU – trnG-UCC*) | LSC |
|  | p1 | T | 10 | 16,717 | 16,726 | *rps2* | LSC |
|  | p1 | T | 10 | 26,948 | 26,957 | *rpoB* | LSC |
|  | p1 | T | 10 | 44,748 | 44,757 | *ycf3* | LSC |
|  | p1 | T | 10 | 56,611 | 56,620 | *atpB* | LSC |
|  | p1 | T | 10 | 56,966 | 56,975 | IGS (*atpB – rbcL*) | LSC |
|  | p1 | T | 10 | 66,961 | 66,970 | IGS (*psbE – petL*) | LSC |
|  | p1 | T | 10 | 78,833 | 78,842 | *petD* | LSC |
|  | p1 | T | 10 | 85,929 | 85,938 | *rps19* | LSC |
|  | p1 | T | 10 | 109,902 | 109,911 | IGS (*trnR-ACG – trnN-GUU*) | IRb |
|  | p1 | T | 10 | 111,815 | 111,824 | *ycf1* | IRb |
|  | p1 | T | 10 | 115,502 | 115,511 | IGS (*rpl32 – trnL-UAG*) | SSC |
|  | p1 | T | 10 | 126,963 | 126,972 | *ycf1* | SSC |
|  | p1 | T | 10 | 130,172 | 130,181 | *ycf1* | IRa |
| 9 | p1 | T | 11 | 14,965 | 14,975 | IGS (*atpH – atpI*) | LSC |
|  | p1 | T | 11 | 52,312 | 52,322 | IGS (*ndhK – ndhC*) | LSC |
|  | p1 | T | 11 | 54,437 | 54,447 | IGS (*trnV-UAC – trnM-CAU*) | LSC |
|  | p1 | T | 11 | 68,853 | 68,863 | IGS (*psaJ – rpl33*) | LSC |
|  | p1 | T | 11 | 119,150 | 119,160 | IGS (*ndhD – psaC*) | SSC |
|  | p1 | T | 11 | 125,493 | 125,503 | *rps15* | SSC |
|  | p1 | T | 11 | 126,284 | 126,294 | *ycf1* | SSC |
|  | p1 | T | 11 | 128,700 | 128,710 | *ycf1* | SSC |
|  | p1 | T | 11 | 132,652 | 132,662 | IGS (*trnR-ACG – rrn5*) | IRa |
| 3 | p1 | T | 12 | 30,396 | 30,407 | IGS (*psbM – trnD-GUC*) | LSC |
|  | p1 | T | 12 | 50,871 | 50,882 | IGS (*trnF-GAA – ndhJ*) | LSC |
|  | p1 | T | 12 | 53,719 | 53,730 | *trnV-UAC* | LSC |
| 1 | p1 | T | 13 | 19,233 | 19,245 | *rpoC2* | LSC |
| 1 | p1 | T | 16 | 73,078 | 73,093 | *clpP* | LSC |
| 2 | p2 | (AT)6 | 12 | 10,768 | 10,779 | IGS (*trnR-UCU – atpA*) | LSC |
|  | p2 | (AT)6 | 12 | 32,390 | 32,401 | IGS (*trnE-UUC – trnT-GGU*) | LSC |
| 1 | c | (A)10 ... (A)10 ... (A)17 | 185 | 4,806 | 4,990 | IGS (*trnK-UUU – rps16*) | LSC |
| 1 | c | (A)10 ... (T)13 | 44 | 123,345 | 123,388 | *ndhA* | SSC |
| 1 | c | (T)10 ... (T)11 | 46 | 23,704 | 23,749 | *rpoC1* | LSC |
| 1 | c | (T)10 ... (T)13 | 104 | 72,003 | 72,106 | *clpP* | LSC |
| 1 | c | (T)11 ... (A)13 | 63 | 82,756 | 82,818 | IGS (*rpl14 – rpl16*) | LSC |
| *Acer pilosum* | | | | | | |  |
| 13 | p1 | A | 10 | 255 | 264 | IGS (*trnH-GUG – psbA*) | LSC |
|  | p1 | A | 10 | 4,601 | 4,610 | IGS (*trnK-UUU – rps16*) | LSC |
|  | p1 | A | 10 | 7,842 | 7,851 | IGS (*psbK – psbI*) | LSC |
|  | p1 | A | 10 | 36,993 | 37,002 | IGS (*psbZ – trnG-GCC*) | LSC |
|  | p1 | A | 10 | 46,194 | 46,203 | IGS (*ycf3 – trnS-GGA*) | LSC |
|  | p1 | A | 10 | 52,817 | 52,826 | IGS (*ndhC – trnV-UAC*) | LSC |
|  | p1 | A | 10 | 56,557 | 56,566 | IGS (*atpB – rbcL*) | LSC |
|  | p1 | A | 10 | 61,077 | 61,086 | *psaI* | LSC |
|  | p1 | A | 10 | 65,724 | 65,733 | *psbF* | LSC |
|  | p1 | A | 10 | 69,442 | 69,451 | IGS (*rps18 – rpl20*) | LSC |
|  | p1 | A | 10 | 83,691 | 83,700 | rpl16 | LSC |
|  | p1 | A | 10 | 113,913 | 113,922 | IGS (*ndhF – rpl32*) | SSC |
|  | p1 | A | 10 | 129,674 | 129,683 | ycf1 | IRa |
| 10 | p1 | A | 11 | 3,594 | 3,604 | *trnK-UUU* | LSC |
|  | p1 | A | 11 | 8,522 | 8,532 | IGS (*trnS-GCU – trnG-UCC*) | LSC |
|  | p1 | A | 11 | 14,875 | 14,885 | IGS (*atpH– atpI*) | LSC |
|  | p1 | A | 11 | 33,012 | 33,022 | IGS (*trnT-GGU – psbD*) | LSC |
|  | p1 | A | 11 | 37,610 | 37,620 | IGS (*trnG-GCC – trnfM-CAU*) | LSC |
|  | p1 | A | 11 | 52,401 | 52,411 | IGS (*ndhC – trnV-UAC*) | LSC |
|  | p1 | A | 11 | 65,083 | 65,093 | IGS (*petA – psbJ*) | LSC |
|  | p1 | A | 11 | 127,455 | 127,465 | *ycf1* | SSC |
|  | p1 | A | 11 | 128,678 | 128,688 | *ycf1* | SSC |
|  | p1 | A | 11 | 129,332 | 129,342 | *ycf1* | SSC |
| 1 | p1 | A | 12 | 82,184 | 82,195 | IGS (*rpl14 – rpl16*) | LSC |
| 2 | p1 | A | 13 | 48,383 | 48,395 | IGS (*trnT-UGU – trnL-UAA*) | LSC |
|  | p1 | A | 13 | 114,269 | 114,281 | IGS (*ndhF – rpl32*) | SSC |
| 1 | p1 | A | 14 | 108,888 | 108,901 | IGS (*rrn5 – trnR-ACG*) | IRb |
| 1 | p1 | C | 10 | 80,881 | 80,890 | *infA* | LSC |
| 1 | p1 | C | 11 | 49,802 | 49,812 | IGS (*trnF-GAA – ndhJ*) | LSC |
| 14 | p1 | T | 10 | 14,532 | 14,541 | IGS (*atpH – atpI*) | LSC |
|  | p1 | T | 10 | 26,444 | 26,453 | *rpoB* | LSC |
|  | p1 | T | 10 | 49,356 | 49,365 | IGS (*trnL-UAA – trnF-GAA*) | LSC |
|  | p1 | T | 10 | 53,126 | 53,135 | *trnV-UAC* | LSC |
|  | p1 | T | 10 | 55,995 | 56,004 | *atpB* | LSC |
|  | p1 | T | 10 | 68,990 | 68,999 | IGS (*rpl33 – rps18*) | LSC |
|  | p1 | T | 10 | 72,499 | 72,508 | *clpP* | LSC |
|  | p1 | T | 10 | 78,918 | 78,927 | IGS (*petD – rpoA*) | LSC |
|  | p1 | T | 10 | 111,217 | 111,226 | *ycf1* | IRb |
|  | p1 | T | 10 | 114,865 | 114,874 | IGS (*rpl32 – trnL-UAG*) | SSC |
|  | p1 | T | 10 | 114,979 | 114,988 | IGS (*rpl32 – trnL-UAG*) | SSC |
|  | p1 | T | 10 | 118,519 | 118,528 | IGS (*ndhD – psaC*) | SSC |
|  | p1 | T | 10 | 122,133 | 122,142 | *ndhA* | SSC |
|  | p1 | T | 10 | 129,511 | 129,520 | *ycf1* | SSC-IRa |
| 7 | p1 | T | 11 | 8,793 | 8,803 | IGS (*trnS-GCU – trnG-UCC*) | LSC |
|  | p1 | T | 11 | 12,655 | 12,665 | *atpF* | LSC |
|  | p1 | T | 11 | 23,200 | 23,210 | *rpoC1* | LSC |
|  | p1 | T | 11 | 56,350 | 56,360 | IGS (*atpB – rbcL*) | LSC |
|  | p1 | T | 11 | 124,838 | 124,848 | *rps15* | SSC |
|  | p1 | T | 11 | 125,638 | 125,648 | *ycf1* | SSC |
|  | p1 | T | 11 | 128,039 | 128,049 | *ycf1* | SSC |
| 1 | p1 | T | 12 | 51,726 | 51,737 | IGS (*ndhK – ndhC*) | LSC |
| 2 | p1 | T | 13 | 29,873 | 29,885 | IGS (*psbM – trnD-GUG*) | LSC |
|  | p1 | T | 13 | 50,284 | 50,296 | IGS (*trnF-GAA – ndhJ*) | LSC |
| 2 | p1 | T | 14 | 71,513 | 71,526 | *clpP* | LSC |
|  | p1 | T | 14 | 131,999 | 132,012 | IGS (*trnR-ACG – rrn5*) | IRa |
| 1 | p1 | T | 16 | 18,783 | 18,798 | *rpoC2* | LSC |
| 1 | c | (A)11 ... (A)11 | 117 | 114,614 | 114,730 | IGS (*rpl32 – trnL-UAG*) | SSC |
| 1 | c | (T)10 ... (T)24 | 70 | 1,595 | 1,664 | IGS (*psbA – trnK-UUU*) | LSC |
| *Acer platanoides* | | | | | | |  |
| 12 | p1 | A | 10 | 3,726 | 3,735 | *trnK-UUU* | LSC |
|  | p1 | A | 10 | 6,285 | 6,294 | *rps16* | LSC |
|  | p1 | A | 10 | 13,879 | 13,888 | IGS (*atpF – atpH*) | LSC |
|  | p1 | A | 10 | 37,409 | 37,418 | IGS (*psbZ – trnG-GCC*) | LSC |
|  | p1 | A | 10 | 38,068 | 38,077 | IGS (*trnG-GCC – trnfM-CAU*) | LSC |
|  | p1 | A | 10 | 43,755 | 43,764 | IGS (*psaA – ycf3*) | LSC |
|  | p1 | A | 10 | 46,650 | 46,659 | IGS (*ycf3 – trnS-GGA*) | LSC |
|  | p1 | A | 10 | 53,054 | 53,063 | IGS (*ndhC – trnV-UAC*) | LSC |
|  | p1 | A | 10 | 66,464 | 66,473 | *psbF* | LSC |
|  | p1 | A | 10 | 112,161 | 112,170 | *ycf1*; *ndhF* | IRb |
|  | p1 | A | 10 | 122,788 | 122,797 | *ndhA* | SSC |
|  | p1 | A | 10 | 130,477 | 130,486 | *ycf1* | IRa |
| 8 | p1 | A | 11 | 61,976 | 61,986 | IGS (*psaI – ycf4*) | LSC |
|  | p1 | A | 11 | 65,823 | 65,833 | IGS (*petA – psbJ*) | LSC |
|  | p1 | A | 11 | 72,916 | 72,926 | *clpP* | LSC |
|  | p1 | A | 11 | 119,280 | 119,290 | IGS (*ndhD – psaC*) | SSC |
|  | p1 | A | 11 | 125,892 | 125,902 | IGS (*rps15 – ycf1*) | SSC |
|  | p1 | A | 11 | 128,258 | 128,268 | *ycf1* | SSC |
|  | p1 | A | 11 | 129,481 | 129,491 | *ycf1* | SSC |
|  | p1 | A | 11 | 130,135 | 130,145 | *ycf1* | SSC |
| 12 | p1 | A | 12 | 77,287 | 77,298 | *petB* | LSC |
|  | p1 | A | 12 | 78,702 | 78,713 | *petD* | LSC |
| 4 | p1 | A | 13 | 378 | 390 | IGS (*trnH-GUG – psbA*) | LSC |
|  | p1 | A | 13 | 15,275 | 15,287 | IGS (*atpH – atpI*) | LSC |
|  | p1 | A | 13 | 49,052 | 49,064 | IGS (*trnT-UGU – trnL-UAA*) | LSC |
|  | p1 | A | 13 | 82,971 | 82,983 | IGS (*rpl14 – rpl16*) | LSC |
| 1 | p1 | A | 14 | 5,630 | 5,643 | *rps16* | LSC |
| 1 | p1 | A | 15 | 8,125 | 8,139 | IGS (*psbK – psbI*) | LSC |
| 1 | p1 | A | 16 | 115,077 | 115,092 | IGS (*ndhF – rpl32*) | SSC |
| 1 | p1 | A | 17 | 109,666 | 109,682 | IGS (*rrn5 – trnR-ACG*) | IRb |
| 1 | p1 | A | 22 | 8,843 | 8,864 | IGS (*trnS-GCU – trnG-UCC*) | LSC |
| 1 | p1 | A | 28 | 4,852 | 4,879 | IGS (*trnK-UUU – rps16*) | LSC |
| 1 | p1 | C | 10 | 50,454 | 50,463 | IGS (*trnF-GAA – ndhJ*) | LSC |
| 1 | p1 | C | 14 | 81,656 | 81,669 | *infA* | LSC |
| 1 | p1 | G | 10 | 68,024 | 68,033 | IGS (*petG – trnW-CCA*) | LSC |
| 14 | p1 | T | 10 | 13,078 | 13,087 | *atpF* | LSC |
|  | p1 | T | 10 | 14,929 | 14,938 | IGS (*atpH – atpI*) | LSC |
|  | p1 | T | 10 | 26,864 | 26,873 | *rpoB* | LSC |
|  | p1 | T | 10 | 48,605 | 48,614 | IGS (*trnT-UGU – trnL-UAA*) | LSC |
|  | p1 | T | 10 | 50,023 | 50,032 | IGS (*trnL-UAA – trnF-GAA*) | LSC |
|  | p1 | T | 10 | 56,668 | 56,677 | *atpB* | LSC |
|  | p1 | T | 10 | 69,012 | 69,021 | IGS (*psaJ – rpl33*) | LSC |
|  | p1 | T | 10 | 69,748 | 69,757 | IGS (*rpl33 – rps18*) | LSC |
|  | p1 | T | 10 | 72,262 | 72,271 | *clpP* | LSC |
|  | p1 | T | 10 | 79,700 | 79,709 | IGS (*petD – rpoA*) | LSC |
|  | p1 | T | 10 | 86,091 | 86,100 | *rps19* | LSC-IRb |
|  | p1 | T | 10 | 111,998 | 112,007 | *ycf1* | IRb |
|  | p1 | T | 10 | 115,787 | 115,796 | IGS (*rpl32 – trnL-UAG*) | SSC |
|  | p1 | T | 10 | 130,314 | 130,323 | *ycf1* | IRa |
| 11 | p1 | T | 11 | 17,297 | 17,307 | *rpoC2* | LSC |
|  | p1 | T | 11 | 28,352 | 28,362 | IGS (*rpoB – trnC-GCA*) | LSC |
|  | p1 | T | 11 | 30,301 | 30,311 | IGS (*psbM – trnD-GUC*) | LSC |
|  | p1 | T | 11 | 52,380 | 52,390 | IGS (*ndhK – ndhC*) | LSC |
|  | p1 | T | 11 | 53,779 | 53,789 | *trnV-UAC* | LSC |
|  | p1 | T | 11 | 67,104 | 67,114 | IGS (*psbE – petL*) | LSC |
|  | p1 | T | 11 | 115,672 | 115,682 | IGS (*rpl32 – trnL-UAG*) | SSC |
|  | p1 | T | 11 | 125,639 | 125,649 | *rps15* | SSC |
|  | p1 | T | 11 | 126,441 | 126,451 | *ycf1* | SSC |
|  | p1 | T | 11 | 128,842 | 128,852 | *ycf1* | SSC |
|  | p1 | T | 11 | 129,328 | 129,338 | *ycf1* | SSC |
| 2 | p1 | T | 12 | 9,124 | 9,135 | IGS (*trnS-GCU – trnG-UCC*) | LSC |
|  | p1 | T | 12 | 62,960 | 62,971 | IGS (*ycf4 – cemA*) | LSC |
| 3 | p1 | T | 13 | 30,111 | 30,123 | IGS (*petN – psbM*) | LSC |
|  | p1 | T | 13 | 44,804 | 44,816 | *ycf3* | LSC |
|  | p1 | T | 13 | 122,903 | 122,915 | *ndhA* | SSC |
| 2 | p1 | T | 14 | 19,210 | 19,223 | *rpoC2* | LSC |
|  | p1 | T | 14 | 23,617 | 23,630 | *rpoC1* | LSC |
| 1 | p1 | T | 15 | 8,258 | 8,272 | IGS (*psbK – psbI*) | LSC |
| 1 | p1 | T | 16 | 73,251 | 73,266 | *clpP* | LSC |
| 1 | p1 | T | 17 | 132,802 | 132,818 | IGS (*trnR-ACG – rrn5*) | IRa |
| 1 | p1 | T | 18 | 123,187 | 123,204 | *ndhA* | SSC |
| 1 | p2 | (AT)6 | 12 | 78,942 | 78,953 | *petD* | LSC |
| 1 | p2 | (AT)7 | 14 | 32,320 | 32,333 | IGS (*trnE-UUC – trnT-GGU*) | LSC |
| 1 | c | (A)10 ... (T)10 ... (A)12 | 114 | 115,424 | 115,537 | IGS (*rpl32 – trnL-UAG*) | SSC |
| 2 | c | (A)10 ... (T)11 | 57 | 61,785 | 61,841 | *psaI* | LSC |
|  | c | (A)10 ... (T)11 | 42 | 123,493 | 123,534 | *ndhA* | SSC |
| 1 | c | (T)10 ... (T)10 | 75 | 9,277 | 9,351 | IGS (*trnS-GCU – trnG-UCC*) | LSC |
| 1 | c | (T)10 ... (T)11 A (T)10 | 74 | 1,721 | 1,794 | IGS (*psbA – trnK-UUU*) | LSC |
| *Acer pseudoplatanus* | | | | | | |  |
| 10 | p1 | A | 10 | 6,394 | 6,403 | *rps16* | LSC |
|  | p1 | A | 10 | 13,911 | 13,920 | IGS (*atpF – atpH*) | LSC |
|  | p1 | A | 10 | 37,838 | 37,847 | IGS (*psbZ – trnG-GCC*) | LSC |
|  | p1 | A | 10 | 46,656 | 46,665 | IGS (*ycf3 – trnS-GGA*) | LSC |
|  | p1 | A | 10 | 61,571 | 61,580 | *psaI* | LSC |
|  | p1 | A | 10 | 66,236 | 66,245 | *psbF* | LSC |
|  | p1 | A | 10 | 72,671 | 72,680 | *clpP* | LSC |
|  | p1 | A | 10 | 125,472 | 125,481 | IGS (*rps15 – ycf1*) | SSC |
|  | p1 | A | 10 | 130,017 | 130,026 | *ycf1* | IRa |
|  | p1 | A | 10 | 141,325 | 141,334 | IGS (*ycf15 – rps12*) | IRa |
| 8 | p1 | A | 11 | 13,417 | 13,427 | *atpF* | LSC |
|  | p1 | A | 11 | 15,302 | 15,312 | IGS (*atpH – atpI*) | LSC |
|  | p1 | A | 11 | 37,433 | 37,443 | IGS (*psbZ – trnG-GCC*) | LSC |
|  | p1 | A | 11 | 65,599 | 65,609 | IGS (*petA – psbJ*) | LSC |
|  | p1 | A | 11 | 114,766 | 114,776 | IGS (*ndhF – rpl32*) | SSC |
|  | p1 | A | 11 | 127,828 | 127,838 | *ycf1* | SSC |
|  | p1 | A | 11 | 129,675 | 129,685 | *ycf1* | SSC |
|  | p1 | A | 11 | 131,939 | 131,949 | IGS (*trnN-GUU – trnR-ACG*) | IRa |
| 2 | p1 | A | 12 | 5,741 | 5,752 | *rps16* | LSC |
|  | p1 | A | 12 | 129,021 | 129,032 | *ycf1* | SSC |
| 4 | p1 | A | 13 | 8,923 | 8,935 | IGS (*trnS-GCU – trnG-UCC*) | LSC |
|  | p1 | A | 13 | 48,867 | 48,879 | IGS (*trnT-UGU – trnL-UAA*) | LSC |
|  | p1 | A | 13 | 73,885 | 73,897 | IGS (*clpP– psbB*) | LSC |
|  | p1 | A | 13 | 109,390 | 109,402 | IGS (*rrn5 – trnR-ACG*) | IRb |
| 3 | p1 | A | 14 | 33,404 | 33,417 | IGS (*trnT-GGU – psbD*) | LSC |
|  | p1 | A | 14 | 57,061 | 57,074 | IGS (*atpB – rbcL*) | LSC |
|  | p1 | A | 14 | 78,472 | 78,485 | *petD* | LSC |
| 14 | p1 | T | 10 | 12,514 | 12,523 | IGS (*atpA – atpF*) | LSC |
|  | p1 | T | 10 | 17,259 | 17,268 | *rpoC2* | LSC |
|  | p1 | T | 10 | 23,586 | 23,595 | *rpoC1* | LSC |
|  | p1 | T | 10 | 26,829 | 26,838 | *rpoB* | LSC |
|  | p1 | T | 10 | 30,095 | 30,104 | IGS (*petN – psbM*) | LSC |
|  | p1 | T | 10 | 53,605 | 53,614 | *trnV-UAC* | LSC |
|  | p1 | T | 10 | 56,492 | 56,501 | *atpB* | LSC |
|  | p1 | T | 10 | 69,492 | 69,501 | IGS (*rpl33 – rps18*) | LSC |
|  | p1 | T | 10 | 75,613 | 75,622 | IGS (*psbB – psbT*) | LSC |
|  | p1 | T | 10 | 100,412 | 100,421 | IGS (*rps12 – ycf15*) | IRb |
|  | p1 | T | 10 | 111,720 | 111,729 | *ycf1* | IRb |
|  | p1 | T | 10 | 118,965 | 118,974 | IGS (*ndhD – psaC*) | SSC |
|  | p1 | T | 10 | 126,607 | 126,616 | *ycf1* | SSC |
|  | p1 | T | 10 | 129,854 | 129,863 | *ycf1* | SSC-IRa |
| 10 | p1 | T | 11 | 9,191 | 9,201 | IGS (*trnS-GCU – trnG-UCC*) | LSC |
|  | p1 | T | 11 | 31,954 | 31,964 | IGS (*trnE-UUC – trnT-GGU*) | LSC |
|  | p1 | T | 11 | 49,848 | 49,858 | IGS (*trnL-UAA – trnF-GAA*) | LSC |
|  | p1 | T | 11 | 52,215 | 52,225 | IGS (*ndhK – ndhC*) | LSC |
|  | p1 | T | 11 | 72,011 | 72,021 | *clpP* | LSC |
|  | p1 | T | 11 | 109,797 | 109,807 | IGS (*trnR-ACG – trnN-GUU*) | IRb |
|  | p1 | T | 11 | 122,789 | 122,799 | *ndhA* | SSC |
|  | p1 | T | 11 | 125,230 | 125,240 | *rps15* | SSC |
|  | p1 | T | 11 | 128,400 | 128,410 | *ycf1* | SSC |
|  | p1 | T | 11 | 128,525 | 128,535 | *ycf1* | SSC |
| 1 | p1 | T | 12 | 122,506 | 122,517 | *ndhA* | SSC |
| 2 | p1 | T | 13 | 19,172 | 19,184 | *rpoC2* | LSC |
|  | p1 | T | 13 | 132,344 | 132,356 | IGS (*trnR-ACG – rrn5*) | IRa |
| 4 | p1 | T | 14 | 30,282 | 30,295 | IGS (*psbM – trnD-GUC*) | LSC |
|  | p1 | T | 14 | 48,454 | 48,467 | IGS (*trnT-UGU – trnL-UAA*) | LSC |
|  | p1 | T | 14 | 72,999 | 73,012 | *clpP* | LSC |
|  | p1 | T | 14 | 123,112 | 123,125 | *ndhA* | SSC |
| 2 | p2 | (AT)6 | 12 | 29,072 | 29,083 | IGS (*trnC-GCA – petN*) | LSC |
|  | p2 | (AT)6 | 12 | 32,295 | 32,306 | IGS (*trnE-UUC – trnT-GGU*) | LSC |
| 1 | p2 | (TA)8 | 16 | 78,713 | 78,728 | *petD* | LSC |
| 1 | c | (A)10 ... (A)11 | 44 | 53,263 | 53,306 | IGS (*ndhC – trnV-UAC*) | LSC |
| 1 | c | (AT)6 ... (A)10 | 76 | 4,809 | 4,884 | IGS (*trnK-UUU – rps16*) | LSC |
| 1 | c | (T)10 ... (A)13 | 79 | 66,885 | 66,963 | IGS (*psbE – petL*) | LSC |
| 2 | c | (T)10 ... (T)13 | 71 | 1,721 | 1,791 | IGS (*psbA – trnK-UUU*) | LSC |
|  | c | (T)10 ... (T)13 | 99 | 50,688 | 50,786 | IGS (*trnF-GAA – ndhJ*) | LSC |
| 1 | c | (T)11 ... (T)10 | 120 | 115,338 | 115,457 | IGS (*rpl32 – trnL-UAG*) | SSC |
| 1 | c | (T)13 ... (A)12 | 66 | 82,657 | 82,722 | IGS (*rpl14 – rpl16*) | LSC |
| *Acer rubrum* | | | | | | |  |
| 12 | p1 | A | 10 | 394 | 403 | IGS (*trnH-GUG – psbA*) | LSC |
|  | p1 | A | 10 | 5,674 | 5,683 | *rps16* | LSC |
|  | p1 | A | 10 | 7,847 | 7,856 | IGS (*psbK – psbI*) | LSC |
|  | p1 | A | 10 | 14,610 | 14,619 | IGS (*atpH – atpI*) | LSC |
|  | p1 | A | 10 | 52,432 | 52,441 | IGS (*ndhC – trnV-UAC*) | LSC |
|  | p1 | A | 10 | 61,162 | 61,171 | *psaI* | LSC |
|  | p1 | A | 10 | 65,801 | 65,810 | *psbF* | LSC |
|  | p1 | A | 10 | 69,519 | 69,528 | IGS (*rps18 – rpl20*) | LSC |
|  | p1 | A | 10 | 103,796 | 103,805 | *trnI-GAU* | IRb |
|  | p1 | A | 10 | 114,724 | 114,733 | IGS (*rpl32 – trnL-UAG*) | SSC |
|  | p1 | A | 10 | 122,083 | 122,092 | *ndhA* | SSC |
|  | p1 | A | 10 | 129,763 | 129,772 | *ycf1* | IRa |
| 9 | p1 | A | 11 | 3,689 | 3,699 | *trnK-UUU* | LSC |
|  | p1 | A | 11 | 36,776 | 36,786 | IGS (*psbZ – trnG-GCC*) | LSC |
|  | p1 | A | 11 | 61,351 | 61,361 | IGS (*psaI – ycf4*) | LSC |
|  | p1 | A | 11 | 65,179 | 65,189 | IGS (*petA – psbJ*) | LSC |
|  | p1 | A | 11 | 76,598 | 76,608 | *petB* | LSC |
|  | p1 | A | 11 | 127,544 | 127,554 | *ycf1* | SSC |
|  | p1 | A | 11 | 128,767 | 128,777 | *ycf1* | SSC |
|  | p1 | A | 11 | 129,421 | 129,431 | *ycf1* | SSC |
|  | p1 | A | 11 | 141,085 | 141,095 | IGS (*ycf15 – rps12*) | IRa |
| 3 | p1 | A | 12 | 48,400 | 48,411 | IGS (*trnT-UGU – trnL-UAA*) | LSC |
|  | p1 | A | 12 | 108,946 | 108,957 | IGS (*rrn5 – trnR-ACG*) | IRb |
|  | p1 | A | 12 | 114,380 | 114,391 | IGS (*ndhF – rpl32*) | SSC |
|  | p1 | A | 13 | 111,458 | 111,470 | *ycf1*;*ndhF* | IRb |
| 1 | p1 | A | 14 | 52,846 | 52,859 | IGS (*ndhC – trnV-UAC*) | LSC |
| 1 | p1 | A | 15 | 82,249 | 82,263 | IGS (*rpl14– rpl16*) | LSC |
| 1 | p1 | C | 10 | 49,825 | 49,834 | IGS (*trnF-GAA – ndhJ*) | LSC |
| 1 | p1 | C | 13 | 56,509 | 56,521 | IGS (*atpB – rbcL*) | LSC |
| 15 | p1 | T | 10 | 8,963 | 8,972 | IGS (*trnS-GCU – trnG-UCC*) | LSC |
|  | p1 | T | 10 | 11,818 | 11,827 | IGS (*atpA – atpF*) | LSC |
|  | p1 | T | 10 | 12,410 | 12,419 | *atpF* | LSC |
|  | p1 | T | 10 | 26,178 | 26,187 | *rpoB* | LSC |
|  | p1 | T | 10 | 27,660 | 27,669 | IGS (*rpoB – trnC-GCA*) | LSC |
|  | p1 | T | 10 | 31,868 | 31,877 | IGS (*trnT-GGU – psbD*) | LSC |
|  | p1 | T | 10 | 56,065 | 56,074 | *atpB* | LSC |
|  | p1 | T | 10 | 62,339 | 62,348 | IGS (*ycf4 – cemA*) | LSC |
|  | p1 | T | 10 | 66,441 | 66,450 | IGS (*psbE – petL*) | LSC |
|  | p1 | T | 10 | 66,727 | 66,736 | IGS (*psbE – petL*) | LSC |
|  | p1 | T | 10 | 68,331 | 68,340 | IGS (*psaJ – rpl33*) | LSC |
|  | p1 | T | 10 | 69,067 | 69,076 | IGS (*rpl33 – rps18*) | LSC |
|  | p1 | T | 10 | 111,295 | 111,304 | *ycf1* | IRb |
|  | p1 | T | 10 | 118,618 | 118,627 | IGS (*ndhD – psaC*) | SSC |
|  | p1 | T | 10 | 137,262 | 137,271 | *trnE-UUC* | IRa |
| 8 | p1 | T | 11 | 1,742 | 1,752 | IGS (*psbA – trnK-UUU*) | LSC |
|  | p1 | T | 11 | 14,266 | 14,276 | IGS (*atpH – atpI*) | LSC |
|  | p1 | T | 11 | 53,166 | 53,176 | *trnV-UAC* | LSC |
|  | p1 | T | 11 | 75,158 | 75,168 | IGS (*psbB – psbT*) | LSC |
|  | p1 | T | 11 | 99,972 | 99,982 | IGS (*rps12 – ycf15*) | IRb |
|  | p1 | T | 11 | 124,921 | 124,931 | *rps15* | SSC |
|  | p1 | T | 11 | 125,721 | 125,731 | *ycf1* | SSC |
|  | p1 | T | 11 | 128,128 | 128,138 | *ycf1* | SSC |
| 6 | p1 | T | 12 | 16,606 | 16,617 | *rpoC2* | LSC |
|  | p1 | T | 12 | 29,609 | 29,620 | IGS (*psbM – trnD-GUC*) | LSC |
|  | p1 | T | 12 | 72,578 | 72,589 | *clpP* | LSC |
|  | p1 | T | 12 | 114,972 | 114,983 | IGS (*rpl32 – trnL-UAG*) | SSC |
|  | p1 | T | 12 | 122,213 | 122,224 | *ndhA* | SSC |
|  | p1 | T | 12 | 132,110 | 132,121 | IGS (*trnR-ACG – rrn5*) | IRa |
| 2 | p1 | T | 13 | 71,595 | 71,607 | *clpP* | LSC |
|  | p1 | T | 13 | 129,597 | 129,609 | *ycf1* | IRa |
| 1 | p1 | T | 14 | 50,313 | 50,326 | IGS (*trnF-GAA – ndhJ*) | LSC |
| 1 | p1 | T | 16 | 51,753 | 51,768 | IGS (*ndhK – ndhC*) | LSC |
| 1 | p2 | (AT)9 | 18 | 31,654 | 31,671 | IGS (*trnE-UUC – trnT-GGU*) | LSC |
| 1 | c | (A)10 ... (T)10 | 41 | 122,769 | 122,809 | *ndhA* | SSC |
| *Acer sterculiaceum* subsp*. sterculiaceum* | | | | | | |  |
| 15 | p1 | A | 10 | 5,710 | 5,719 | *rps16* | LSC |
|  | p1 | A | 10 | 6,367 | 6,376 | *rps16* | LSC |
|  | p1 | A | 10 | 8,192 | 8,201 | IGS (*psbK – psbI*) | LSC |
|  | p1 | A | 10 | 8,874 | 8,883 | IGS (*trnS-GCU – trnG-UCC*) | LSC |
|  | p1 | A | 10 | 10,597 | 10,606 | IGS (*trnG-UCC – trnR-UCU*) | LSC |
|  | p1 | A | 10 | 43,716 | 43,725 | IGS (*psaA – ycf3*) | LSC |
|  | p1 | A | 10 | 52,981 | 52,990 | IGS (*ndhC – trnV-UAC*) | LSC |
|  | p1 | A | 10 | 57,165 | 57,174 | IGS (*atpB – rbcL*) | LSC |
|  | p1 | A | 10 | 61,698 | 61,707 | *psaI* | LSC |
|  | p1 | A | 10 | 66,358 | 66,367 | *psbF* | LSC |
|  | p1 | A | 10 | 78,661 | 78,670 | *petD* | LSC |
|  | p1 | A | 10 | 112,080 | 112,089 | *ycf1*;*ndhF* | IRb |
|  | p1 | A | 10 | 115,000 | 115,009 | IGS (*ndhF – rpl32*) | SSC |
|  | p1 | A | 10 | 130,356 | 130,365 | *ycf1* | IRa |
|  | p1 | A | 10 | 156,242 | 156,251 | IGS (*rpl2 – trnH-GUG*) | IRa |
| 7 | p1 | A | 11 | 377 | 387 | IGS (*trnH-GUG – psbA*) | LSC |
|  | p1 | A | 11 | 3,708 | 3,718 | *trnK-UUU* | LSC |
|  | p1 | A | 11 | 4,502 | 4,512 | IGS (*trnK-UUU – rps16*) | LSC |
|  | p1 | A | 11 | 46,616 | 46,626 | IGS (*ycf3– trnS-GGA*) | LSC |
|  | p1 | A | 11 | 65,717 | 65,727 | IGS (*petA – psbJ*) | LSC |
|  | p1 | A | 11 | 128,140 | 128,150 | *ycf1* | SSC |
|  | p1 | A | 11 | 130,005 | 130,015 | *ycf1* | SSC |
| 2 | p1 | A | 12 | 13,384 | 13,395 | *atpF* | LSC |
|  | p1 | A | 12 | 109,590 | 109,601 | IGS (*rrn5 – trnR-ACG*) | IRb |
| 3 | p1 | A | 13 | 48,982 | 48,994 | IGS (*trnT-UGU – trnL-UAA*) | LSC |
|  | p1 | A | 13 | 61,887 | 61,899 | IGS (*psaI – ycf4*) | LSC |
|  | p1 | A | 13 | 77,229 | 77,241 | *petB* | LSC |
| 2 | p1 | A | 14 | 53,394 | 53,407 | IGS (*ndhC – trnV-UAC*) | LSC |
|  | p1 | A | 14 | 61,076 | 61,089 | IGS (*accD – psaI*) | LSC |
| 1 | p1 | A | 15 | 15,281 | 15,295 | IGS (*atpH – atpI*) | LSC |
| 1 | p1 | A | 16 | 37,418 | 37,433 | IGS (*psbZ – trnG-GCC*) | LSC |
| 1 | p1 | C | 12 | 81,587 | 81,598 | *infA* | LSC |
| 1 | p1 | G | 10 | 67,918 | 67,927 | IGS (*petG – trnW-CCA*) | LSC |
| 18 | p1 | T | 10 | 7,692 | 7,701 | IGS (*trnQ-UUG – psbK*) | LSC |
|  | p1 | T | 10 | 8,320 | 8,329 | IGS (*psbK – psbI*) | LSC |
|  | p1 | T | 10 | 9,154 | 9,163 | IGS (*trnS-GCU – trnG-UCC*) | LSC |
|  | p1 | T | 10 | 9,306 | 9,315 | IGS (*trnS-GCU – trnG-UCC*) | LSC |
|  | p1 | T | 10 | 13,076 | 13,085 | *atpF* | LSC |
|  | p1 | T | 10 | 26,863 | 26,872 | *rpoB* | LSC |
|  | p1 | T | 10 | 30,127 | 30,136 | IGS (*petN – psbM*) | LSC |
|  | p1 | T | 10 | 56,605 | 56,614 | *atpB* | LSC |
|  | p1 | T | 10 | 60,916 | 60,925 | IGS (*accD – psaI*) | LSC |
|  | p1 | T | 10 | 69,684 | 69,693 | IGS (*rpl33 – rps18*) | LSC |
|  | p1 | T | 10 | 73,211 | 73,220 | *clpP* | LSC |
|  | p1 | T | 10 | 79,636 | 79,645 | IGS (*petD – rpoA*) | LSC |
|  | p1 | T | 10 | 86,022 | 86,031 | *rps19* | IRb |
|  | p1 | T | 10 | 111,908 | 111,917 | *ycf1* | IRb |
|  | p1 | T | 10 | 119,195 | 119,204 | IGS (*ndhD – psaC*) | SSC |
|  | p1 | T | 10 | 121,787 | 121,796 | IGS (*ndhI – ndhA*) | SSC |
|  | p1 | T | 10 | 128,838 | 128,847 | *ycf1* | SSC |
|  | p1 | T | 10 | 130,184 | 130,193 | *ycf1* | IRa |
| 5 | p1 | T | 11 | 66,998 | 67,008 | IGS (*psbE – petL*) | LSC |
|  | p1 | T | 11 | 75,772 | 75,782 | IGS (*psbB – psbT*) | LSC |
|  | p1 | T | 11 | 125,506 | 125,516 | *rps15* | SSC |
|  | p1 | T | 11 | 126,323 | 126,333 | *ycf1* | SSC |
|  | p1 | T | 11 | 128,712 | 128,722 | *ycf1* | SSC |
| 4 | p1 | T | 12 | 52,300 | 52,311 | IGS (*ndhK – ndhC*) | LSC |
|  | p1 | T | 12 | 53,706 | 53,717 | *trnV-UAC* | LSC |
|  | p1 | T | 12 | 115,589 | 115,600 | IGS (*rpl32 – trnL-UAG*) | SSC |
|  | p1 | T | 12 | 132,672 | 132,683 | IGS (*trnR-ACG – rrn5*) | IRa |
| 2 | p1 | T | 13 | 30,314 | 30,326 | IGS (*psbM – trnD-GUC*) | LSC |
|  | p1 | T | 13 | 50,858 | 50,870 | IGS (*trnF-GAA – ndhJ*) | LSC |
| 1 | p1 | T | 14 | 19,208 | 19,221 | *rpoC2* | LSC |
| 1 | p2 | (AT)6 | 12 | 32,327 | 32,338 | IGS (*trnE-UUC – trnT-GGU*) | LSC |
| 1 | c | (A)10 ... (A)10 | 118 | 4,845 | 4,962 | IGS (*trnK-UUU – rps16*) | LSC |
| 1 | c | (A)10 ... (T)10 | 41 | 123,344 | 123,384 | *ndhA* | SSC |
| 1 | c | (T)10 ... (A)11 | 113 | 129,249 | 129,361 | *ycf1* | SSC |
| 1 | c | (T)10 ... (T)12 | 103 | 72,122 | 72,224 | *clpP* | LSC |
| 1 | c | (T)11 ... (A)14 | 63 | 82,844 | 82,906 | IGS (*rpl14 – rpl16*) | LSC |
| *Acer tataricum* subsp*. ginnala* | | | | | | | |
| 16 | p1 | A | 10 | 4,466 | 4,475 | IGS (*trnK-UUU – rps16*) | LSC |
|  | p1 | A | 10 | 5,665 | 5,674 | *rps16* | LSC |
|  | p1 | A | 10 | 9,014 | 9,023 | IGS (*trnS-GCU – trnG-UCC*) | LSC |
|  | p1 | A | 10 | 13,819 | 13,828 | IGS (*atpF – atpH*) | LSC |
|  | p1 | A | 10 | 43,649 | 43,658 | IGS (*psaA – ycf3*) | LSC |
|  | p1 | A | 10 | 46,540 | 46,549 | IGS (*ycf3 – trnS-GGA*) | LSC |
|  | p1 | A | 10 | 52,394 | 52,403 | IGS (*ndhC – trnV-UAC*) | LSC |
|  | p1 | A | 10 | 61,093 | 61,102 | *psaI* | LSC |
|  | p1 | A | 10 | 61,280 | 61,289 | IGS (*psaI – ycf4*) | LSC |
|  | p1 | A | 10 | 65,738 | 65,747 | *psbF* | LSC |
|  | p1 | A | 10 | 83,048 | 83,057 | *rpl16* | LSC |
|  | p1 | A | 10 | 111,473 | 111,482 | *ycf1*;*ndhF* | IRb |
|  | p1 | A | 10 | 115,210 | 115,219 | IGS (*rpl32 – trnL-UAG*) | SSC |
|  | p1 | A | 10 | 122764 | 122,773 | *ndhA* | SSC |
|  | p1 | A | 10 | 128,757 | 128,766 | *ycf1* | SSC |
|  | p1 | A | 10 | 129,753 | 129,762 | *ycf1* | IRa |
| 7 | p1 | A | 11 | 400 | 410 | IGS (*trnH-GUG – psbA*) | LSC |
|  | p1 | A | 11 | 3,672 | 3,682 | *trnK-UUU* | LSC |
|  | p1 | A | 11 | 13,325 | 13,335 | *atpF* | LSC |
|  | p1 | A | 11 | 76,532 | 76,542 | *petB* | LSC |
|  | p1 | A | 11 | 114,387 | 114,397 | IGS (*ndhF – rpl32*) | SSC |
|  | p1 | A | 11 | 127,534 | 127,544 | *ycf1* | SSC |
|  | p1 | A | 11 | 129,411 | 129,421 | *ycf1* | SSC |
| 1 | p1 | A | 12 | 65,096 | 65,107 | IGS (*petA – psbJ*) | LSC |
| 1 | p1 | A | 13 | 8,817 | 8,829 | IGS (*trnS-GCU – trnG-UCC*) | LSC |
| 1 | p1 | A | 17 | 52,809 | 52,825 | IGS (*ndhC – trnV-UAC*) | LSC |
| 1 | p1 | A | 18 | 108,977 | 108,994 | IGS (*rrn5 – trnR-ACG*) | IRb |
| 1 | p1 | C | 13 | 80,920 | 80,932 | *infA* | LSC |
| 14 | p1 | T | 10 | 144 | 153 | IGS (*trnH-GUG – psbA*) | LSC |
|  | p1 | T | 10 | 9,524 | 9,533 | IGS (*trnS-GCU – trnG-UCC*) | LSC |
|  | p1 | T | 10 | 13,016 | 13,025 | *atpF* | LSC |
|  | p1 | T | 10 | 14,871 | 14,880 | IGS (*atpH – atpI*) | LSC |
|  | p1 | T | 10 | 26,808 | 26,817 | *rpoB* | LSC |
|  | p1 | T | 10 | 32,447 | 32,456 | IGS (*trnT-GGU – psbD*) | LSC |
|  | p1 | T | 10 | 44,690 | 44,699 | *ycf3* | LSC |
|  | p1 | T | 10 | 56,017 | 56,026 | *atpB* | LSC |
|  | p1 | T | 10 | 66,378 | 66,387 | IGS (*psbE – petL*) | LSC |
|  | p1 | T | 10 | 68,261 | 68,270 | IGS (*psaJ – rpl33*) | LSC |
|  | p1 | T | 10 | 111,310 | 111,319 | *ycf1* | IRb |
|  | p1 | T | 10 | 128,119 | 128,128 | *ycf1* | SSC |
|  | p1 | T | 10 | 128,244 | 128,253 | *ycf1* | SSC |
|  | p1 | T | 10 | 129,590 | 129,599 | *ycf1* | IRa |
| 8 | p1 | T | 11 | 1,725 | 1,735 | IGS (*psbA – trnK-UUU*) | LSC |
|  | p1 | T | 11 | 16,109 | 16,119 | IGS (*atpI – rps2*) | LSC |
|  | p1 | T | 11 | 51,699 | 51,709 | IGS (*ndhK – ndhC*) | LSC |
|  | p1 | T | 11 | 72,500 | 72,510 | *clpP* | LSC |
|  | p1 | T | 11 | 122,199 | 122,209 | *ndhA* | SSC |
|  | p1 | T | 11 | 122,481 | 122,491 | *ndhA* | SSC |
|  | p1 | T | 11 | 124,908 | 124,918 | *rps15* | SSC |
|  | p1 | T | 11 | 125,708 | 125,718 | *ycf1* | SSC |
| 1 | p1 | T | 13 | 30,239 | 30,251 | IGS (*psbM – trnD-GUC*) | LSC |
| 1 | p1 | T | 14 | 19,142 | 19,155 | *rpoC2* | LSC |
| 1 | p1 | T | 15 | 114,983 | 114,997 | IGS (*rpl32 – trnL-UAG*) | SSC |
| 1 | p1 | T | 18 | 132,078 | 132,095 | IGS (*trnR-ACG – rrn5*) | IRa |
| 1 | p2 | (AT)6 | 12 | 32,239 | 32,250 | IGS (*trnE-UUC – trnT-GGU*) | LSC |
| 1 | c | (A)10 ... (A)10 ... (A)10 | 178 | 4,737 | 4,914 | IGS (*trnK-UUU – rps16*) | LSC |
| 1 | c | (A)10 ... (A)12 | 119 | 114,730 | 114,848 | IGS (*rpl32 – trnL-UAG*) | SSC |
| 1 | c | (T)10 ... (A)19 | 68 | 82,168 | 82,235 | IGS (*rpl14 – rpl16*) | LSC |
| 1 | c | (T)10 ... (T)11 | 102 | 71,427 | 71,528 | *clpP* | LSC |
| 1 | c | (T)10 ... (T)13 | 106 | 50,157 | 50,262 | IGS (*trnF-GAA – ndhJ*) | LSC |
| 1 | c | (TAT)5 ... (T)11 | 123 | 53,018 | 53,140 | IGS (*ndhC – trnV-UAC*) | LSC |
