# Supplementary material for: Plastome comparative genomics in maples resolves the infrageneric backbone relationships
Source: PeerJ. 2020 Jul 13;8:e9483. doi: 10.7717/peerj.9483 (PMC7365138; doi:10.7717/peerj.9483)
Supplement: Table S6 — See Table S1 for full species names. [file peerj-08-9483-s006.docx]

**Table S6.** Pairwise percent identity for the 26 species included in the phylogenetic analysis. See Table S1 for full species names.

|  | ***A. acu*** | ***A. pilo*** | ***A. micr*** | ***A. plat*** | ***A. trun*** | ***A. miy*** | ***A. max*** | ***A. gris*** | ***A. oblo*** | ***A. pent*** | ***A. ster*** | ***A. pal*** | ***A. wils*** | ***A. negu*** | ***A. carp*** | ***A. tat*** | ***A. rubr*** | ***A. pseu*** | ***A. glab*** | ***A. nipp*** | ***A. davi*** | ***A. morr*** | ***D. sine*** | ***D. dyer*** | ***L. sine*** | ***S. mom*** |
| --- | --- | --- | --- | --- | --- | --- | --- | --- | --- | --- | --- | --- | --- | --- | --- | --- | --- | --- | --- | --- | --- | --- | --- | --- | --- | --- |
| ***A. acu*** |  | 97.4 | 97.6 | 97.5 | 97.4 | 97.1 | 97.1 | 95.2 | 96.9 | 96.6 | 97 | 97.4 | 97.2 | 97.4 | 96.9 | 97.5 | 97.2 | 97.1 | 97.2 | 97.4 | 95.3 | 95 | 96.2 | 95.8 | 84.5 | 83.9 |
| ***A. pilo*** | 97.4 |  | 97.8 | 97.4 | 97.3 | 97.1 | 97.1 | 95.3 | 96.9 | 96.6 | 96.8 | 97.6 | 97.2 | 97.7 | 97.1 | 97.5 | 97.5 | 97.7 | 96.9 | 97.3 | 95 | 94.7 | 96.1 | 95.7 | 84.4 | 83.7 |
| ***A. micr*** | 97.6 | 97.8 |  | 97.6 | 97.5 | 97.2 | 97.3 | 95.4 | 97.1 | 96.8 | 97.1 | 97.8 | 97.4 | 97.7 | 97.2 | 97.5 | 97.6 | 97.4 | 97 | 97.4 | 95.5 | 95.4 | 96.1 | 95.6 | 84.2 | 83.7 |
| ***A. plat*** | 97.5 | 97.4 | 97.6 |  | 99.2 | 99 | 97.9 | 94.9 | 97.8 | 97.5 | 97.6 | 97.4 | 96.9 | 97.3 | 96.7 | 97 | 97.2 | 97.1 | 97.8 | 97 | 94.7 | 94.4 | 95.7 | 95.3 | 84.9 | 84.3 |
| ***A. trun*** | 97.4 | 97.3 | 97.5 | 99.2 |  | 99.4 | 97.9 | 94.9 | 97.7 | 97.4 | 97.5 | 97.4 | 97 | 97.2 | 96.7 | 97 | 97.1 | 96.9 | 97.7 | 97 | 94.8 | 94.4 | 95.6 | 95.2 | 84.9 | 84.3 |
| ***A. miy*** | 97.1 | 97.1 | 97.2 | 99 | 99.4 |  | 97.6 | 94.7 | 97.5 | 97.1 | 97.3 | 97.1 | 96.8 | 96.9 | 96.4 | 96.7 | 96.8 | 96.7 | 97.4 | 96.7 | 94.5 | 94.1 | 95.4 | 95 | 84.7 | 84.1 |
| ***A. max*** | 97.1 | 97.1 | 97.3 | 97.9 | 97.9 | 97.6 |  | 96.9 | 98.8 | 98.2 | 97.3 | 97.1 | 96.7 | 97 | 96.6 | 96.9 | 96.9 | 96.8 | 97.5 | 96.8 | 94.4 | 94.2 | 95.5 | 95.1 | 84.8 | 84.2 |
| ***A. gris*** | 95.2 | 95.3 | 95.4 | 94.9 | 94.9 | 94.7 | 96.9 |  | 95.7 | 95.1 | 94.4 | 95.1 | 95.5 | 95.1 | 94.8 | 95 | 95 | 95 | 94.5 | 94.9 | 97.4 | 97.1 | 94.6 | 94.2 | 85.8 | 82 |
| ***A. oblo*** | 96.9 | 96.9 | 97.1 | 97.8 | 97.7 | 97.5 | 98.8 | 95.7 |  | 98 | 97.2 | 96.9 | 96.5 | 97 | 96.4 | 96.7 | 96.7 | 96.7 | 97.4 | 96.6 | 94.2 | 93.9 | 95.2 | 94.9 | 84.6 | 84.1 |
| ***A. pent*** | 96.6 | 96.6 | 96.8 | 97.5 | 97.4 | 97.1 | 98.2 | 95.1 | 98 |  | 97.7 | 96.7 | 96.2 | 96.6 | 96.2 | 96.4 | 96.4 | 96.3 | 97.1 | 96.3 | 93.9 | 93.7 | 95 | 94.6 | 84.3 | 83.8 |
| ***A. ster*** | 97 | 96.8 | 97.1 | 97.6 | 97.5 | 97.3 | 97.3 | 94.4 | 97.2 | 97.7 |  | 97 | 96.5 | 96.8 | 96.5 | 96.7 | 96.7 | 96.6 | 97.3 | 96.6 | 94.4 | 94.1 | 95.4 | 95 | 84.4 | 83.9 |
| ***A. pal*** | 97.4 | 97.6 | 97.8 | 97.4 | 97.4 | 97.1 | 97.1 | 95.1 | 96.9 | 96.7 | 97 |  | 98.8 | 97.5 | 97 | 97.3 | 97.5 | 97.3 | 97 | 97.2 | 95 | 94.8 | 96 | 95.6 | 84.2 | 83.6 |
| ***A. wils*** | 97.2 | 97.2 | 97.4 | 96.9 | 97 | 96.8 | 96.7 | 95.5 | 96.5 | 96.2 | 96.5 | 98.8 |  | 97.2 | 96.7 | 96.9 | 97.1 | 96.9 | 96.5 | 96.8 | 95.3 | 95.2 | 96.2 | 95.8 | 83.9 | 83.3 |
| ***A. negu*** | 97.4 | 97.7 | 97.7 | 97.3 | 97.2 | 96.9 | 97 | 95.1 | 97 | 96.6 | 96.8 | 97.5 | 97.2 |  | 97.1 | 97.2 | 97.5 | 97.4 | 96.9 | 97.3 | 94.9 | 94.5 | 96 | 95.6 | 84.1 | 83.6 |
| ***A. carp*** | 96.9 | 97.1 | 97.2 | 96.7 | 96.7 | 96.4 | 96.6 | 94.8 | 96.4 | 96.2 | 96.5 | 97 | 96.7 | 97.1 |  | 96.9 | 96.9 | 96.8 | 96.5 | 97 | 94.6 | 94.3 | 95.7 | 95.1 | 84.2 | 83.4 |
| ***A. tat*** | 97.5 | 97.5 | 97.5 | 97 | 97 | 96.7 | 96.9 | 95 | 96.7 | 96.4 | 96.7 | 97.3 | 96.9 | 97.2 | 96.9 |  | 97.1 | 97.2 | 96.8 | 97.2 | 94.9 | 94.6 | 96 | 95.5 | 84.3 | 83.7 |
| ***A. rubr*** | 97.2 | 97.5 | 97.6 | 97.2 | 97.1 | 96.8 | 96.9 | 95 | 96.7 | 96.4 | 96.7 | 97.5 | 97.1 | 97.5 | 96.9 | 97.1 |  | 97.2 | 96.7 | 97.1 | 94.8 | 94.5 | 95.9 | 95.5 | 84.2 | 83.6 |
| ***A. pseu*** | 97.1 | 97.7 | 97.4 | 97.1 | 96.9 | 96.7 | 96.8 | 95 | 96.7 | 96.3 | 96.6 | 97.3 | 96.9 | 97.4 | 96.8 | 97.2 | 97.2 |  | 97.2 | 97 | 94.7 | 94.4 | 96 | 95.6 | 84.3 | 83.6 |
| ***A. glab*** | 97.2 | 96.9 | 97 | 97.8 | 97.7 | 97.4 | 97.5 | 94.5 | 97.4 | 97.1 | 97.3 | 97 | 96.5 | 96.9 | 96.5 | 96.8 | 96.7 | 97.2 |  | 96.9 | 94.5 | 94.2 | 95.8 | 95.3 | 84.9 | 84.4 |
| ***A. nipp*** | 97.4 | 97.3 | 97.4 | 97 | 97 | 96.7 | 96.8 | 94.9 | 96.6 | 96.3 | 96.6 | 97.2 | 96.8 | 97.3 | 97 | 97.2 | 97.1 | 97 | 96.9 |  | 94.9 | 94.5 | 95.9 | 95.4 | 84.2 | 83.7 |
| ***A. davi*** | 95.3 | 95 | 95.5 | 94.7 | 94.8 | 94.5 | 94.4 | 97.4 | 94.2 | 93.9 | 94.4 | 95 | 95.3 | 94.9 | 94.6 | 94.9 | 94.8 | 94.7 | 94.5 | 94.9 |  | 97.7 | 94.8 | 94.4 | 85.7 | 82.1 |
| ***A. morr*** | 95 | 94.7 | 95.4 | 94.4 | 94.4 | 94.1 | 94.2 | 97.1 | 93.9 | 93.7 | 94.1 | 94.8 | 95.2 | 94.5 | 94.3 | 94.6 | 94.5 | 94.4 | 94.2 | 94.5 | 97.7 |  | 94.4 | 94 | 85.5 | 81.9 |
| ***D. sine*** | 96.2 | 96.1 | 96.1 | 95.7 | 95.6 | 95.4 | 95.5 | 94.6 | 95.2 | 95 | 95.4 | 96 | 96.2 | 96 | 95.7 | 96 | 95.9 | 96 | 95.8 | 95.9 | 94.8 | 94.4 |  | 96 | 83.9 | 83.4 |
| ***D. dyer*** | 95.8 | 95.7 | 95.6 | 95.3 | 95.2 | 95 | 95.1 | 94.2 | 94.9 | 94.6 | 95 | 95.6 | 95.8 | 95.6 | 95.1 | 95.5 | 95.5 | 95.6 | 95.3 | 95.4 | 94.4 | 94 | 96 |  | 83.7 | 83.2 |
| ***L. sine*** | 84.5 | 84.4 | 84.2 | 84.9 | 84.9 | 84.7 | 84.8 | 85.8 | 84.6 | 84.3 | 84.4 | 84.2 | 83.9 | 84.1 | 84.2 | 84.3 | 84.2 | 84.3 | 84.9 | 84.2 | 85.7 | 85.5 | 83.9 | 83.7 |  | 82.7 |
| ***S. mom*** | 83.9 | 83.7 | 83.7 | 84.3 | 84.3 | 84.1 | 84.2 | 82 | 84.1 | 83.8 | 83.9 | 83.6 | 83.3 | 83.6 | 83.4 | 83.7 | 83.6 | 83.6 | 84.4 | 83.7 | 82.1 | 81.9 | 83.4 | 83.2 | 82.7 |  |
